# *Dusp6* deficiency attenuates neutrophil-mediated cardiac damage in the acute inflammatory phase of myocardial infarction

**DOI:** 10.1038/s41467-022-33631-z

**Published:** 2022-11-05

**Authors:** Xiaohai Zhou, Chenyang Zhang, Xueying Wu, Xinli Hu, Yan Zhang, Xuelian Wang, Lixia Zheng, Peng Gao, Jianyong Du, Wen Zheng, Haibao Shang, Keping Hu, Zhengfan Jiang, Yu Nie, Shengshou Hu, Rui-Ping Xiao, Xiaojun Zhu, Jing-Wei Xiong

**Affiliations:** 1 Beijing Key Laboratory of Cardiometabolic Molecular Medicine, Institute of Molecular Medicine, College of Future Technology, and State Key Laboratory of Natural and Biomimetic Drugs, Peking University, Beijing, 100871 China; 2 PKU-Nanjing Institute of Translational Medicine, Nanjing, 211800 China; 3 Research Center for Pharmacology and Toxicology, Institute of Medicinal Plant Development, Chinese Academy of Medical Sciences and Peking Union Medical College, Beijing, 100193 China; 4 School of Life Sciences, Peking University, Beijing, 100871 China; 5 State Key Laboratory of Cardiovascular Disease, Fuwai Hospital, National Center for Cardiovascular Disease, Chinese Academy of Medical Sciences and Peking Union Medical College, Beijing, 100037 China

**Keywords:** Inflammation, Cardiovascular biology, Transcription

## Abstract

Dual-specificity phosphatase 6 (DUSP6) serves a specific and conserved function on the dephosphorylation of extracellular signal-regulated kinase 1/2 (ERK1/2). We previously identified Dusp6 as a regenerative repressor during zebrafish heart regeneration, therefore we propose to investigate the role of this repressor in mammalian cardiac repair. Utilizing a rat strain harboring *Dusp6* nonsense mutation, rat neutrophil-cardiomyocyte co-culture, bone marrow transplanted rats and neutrophil-specific *Dusp6* knockout mice, we find that *Dusp6* deficiency improves cardiac outcomes by predominantly attenuating neutrophil-mediated myocardial damage in acute inflammatory phase after myocardial infarction. Mechanistically, *Dusp6* is transcriptionally activated by p38-C/EBPβ signaling and acts as an effector for maintaining p-p38 activity by down-regulating pERK and p38-targeting phosphatases DUSP1/DUSP16. Our findings provide robust animal models and novel insights for neutrophil-mediated cardiac damage and demonstrate the potential of DUSP6 as a therapeutic target for post-MI cardiac remodeling and other relevant inflammatory diseases.

## Abstract (web summary)

Dusp6, an ERK specific phosphatase, was identified as a regenerative repressor during zebrafish heart regeneration. Here, the authors show that *Dusp6* deficiency improves post infarctional cardiac repair by predominantly attenuating neutrophil-mediated myocardial damage in mammalian hearts.

## Introduction

Over the past 30 years, advances in the development of early reperfusion strategies by percutaneous coronary intervention as well as pharmacological prevention and intervention have alleviated the overall mortality in patients with myocardial infarction (MI). However, due to the limited regenerative capacity of the adult human heart, the necrotic cardiac tissue is irreversibly replaced by fibrotic scars rather than newly-generated cardiomyocytes, thus leading to the pathogenesis of cardiac remodeling, which consists of progressive pathological changes in the size, shape, function, and cellular and molecular composition of the left ventricle (LV)^1^. This remodeling process contributes to the development of cardiac fibrosis and other adverse outcomes, eventually leading to heart failure and death^2^. In clinical practice, percutaneous coronary intervention is the primary means of controlling the extent of infarcted myocardium and thus lessening the severity of post-MI cardiac remodeling. Nonetheless, recent preclinical studies have focused on a large number of potential therapeutic targets that may favorably influence autogenous cardiac wound healing and repair^1,3^.

Cardiac repair after MI involves an early inflammatory phase with intense aseptic inflammation and immune cell infiltration that serves to digest and clear extracellular matrix tissue and damaged cells (~1–4 days after MI in mice), and a reparative phase of resolving inflammation accompanied by fibroblast proliferation and activation, scar formation, and neovascularization^2^. Early inflammatory activation is a necessary event for the transition to the later reparative programs. Appropriate control of the resolution of inflammation is a key determinant to wound healing, thus highlighting that a proper physiological balance between these two phases is needed to achieve optimal repair. Either excessive or insufficient suppression of inflammatory processes during the post-MI stage leads to additional cardiac damage and deferred repair, thereby promoting infarct expansion, adverse remodeling, and functional loss^4^. So far, it remains difficult to translate immunomodulatory and anti-inflammatory therapeutic strategies into clinical practice, except for antibody against IL-1β (canakinumab) for the treatment of post-MI patients^5^, suggesting that our understanding of the relevant fundamental biology and pathology is still incomplete.

Zebrafish and newt have great potential for adult heart regeneration^6–8^. We have previously shown that H_2_O_2_ produced by NADPH oxidases promotes zebrafish heart regeneration by activating Erk1/2, and Dusp6 (dual-specificity phosphatase 6, also called Mkp3) acts as a direct target of H_2_O_2_ to down-regulate pERK and impair heart regeneration^9^. As a member of the dual-specificity phosphatase family, DUSP6 protein consists of an N-terminal non-catalytic domain with a kinase interaction motif and a C-terminal phosphatase domain that catalyzes threonine/tyrosine dephosphorylation. The kinase interaction motif has great substrate selectivity and mediates the binding of DUSP6 to the conserved common docking domain in ERK1 and ERK2 rather than other MAPKs. This interaction is indispensable for activation of the C-terminal catalytic domain, leading to specific dephosphorylation activity of DUSP6 against ERK1/2^10,11^. The function of DUSP6 has been well studied in organ development^12^, tumor suppression^13^, metabolic diseases^14^ and immune diseases^15,16^. Inducible expression of DUSP6 in cardiac tissue has no effect on the hypertrophic response to pressure-overload stimulation, but promotes stress-induced apoptosis and heart failure^17^. *Dusp6*-knockout mice are reported to have improved cardiac function and reduced scar formation after MI^18^, but the underlying cellular and molecular mechanisms remain largely unknown.

Here, by using CRISPR-induced *Dusp6* mutant rats as reported previously^19^, as well as a newly established neutrophil-specific Dusp6-knockout mouse model, we found that *Dusp6* deficiency improves cardiac repair and function by predominantly attenuating neutrophil activity at the acute inflammatory phase post-MI while having no effect on neutrophil development, differentiation, and injury-induced tissue infiltration. In addition, DUSP6 acts as a key effector and sustainer of p38 activity by inhibiting pERK in neutrophils. This work reveals DUSP6 as a novel rheostat to regulate p38 MAPK-dependent neutrophil activity, thus providing a powerful animal model to investigate the potential of pharmacological interventions targeting neutrophil-mediated tissue damage for post-MI cardiac remodeling and other relevant human diseases.

## Results

### *Dusp6* deficiency improves post-MI cardiac outcomes in rats

Rats have been utilized as an ideal animal model for cardiovascular diseases due to their larger heart size and blood volume, as well as their higher similarities to human cardiac physiology than that of mice^20^. The development of CRISPR/Cas9 system enables gene targeting in rats and thus makes the biomedical community to fully take advantage of the aforementioned benefits that rats can offer^21^. We previously used the CRISPR/Cas9 system to create a series of rat mutant strains that have various insertions or deletions in the *Dusp6* locus^19^. One of these mutants had a premature stop codon (TAA) resulting from the insertion of an adenine nucleotide into exon 1 of the *Dusp6* gene, and this led to the interruption of DUSP6 protein translation (Supplementary Fig. 1a). Homozygous *Dusp6* mutant rats had no evident abnormality in development, reproduction, life span, or behavior. Western blot revealed the complete absence of DUSP6 proteins in the adult heart, liver, spleen, and lung from *Dusp6* mutant rats, accompanied with enhanced phosphorylation of ERK1/2 (pERK), a specific substrate of DUSP6 (Supplementary Fig. 1b–i). However, due to the very low-level DUSP6 proteins in wild-type (WT) hearts (Supplementary Fig. 1b), the pERK levels in WT and *Dusp6*-deficient hearts were comparable (Supplementary Fig. 1b, c), which is consistent with our previous findings of undetectable Dusp6 protein expression and function in normal adult zebrafish heart^9^.

**Fig. 1:**
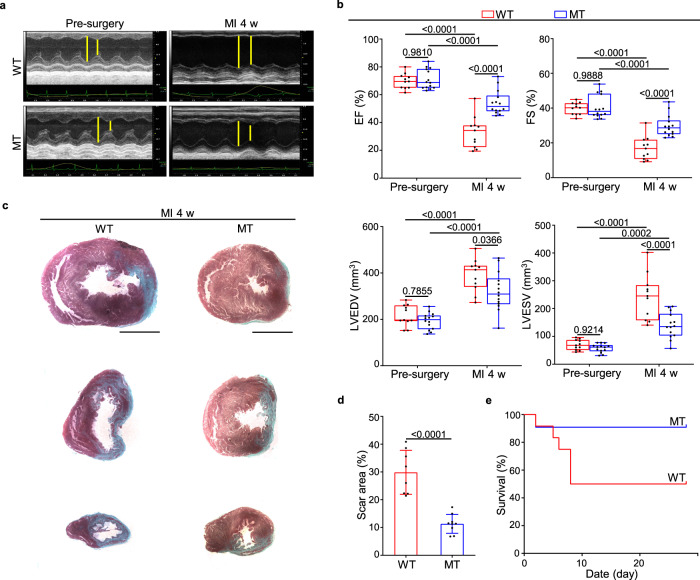
Reduced ventricular remodeling and improved cardiac function in *Dusp6*-deficient rats after MI. **a** Representative echocardiographic images from WT (*n* = 11) and *Dusp6*-deficient rats (*n* = 14) before surgery and 4 weeks (4 w) after myocardial infarction (MI). **b** Measurement of cardiac function indexes EF and FS as well as the left ventricular dilatation indexes LVEDV and LVESV from echocardiographic results as in **a**. The box blots show center lines as median, box boundaries as upper and lower quartiles, and whiskers as minimum and maximum values. Masson staining of heart sections (**c**) and quantitative analysis (**d**) of the fibrotic area from WT (*n* = 8) and *Dusp6*-deficient hearts (*n* = 8) at 4w after MI. Scale bars: 5 mm. **e** Kaplan–Meier survival curves of WT (*n* = 11) and *Dusp6*-deficient rats (*n* = 12) at 4w after MI. Quantitative data are presented as min to max with all points mean in **b**, and as values ± SD in **d** and **e**. One-way ANOVA with Tukey’s multiple comparison test (for **b**) and Two-sided unpaired T-test (for **d**) were used to calculate the presented *p*-values. Source data of **b**, **d** and **e** are provided in a Source Data File. MI myocardial infarction, WT wild-type, MT *Dusp6* mutant, EF ejection fraction, FS fractional shortening, LVEDV left ventricular end-diastolic volume, LVESV left ventricular end-systolic volume.

To examine the basal characteristics of *Dusp6*-deficient rats, we measured the weights of body and multiple organs in WT and *Dusp6*-deficient rats at 3 months of age, and found evidently increased spleen/body weight ratio, as well as slightly increased heart/body weight ratio in *Dusp6*-deficient rats (Supplementary Fig. 1j and Supplementary Table 2). However, the numbers of cardiomyocytes in LV tissues, as well as LV mass and anterior/posterior dimension were not enhanced in *Dusp6*-deficient rats (Supplementary Fig. 2), suggesting that LV was not implicated in the increase of heart weight in *Dusp6*-deficient rats. To test whether DUSP6 also acts as a negative regulator during mammalian cardiac repair, we took advantage of the *Dusp6* mutant line and induced myocardial infarction (MI) by the occlusion of the left anterior descending artery (LAD) as previously described^22^. Echocardiographic imaging revealed that both cardiac morphology and function were comparable in WT and *Dusp6*-deficient rats before MI, indicating that the slight increase of *Dusp6*-deficient heart weight is not pathological. In contrast, the *Dusp6*-deficient hearts had better systolic performance and smaller cardiac dilatation than the WT sibling hearts at 4 weeks after MI, as indicated by better preserved ejection fraction and fractional shortening as well as smaller LV end-diastolic volume and end-systolic volume (Fig. 1a, b and Supplementary Table 3). Masson’s trichrome staining showed that fibrotic scar formation in the *Dusp6*-deficient hearts decreased compared with that in the WT hearts (Fig. 1c, d). Consistently, the post-MI survival rate of *Dusp6*-deficient rats was higher than that of WT rats (Fig. 1e). These results suggest that *Dusp6* deficiency markedly represses LV remodeling and improves cardiac function after MI.

### *Dusp6* deficiency leads to reduced cardiac damage at inflammatory stage after MI

**Fig. 2:**
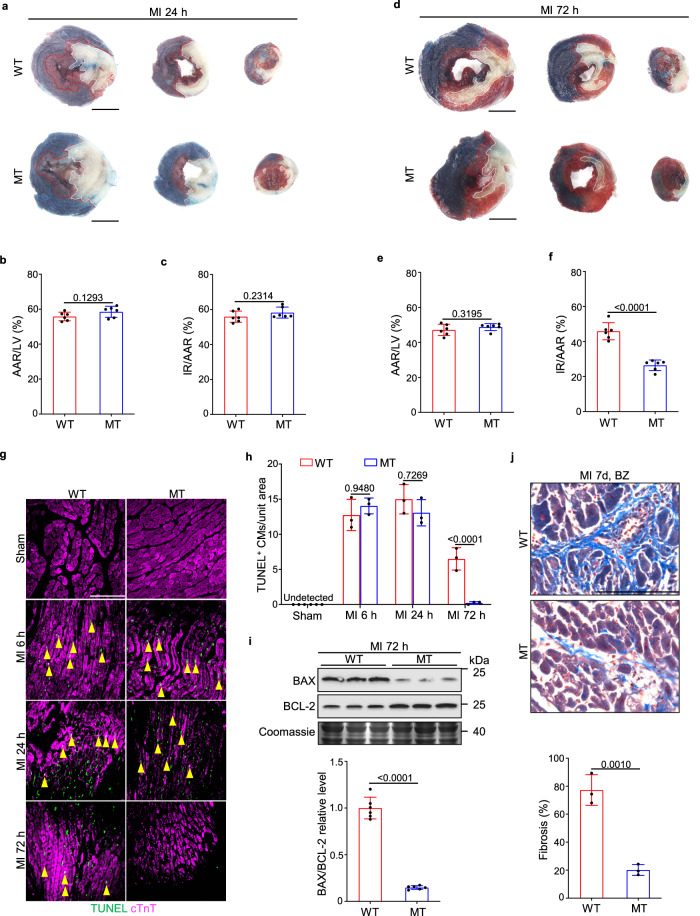
*Dusp6* deficiency reduces cardiac cell death and interstitial fibrosis at post-acute stage. **a**–**f** Representative TTC-Evans blue staining (**a** and **d**) and measurements of infarct size in WT and *Dusp6*-deficient hearts at 24 h (**b** and **c**) and 72 h (**e** and **f**) after MI (*n* = 6 hearts/group). In the ischemic area, the infarcted region is stained white and viable cardiac tissue is stained red. Non-ischemic heart muscle is stained blue (scale bar, 5 mm). Representative TUNEL staining of WT and *Dusp6* mutant hearts in sham-operated, MI 6 h, MI 24 h, and MI 72 h (**g**), and quantitative analysis (**h**) of cardiomyocyte (CM) cell death in WT and *Dusp6*-deficient LV tissue. Immunostaining of cTnT was used to co-stain myocardial tissue (*n* = 9 areas from 3 sham WT hearts and 9 areas from 3 sham MT hearts, 14 areas from WT hearts and 11 areas from MT hearts at 6 h after MI, 17 areas from WT hearts and 17 areas from MT hearts at 24 h after MI, 12 areas from WT hearts and 9 areas from MT hearts at 72 h after MI; scale bar, 100 μm). **i** Western blot and corresponding quantitative analysis of BAX and BCL-2 from WT and *Dusp6*-deficient LV tissue at 72 h after MI (*n* = 6 biological independent samples/group). Coomassie blue staining was used to normalize protein loading. All blots and stainings were performed in parallel with the same samples. **j** Masson staining and quantitative analysis of interstitial fibrosis in the infarct border zone of WT and *Dusp6*-deficient hearts at 7 days after MI (*n* = 9 areas from WT hearts and 9 areas from MT hearts; scale bar, 100 μm). All quantitative data shown in this figure are presented as mean values ± SD. One-way ANOVA with Tukey’s multiple comparison test (for **h**) and Two-sided unpaired T-test (for **b**, **c**, **e**, **f**, **i** and **j**) were used to calculate the presented *p*-values. Source data of **b**, **c, e, f, h, i** and **j** are provided in a Source Data File. WT wild-type, MT *Dusp6* mutant, TTC triphenyl tetrazolium chloride, IR infarcted region, AAR area at risk, LV left ventricle, BZ infarct border zone, cTnT cardiac troponin T.

Infarct size is the dominant factor for determining the level of LV remodeling after MI. Thus, we measured the infarcted region (IR) at various stages after MI using triphenyl tetrazolium chloride (TTC)-Evans blue staining. The areas of ischemic myocardial tissue (area at risk, AAR) assessed by Evans blue perfusion were similar in WT and *Dusp6*-deficient hearts at 24 h and 72 h after MI (Fig. 2a, b, d, e), suggesting that the level of ischemia induced by LAD occlusion was consistent among WT and mutant groups. The acute ischemia-induced cardiac injury was comparable in the WT and *Dusp6*-deficient hearts as shown by equivalent IR size at 24 h after MI (Fig. 2a, c). However, at 72 h after MI, the infarcted area was markedly reduced by *Dusp6* deficiency (Fig. 2d, f). With neovascularization and blood-supply restoration at 72 h after MI, ischemia-induced necrosis of CMs is repressed to some degrees while the clearance of dead CMs by infiltrated inflammatory cells launched and the necrotic area is gradually replaced by newly formed granulation tissues, which is the prerequisite for healing and scar formation in the inflammatory phase. The formation of granulation tissues, as well as the shrinkage of necrotic tissues and hypertrophy of residual myocardium, leads to no further enlargement but a reduction of infarct size from 24 to 72 h after MI as observed by TTC-Evans blue staining (Fig. 2a–f)^23^. Nevertheless, inflammation, reactive oxygen species (ROS) stress, and other factors further prompt progressive cardiac damage in the granulation tissues at the border zone of the infarcted area^2^. Therefore, the attenuated IR/AAR at 72 h rather than 24 h post-MI suggests that the improvement of post-MI cardiac function by *Dusp6* deficiency is due to a reduction of progressive cardiac damage in the inflammatory phase. Furthermore, Wheat Germ Agglutinin (WGA) staining revealed comparable areas of CMs at the border zone between WT and *Dusp6*-deficient LV tissues 72 h after MI, indicating that the reduced IR in *Dusp6*-deficient hearts at this stage is not owing to enhanced CM hypertrophy (Supplementary Fig. 3a, b).

We then performed TUNEL staining at various time points after MI and found comparable numbers of TUNEL-positive CMs in WT and *Dusp6*-deficient LV tissues within 24 h after MI. Conversely, at 72 h after MI, the numbers of TUNEL-positive CMs dramatically decreased in the *Dusp6*-deficient heart (Fig. 2g, h). Consistently, Western blot analysis showed a reduced level of BAX, a pro-apoptotic protein of the BCL-2 family, but an elevated level of the anti-apoptotic protein BCL-2 in lysates from the *Dusp6*-deficient LV at 72 h after MI (Fig. 2i), suggesting that *Dusp6* deficiency indeed attenuates the CM death at this stage. Furthermore, the degree of interstitial fibrosis in the infarct border zone, another hallmark of LV remodeling, was also attenuated in the *Dusp6*-deficient heart (Fig. 2j). Taken together, *Dusp6* ablation significantly reduces progressive LV damage in acute inflammatory phase while has no protective effect on the early ischemia-induced myocardial necrosis and apoptosis.

In line with the results of TTC-Evans blue staining (Fig. 2a, c) and TUNEL assay (Fig. 2g, h) within 24 h after MI, lactate dehydrogenase (LDH) release assay showed that H_2_O_2_-induced CM death was comparable between WT and *Dusp6* mutant neonatal rat ventricular myocytes (NRVMs) (Supplementary Fig. 3c), suggesting that *Dusp6* deficiency has no intrinsic effects on CM viability upon lethal stress. By performing immunofluorescence staining with antibodies against the mitosis marker phosphorylated histone H3 (pH3) and cardiac troponin T (cTnT), we found no contribution of proliferative CMs to the reduced IR in the *Dusp6*-deficient heart at 72 h after MI (Supplementary Fig. 3d). Compensatory collateral angiogenesis in the infarct area also contributes to post-MI cardiac repair and improves heart function^24^. By double immunofluorescence for CD31 and α-smooth muscle actin-positive (α-SMA), we found comparable densities of coronary arteries (CD31^+^/α-SMA^+^) and capillaries (CD31^+^/α-SMA^−^) between WT and *Dusp6*-deficient hearts at sham-operated and post-MI (Supplementary Fig. 3e, f). Therefore, the reduced cardiac damage in *Dusp6*-deficienct rats at 72 h post-MI is not due to intrinsic changes in CMs and vessels.

### *Dusp6* mRNA and protein are enriched in neutrophils

**Fig. 3:**
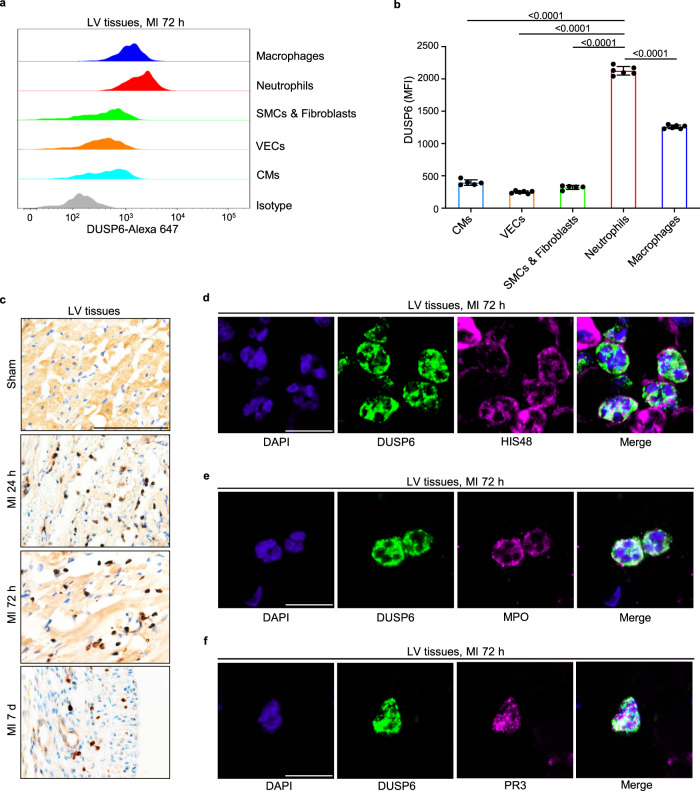
DUSP6 is predominantly enriched in infiltrated neutrophils after MI. **a**, **b** Intracellular staining, flow cytometry and corresponding quantitative analysis of DUSP6 levels in various cell types in wild-type LV tissue at 72 h after MI. *n* = 5 (CMs, SMCs & Fibroblasts) or 6 (VECs, Neutrophils, Macrophages) biological independent samples/group. Gating strategies for each cell population are shown in Supplementary Fig. 4a**. c** Representative immunohistochemistry of DUSP6 (dark brown) in wild-type LV tissue at sham operation, 24 h, 72 h, and 7 days after MI (*n* = 9 areas from 3 hearts/group; scale bar, 100 μm). **d–f** Representative double immunofluorescence of DUSP6 with either HIS48, MPO, or PR3 in wild-type LV tissue at 72 h after MI, showing DUSP6 expression in neutrophils (*n* = 9 areas from 3 hearts/group; scale bar, 10 μm). DAPI co-staining was used to display nuclear morphology. All quantitative data shown in this figure are presented as mean values ± SD. One-way ANOVA with Tukey’s multiple comparison test was used to calculate the presented p-values. Source data of **b** are provided in a Source Data File. CMs cardiomyocytes, VECs vascular endothelial cells, SMCs smooth muscle cells, MPO myeloperoxidase, PR3 proteinase 3, MFI median fluorescence intensity.

The above results implied that the ablation of DUSP6 in non-cardiac tissue and cell types might contribute to the improvement of post-MI cardiac function. However, the function and DUSP6-expressing cardiac cell types in the MI pathology remain unexplored. We first investigated the expression pattern of *Dusp6* in the infarcted heart. Flow cytometry analysis of wild-type LV tissues at 72 h after MI with DUSP6 antibody (Abcam, ab76310) revealed that DUSP6 protein was highly expressed in neutrophils and macrophages, and was most abundant in neutrophils among all cells examined (Fig. 3a, b and Supplementary Fig. 4a). DUSP6 signal was largely reduced in *Dusp6*-deficient cells (Supplementary Fig. 5a, b), confirming the specificity of this antibody. Furthermore, by immunohistochemistry with another DUSP6 antibody (Origene, TA323084), we found a large number of DUSP6-labeled cells in infarcted LV tissues at 1, 3, and 7 days after MI, but minimal or no DUSP6 expression in the sham-operated hearts (Fig. 3c). We noted that these DUSP6-expressing cells were spherical, ovoid, and distributed within damaged myocardial tissues at 1–3 days after MI and in the infarcted area at 7 days after MI (Fig. 3c). Double immunofluorescence staining showed that the DUSP6 signals co-localized well with the granulocyte surface marker HIS48, myeloperoxidase (MPO), and protease 3 (PR3) in the infarct area at 72 h after MI (Fig. 3d–f). Remarkably, most DUSP6^+^ cells in the infarcted tissue had polymorphic or multi-lobular nuclei, the unique nuclear morphology of neutrophils (Fig. 3d–f). The specificity of this antibody for immunostaining was validated by the absence of DUSP6 signals in the *Dusp6*-deficient infarcted hearts and spleens (Supplementary Fig. 5c).

**Fig. 4:**
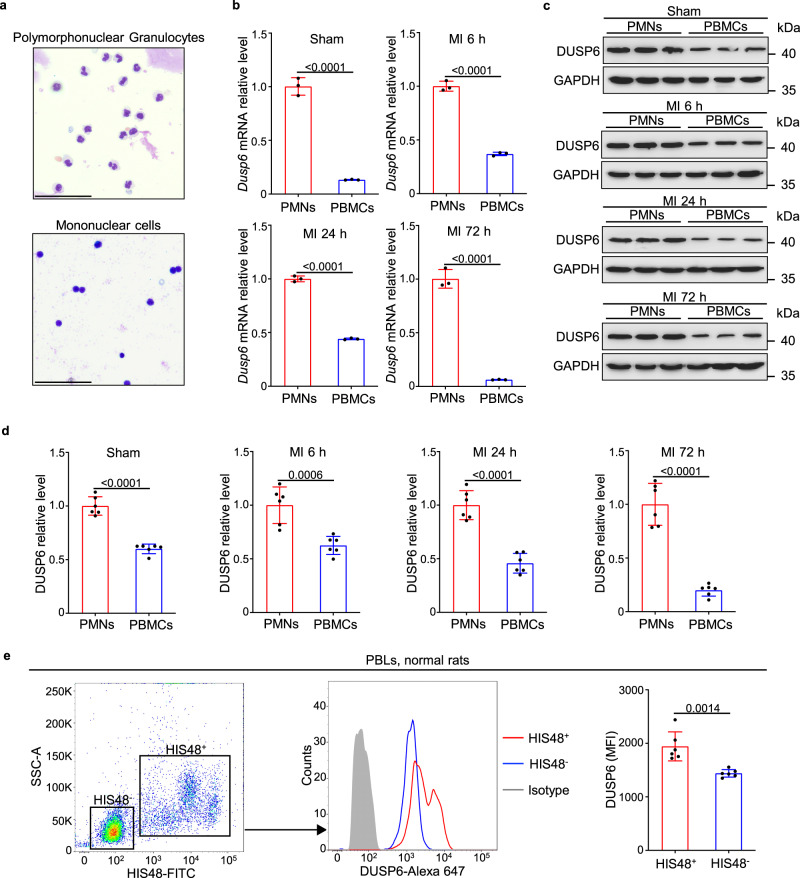
*Dusp6* mRNA and protein are strongly expressed in peripheral neutrophils. **a** Representative Giemsa staining of PMNs and PBMCs separated from PBLs by density-gradient centrifugation and erythrocyte lysis (scale bar, 50 μm). **b** Quantitative RT-PCR of *Dusp6* mRNA in PMNs and PBMCs from WT rats for sham-operated, MI 6 h, MI 24 h, and MI 72 h (performed in duplicates with *n* = 3 biological independent samples/group). **c, d** Western blot and corresponding quantitative analysis of DUSP6 in WT PMNs and PBMCs for sham-operated, MI 6 h, MI 24 h, and MI 72 h (*n* = 6 biological independent samples/group). GAPDH was used as an internal control. All blots within each individual panel were performed in parallel with the same samples. **e** Intracellular staining, flow cytometry and corresponding quantitative analysis of DUSP6 in HIS48^+^ and HIS48^–^ cells from PBLs of unoperated WT rats (*n* = 6 biological independent samples/group). All quantitative data shown in this figure are presented as mean values ± SD. Two-sided unpaired T-test was used to calculate the presented p-values. Source data of **b**–**e** are provided in a Source Data File. PMNs polymorphonuclear cells (peripheral neutrophils), PBMCs peripheral blood mononuclear cells, PBLs peripheral blood leukocytes, MFI, median fluorescence intensity.

In addition, by purifying polymorphonuclear cells (PMNs, mainly neutrophils) and mononuclear cells (PBMCs, including lymphocytes and monocytes) from total peripheral blood leukocytes (PBLs), we found the conspicuously higher levels of *Dusp6* mRNA and protein in PMNs than in PBMCs in the sham-operated controls, and 6, 24, and 72 h after MI, which the purity of these leukocytes was verified by Giemsa staining (Fig. 4a–d). Consistently, flow cytometry analysis with PBLs isolated from normal WT rats also revealed a stronger DUSP6 signal in HIS48^+^ granulocytes (Fig. 4e). Together, these data demonstrate that *Dusp6* is predominantly expressed in neutrophils from both peripheral blood and infarcted LV tissues. Intriguingly, by investigating the RNA levels of *DUSP6* across all human single-cell types utilizing the Human Protein Atlas Database (www.proteinatlas.org)^25^, we found that *DUSP6* expression is most abundant in granulocytes, relatively high level in macrophages, alveolar cells and hepatocytes, while is weak in fibroblasts, smooth muscle cells and cardiomyocytes (Supplementary Fig. 6). Thus, human DUSP6 has expression patterns similar to that of rat DUSP6 (Fig. 3a, b and Supplementary Fig. 1b–i), indicating that *Dusp6*-deficient rats are capable of serving as a reliable animal model for studying human DUSP6 function.

### *Dusp6* deficiency attenuates ROS release and degranulation in neutrophils

We then investigated the impact of *Dusp6* deficiency on neutrophil function. HIS48 antibody staining and flow cytometry revealed comparable ratios of PMNs among total PBLs in WT and *Dusp6*-deficient rats (Supplementary Fig. 7a, b); and *Dusp6* deficiency did not alter the expression of CD11b, a marker of terminal neutrophil differentiation, in PMNs (Supplementary Fig. 7a, c). Infiltrated neutrophils normally peak in the infarct area at 24 h after MI^2^. At this stage, we found that neither the amount of HIS48^+^/CD45^+^ neutrophils nor CD11b level was affected in the *Dusp6*-deficient hearts compared with WT siblings (Supplementary Fig. 7d–f). Together, these data suggest that *Dusp6* deficiency has no effect on neutrophil development, differentiation, or chemotaxis-induced recruitment after MI.

After recruitment to the infarct area in response to MI, activated neutrophils release many cytokines and chemokines including TNF-α and IL-1β, the two main pro-inflammatory factors after MI^26^. ELISA analysis revealed that expression levels of both cytokines were comparable in WT and *Dusp6*-deficient PMNs with or without lipopolysaccharide (LPS) treatment for 18 h (Supplementary Fig. 8a). We also found equal levels of TNF-α and IL-1β in WT and *Dusp6*-deficient CD45^+^/HIS48^+^ infiltrated neutrophils of hearts after MI (Supplementary Fig. 8b, c), suggesting that *Dusp6* deficiency has no effect on the release of pro-inflammatory cytokines from neutrophils in both peripheral blood and infarcted heart tissue.

**Fig. 5:**
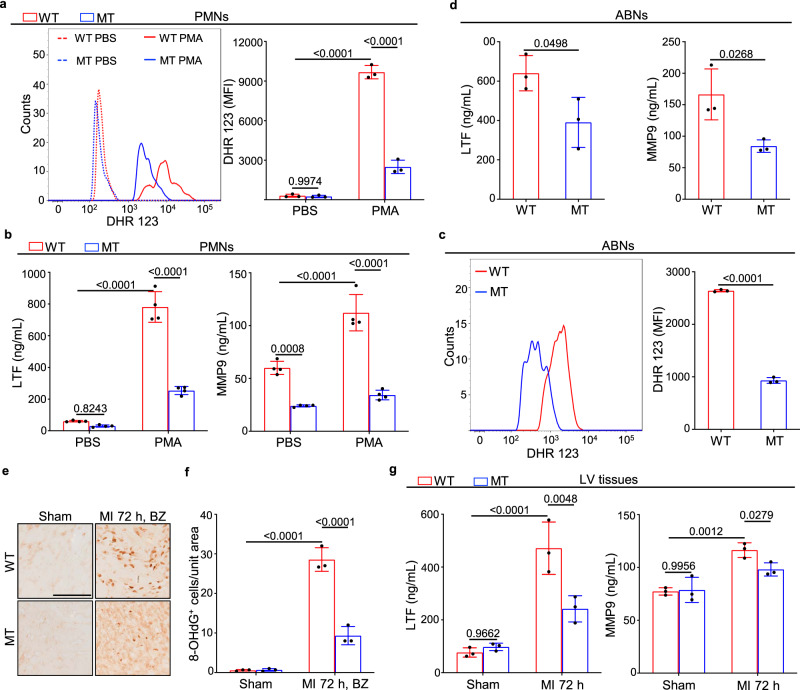
Attenuation of ROS production and degranulation in *Dusp6*-deficient neutrophils. **a** Dihydrorhodamine 123 (DHR 123) staining, flow cytometry and corresponding quantitative analysis of ROS levels in WT and *Dusp6*-deficient PMNs with either PBS or PMA stimulation (*n* = 3 biological independent samples/group). **b** ELISA analysis of LTF and MMP9 release in WT and *Dusp6*-deficient PMNs with either PBS or PMA stimulation (*n* = 4 biological independent samples/group). **c**, **d** DHR 123 staining assays of ROS production (*n* = 3 biological independent samples/group), and ELISA analysis of LTF and MMP9 release (*n* = 3 biological independent samples/group) in WT and *Dusp6*-deficient ABNs. Gating strategies for peripheral and abdominal neutrophils are shown in Supplementary Fig. 4b. **e**, **f** Representative immunohistochemistry images and corresponding quantitative analysis of 8-OHdG staining in WT and *Dusp6*-deficient LV tissue from sham-operated or 72 h after MI (*n* = 9 areas from 3 hearts/group; scale bar, 100 μm; BZ, infarct border zone). **g** ELISA analysis of the levels of LTF and MMP9 in WT and *Dusp6*-deficient LV tissue from sham or 72 h after MI (*n* = 3 biological independent samples/group). All quantitative data shown in this figure are presented as mean values ± SD. One-way ANOVA with Tukey’s multiple comparison test (for **a**, **b**, **f** and **g**) and Two-sided unpaired *T*-test (for **c** and **d**) were used to calculate the presented *p*-values. Source data of **a**–**d**, **f** and **g** are provided in a Source Data File. WT wild-type, MT *Dusp6* mutant, PMA phorbol-12-myristate-13-acetate, DHR 123 dihydrorhodamine 123, LTF lactoferrin, MMP9 matrix metallopeptidase 9, ABNs abdominal neutrophils, 8-OHdG 8-hydroxy-2′-deoxyguanosine.

We then assessed the levels of neutrophil-released granule components and reactive oxygen species (ROS), which largely contribute to cardiac damage and remodeling after MI^27^. As an indispensable effector of neutrophil antimicrobial activity, ROS are released through a process called respiratory burst that directly causes DNA damage and protein modification, leading to cell death and tissue injury. Dihydrorhodamine 123 (DHR 123) staining and flow cytometry analysis showed that the ROS levels were markedly lower in *Dusp6* mutant PMNs than in WT controls with or without phorbol myristate acetate (PMA) stimulation (Fig. 5a). Likewise, *Dusp6* deficiency resulted in decreased ROS levels elicited by glycogen in abdominal neutrophils (ABNs) (Fig. 5c). By performing immunohistochemistry analysis of 8-hydroxy-2′-deoxyguanosine (8-OHdG), a modified base indicator of DNA damage associated with oxidation, we found that oxidative injury caused by neutrophil respiratory bursts decreased in the infarct border zone *of Dusp6*-deficient LV tissues compared with WT controls at 72 h after MI (Fig. 5e, f). Thus, *Dusp6* ablation attenuates neutrophil respiratory bursts and the consequent cardiac tissue damage after MI.

Degranulation is another major function of neutrophils in protecting against infection and non-infectious inflammatory responses. The granule components including azurophilic granule, secondary and tertiary granules, as well as secretory vesicles, are involved in various inflammation-associated diseases including MI^27^. ELISA assays revealed that *Dusp6* deficiency decreased the release of the secondary granule marker lactoferrin (LTF) and the tertiary granule marker matrix metallopeptidase 9 (MMP9) in PMA-stimulated PMNs (Fig. 5b), as well as in glycogen-elicited ABNs (Fig. 5d). The release of MMP9 was also reduced in *Dusp6*-deficient PMNs even without PMA stimulation, probably due to stimulatory effects during the neutrophil separation processing. ELISA assays of LV tissue extracts showed elevated levels of the granule markers LTF and MMP9 after MI, but both markers were attenuated in *Dusp6* mutants (Fig. 5g). In contrast, the levels of MPO and neutrophil elastase (NE), markers of azurophilic granules and neutrophil extracellular traps (NETs), were comparable in extracts of WT and *Dusp6*-deficient hearts (Supplementary Fig. 8d). Taken together, these data demonstrate that *Dusp6* deficiency attenuates neutrophil respiratory burst and the release of secondary/tertiary granules in PMNs, ABNs, and LV tissues.

### *Dusp6* deficiency in neutrophils decreases cardiac damage in vitro and in vivo

We then investigated whether DUSP6 ablation in neutrophils is sufficient to alleviate post-MI cardiac damage. We first designed an in vitro myocardial injury model by co-cultivating primary NRVMs with glycogen-elicited ABNs endogenously activated and isolated from WT and *Dusp6*-deficient rats (Supplementary Fig. 9a). The numbers of adherent NRVMs were markedly reduced after 12 h of co-culture with WT ABNs compared with NRVMs alone, reflecting the damage of activated neutrophils to primary NRVMs. Interestingly, NRVMs co-cultured with *Dusp6* mutant ABNs showed better viability (Supplementary Fig. 9b, c) compared with those co-cultured with WT ABNs. These data directly demonstrate that *Dusp6*-deficient neutrophils are less lethal for NRVMs, consistent with their reduced ROS production and degranulation.

To fulfill the ablation of DUSP6 in neutrophils in vivo, we performed bone marrow transplantation. Adult WT rats were irradiated with Co^60^ to thoroughly destroy the intrinsic hematopoietic system, and then given an intravenous injection of WT or *Dusp6*-deficient nucleated bone marrow cells (Supplementary Fig. 11a). Giemsa staining of blood smears revealed that the peripheral leukocytes disappeared 5 days after radiation and transplantation (Supplementary Fig. 10d–f) compared with smears before irradiation (Supplementary Fig. 10a–c), and re-emerged at 9 days after transplantation (Supplementary Fig. 10g–i). Moreover, Western blot analysis showed a complete loss of DUSP6 protein in ABNs from WT rats transplanted with *Dusp6*-deficient bone marrow cells, demonstrating the successful ablation of DUSP6-expressing neutrophils in recipient WT rats (Supplementary Fig. 11b). Similar to the results from global *Dusp6* mutant rats, transplantation of *Dusp6*-deficient bone marrow cells led to attenuated ROS production and degranulation (LTF and MMP9) in ABNs (Supplementary Fig. 11c, d). More interestingly, *Dusp6*-deficient BMC transplantation was sufficient to decrease the infarct area at 72 h after MI (Supplementary Fig. 11e, f), while transplantation of WT BMCs to *Dusp6*-deficient rats abrogated the reduction of infarct area at the same stage (Supplementary Fig. 11g, h).

**Fig. 6:**
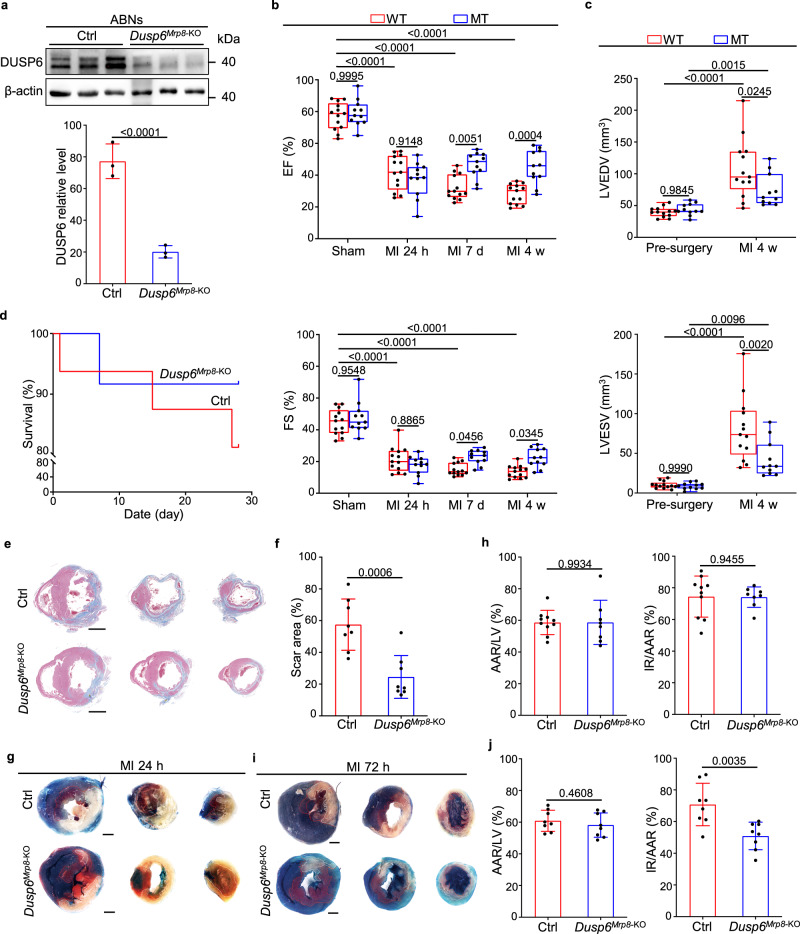
Neutrophil-specific *Dusp6* ablation attenuates post-MI cardiac damage. **a** Western blot and corresponding quantitative analysis of Dusp6 in ABNs from control and *Dusp6*^*Mrp8*-KO^ mice. Control mice were *Mrp8*-Cre, and *Dusp6*^*Mrp8*-KO^ mice were *Mrp8*-Cre, *Dusp6*^f/f^. β-actin served as internal control (*n* = 6 biological independent samples/group). Both blots were performed in parallel with the same samples. **b** Echocardiographic measurements of EF and FS for control *(n* = 13) and *Dusp6*^*Mrp8*-KO^ (*n* = 11) mice before surgery, as well as 24 h, 7 days and 4 weeks after MI. **c** Echocardiographic measurements of LVEDV and LVESV for control (*n* = 13) and *Dusp6*^*Mrp8*-KO^ (*n* = 11) mice before surgery and 4 weeks after MI. The box blots show center lines as median, box boundaries as upper and lower quartiles, and whiskers as minimum and maximum values. **d** Kaplan–Meier survival curves of control (*n* = 16) and *Dusp6*^*Mrp8*-KO^ mice (*n* = 12) at 4w after MI. Representative Masson staining of heart sections (**e**) and quantitative analysis (**f**) of the fibrotic area from control and *Dusp6*^*Mrp8*-KO^ hearts at 4 weeks after MI (*n* = 8/group. Scale bar, 1 mm). Representative TTC-Evans blue staining (**g** and **i**) and measurements of infarct size in control and *Dusp6*^*Mrp8*-KO^ hearts at 24 h (**h**) (control: *n* = 10; *Dusp6*^*Mrp8*-KO^: *n* = 8) and 72 h (**j**) (*n* = 8/group) after MI. Scale bar, 1 mm. Quantitative data are presented as min to max with all points mean in **b** and **c**, and as values ± SD in **a**, **f**, **h** and **j**. One-way ANOVA with Tukey’s multiple comparison test (for **b** and **c**) and Two-sided unpaired *T*-test (for **a, f, h** and **j**) were used to calculate the presented p-values. Source data of **a**–**d**, **f, h** and **j** are provided in a Source Data File. Ctrl control, EF ejection fraction, FS fractional shortening, LVEDV left ventricular end-diastolic volume, LVESV left ventricular end-systolic volume, TTC triphenyl tetrazolium chloride, IR infarcted region, AAR area at risk, LV left ventricle.

To more specifically investigate the role of neutrophil DUSP6 in improvement of cardiac function post-MI, we generated and executed two other animal models. First, we generated neutrophil-depleted *Dusp6*-deficient and WT rats by injecting polyclonal rabbit anti-rat PMN antibody for three consecutive days as previously reported^28^, which were then subjected to MI. Notably, upon the depletion of circulating neutrophils (Supplementary Fig. 12a, b), both *Dusp6*-deficient and WT rats exhibited comparable levels of LV function and morphology, as represented by ejection fraction and fractional shortening at different time points (Supplementary Fig. 12c) as well as LV diastolic and systolic volumes at 4 weeks after MI (Supplementary Fig. 12d). Furthermore, to specifically ablate *Dusp6* in neutrophils, we generated *Dusp6*-floxed mouse line using CRISPR/Cas9 technology (Supplementary Fig. 13), and subsequently crossed it with *Mrp8*-Cre transgenic mice, which express Cre recombinase specifically in neutrophils^29,30^, to establish a neutrophil-specific *Dusp6* knockout mice (*Dusp6*^*Mrp8*-KO^). The protein level of DUSP6 was markedly decreased in *Dusp6*^*Mrp8*-KO^ neutrophils compared with that of *Mrp8*-Cre controls (Fig. 6a). Strikingly, although the LV function and volume indexes were comparable between *Dusp6*^*Mrp8*-KO^ and control mice before and 24 h after MI, we found that ejection fraction and fractional shortening increased in *Dusp6*^*Mrp8*-KO^ hearts at 7 d and 4 weeks after MI (Fig. 6b), as well as LV diastolic and systolic volumes decreased at 4 weeks after MI (Fig. 6c). Consistent with the echocardiographic results, *Dusp6*^*Mrp8*-KO^ mice displayed slightly higher survival rate, as well as reduced scar area in LV by 4 weeks after MI (Fig. 6d–f). Moreover, by performing TTC-Evans blue staining, we observed a reduction of infarct size in *Dusp6*^*Mrp8*-KO^ hearts at 72 h, rather than 24 h, after MI (Fig. 6g, h), whereas the AAR between *Dusp6*^*Mrp8*-KO^ and control hearts was comparable at both stages (Fig. 6i, j), indicating that neutrophil-specific ablation of *Dusp6* contributes to the reduction of progressive cardiac damage, rather than acute ischemic cardiac injury, in *Dusp6*^*Mrp8*-KO^ infarcted hearts. Taken together, these results demonstrate a predominant and common role of neutrophil *Dusp6* in regulating post-MI cardiac injury and remodeling in both rats and mice.

### DUSP6 plays a key role in reciprocal inhibition between ERK and p38 in resting neutrophils

**Fig. 7:**
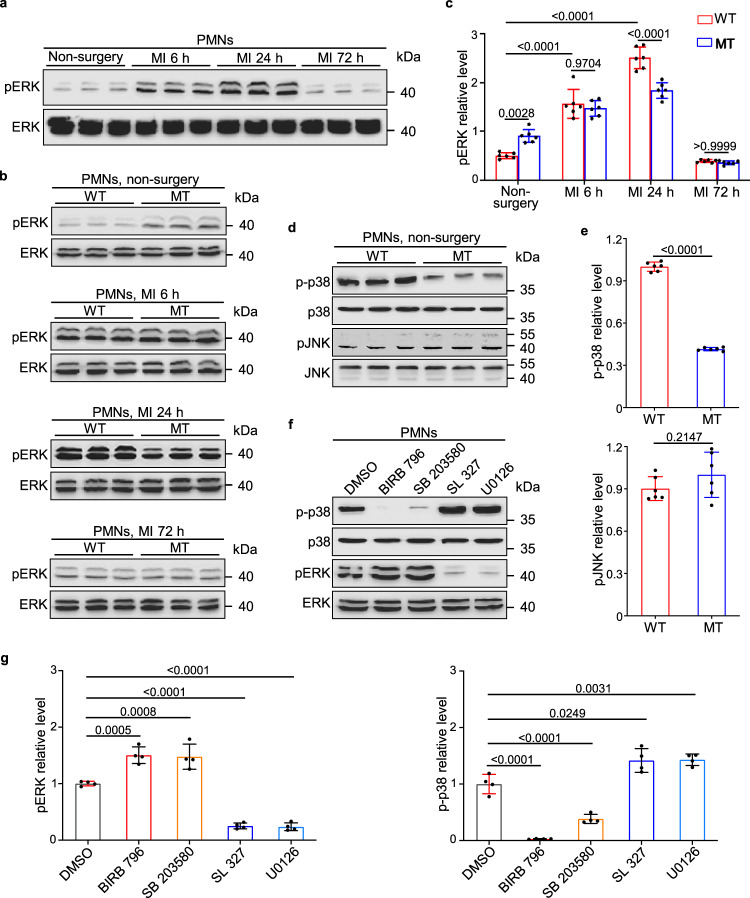
*Dusp6* deficiency interferes with the cross-talk between basal pERK and p-p38 in resting peripheral neutrophils. **a** Western blot of pERK in WT PMNs in unoperated controls and 6–72 h after MI (*n* = 6/group). Both blots were performed in parallel with the same samples. **b**, **c** Western blot and quantitative analysis of pERK levels in WT and *Dusp6*-deficient PMNs in unoperated controls and 6–72 h after MI (*n* = 6/group). **d**, **e** Western blot and quantitative analysis of p-p38 and pJNK in PMNs from unoperated WT and *Dusp6*-deficient rats (*n* = 6/group). **f**, **g** Western blot and quantitative analysis of pERK and p-p38 levels in unoperated WT PMNs treated with either p38 inhibitors (BIRB796 and SB203580) or MEK1/2 inhibitors (SL327 and U0126). DMSO was used as a negative control (*n* = 4 independent experiments). All blots within each individual panel were performed in parallel with the same samples. All quantitative data shown in this figure are presented as mean values ± SD. One-way ANOVA with Tukey’s multiple comparison test (for **c** and **g**) and Two-sided unpaired *T*-test (for **e**) were used to calculate the presented *p*-values. Source data of **a**–**g** are provided in a Source Data File. WT wild-type, MT *Dusp6* mutant.

We then asked how DUSP6 engages in neutrophil activity. Since DUSP6 is a well-known phosphatase specifically against ERK1/2 phosphorylation^11^, we first assessed the pERK level in isolated PMNs by Western blot, and found that pERK in PMNs increased from 6 to 24 h, and then decreased to the basal level at 72 h after MI compared with the non-surgery group (Fig. 7a). By comparative analysis of the pERK levels in WT and *Dusp6*-deficient PMNs, we found that basal pERK increased in resting PMNs from *Dusp6*-deficient hearts without MI surgery, but the level of pERK in PMNs in *Dusp6*-deficient heart at 6 h, 24 h, and 72 h after MI did not increase and rather slightly decreased at 24 h after MI compared with those in WT hearts (Fig. 7b, c). This finding is consistent with a previous study showing that knockout of mouse *Dusp6* augments basal ERK phosphorylation instead of stimulus-induced ERK phosphorylation^18^. Regarding the phosphorylation levels of the other two MAPKs p38 and JNK, we found that p38 MAPK phosphorylation (p-p38) was decreased while the phosphorylation levels of JNK1/2/3 (pJNK) were unaffected in *Dusp6* mutant neutrophils (Fig. 7d, e). These data suggest that DUSP6 may be involved in reciprocal inhibition between ERK1/2 and p38 MAPK signaling in resting PMNs.

To address this reciprocal inhibition hypothesis, we isolated resting PMNs from normal WT rats and treated them with dimethyl sulfoxide (DMSO), p38 inhibitors (BIRB796 or SB203580), or MEK1/2 inhibitors (SL327 or U0126). Western blot analysis revealed that the treatment of p38 MAPK inhibitors increased pERK level, while suppression of pERK by inhibitors of its upstream kinase MEK1/2 resulted in a reciprocal increase of p-p38. In particular, treatment of PMNs with SB203580, which inhibits the kinase activity of p-p38 without interfering with its phosphorylation, also caused a downregulation of the p-p38 level accompanied by enhanced pERK, further suggesting the inhibitory effect of pERK on p38 phosphorylation (Fig. 7f, g). Taken together, these data suggest that DUSP6 negatively regulates basal pERK to maintain a relatively high level of p-p38 in normal PMNs.

As noted above, we found that the release of ROS and secondary/tertiary granules were attenuated (Fig. 5), which are dependent on p38 MAPK activity^31^, but the release of p38-independent azurophilic granules and neutrophil extracellular traps^31^ were not affected in *Dusp6* mutant neutrophils (Supplementary Fig. 7d). Moreover, *Dusp6* deficiency only elevated the basal pERK level in resting but not activated neutrophils (Fig. 7b, c). Thus, we speculated that the attenuated p38 MAPK signaling in *Dusp6*-deficient neutrophils prior to activation is sufficient to interfere with their ROS production and granule release after stimulation. To test this hypothesis, we designed ‘pre-modulation’ protocols by pre-treating PMNs with either p38 or MEK1/2 inhibitors (Supplementary Fig. 14a, b). Interestingly, before PMA stimulation, treatment of PMNs with p38 inhibitors (BIRB796 or SB203580), but not MEK1/2 inhibitors (SL327 or U0126), decreased the ROS production and granule release (Supplementary Fig. 14c, d). These data further demonstrate that the attenuated respiratory burst and degranulation in rat *Dusp6*-deficient neutrophils are due to the downregulation of p-p38 rather than pERK in the resting state.

**Fig. 8:**
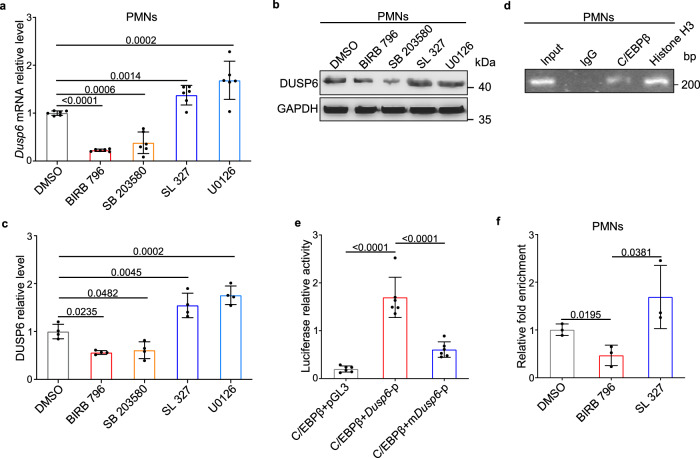
*Dusp6* is transcriptionally regulated by the p38-C/EBPβ pathway in resting PMNs. **a-c** Quantitative RT-PCR (**a** *n* = 6 biological independent samples/group), western blot (**b**) and corresponding quantitative analysis (**c** *n* = 4 biological independent samples/group) of *Dusp6* mRNA and protein in PMNs isolated from unoperated WT rats and treated with either DMSO, p38 inhibitors (BIRB796 or SB203580), or MEK inhibitors (SL327 or U0126). Both blots were performed in parallel with the same samples. **d** Representative ChIP assays reveal the interaction between C/EBPβ and the *Dusp6* promoter fragment (performed in duplicates using PMNs from 5 normal WT rats each time). Anti-Histone H3 and rabbit IgG were used as positive and negative controls, respectively. **e** Luciferase reporter activity driven by the *Dusp6* promoter with WT sequence (*Dusp6*-p) or mutations of putative C/EBPβ-binding motifs (m*Dusp6*-p) upon the regulation of C/EBPβ in 293 T cells (performed in duplicates with *n* = 3/group). The pGL3 empty vector was used as negative control. **f** Quantitative ChIP assays of C/EBPβ binding to the *Dusp6* promoter in WT PMNs treated with either DMSO, p38 inhibitor (BIRB796), or MEK1/2 inhibitor (SL327). 3 replicates were performed with PMNs from at least 5 normal WT rats for each group. All quantitative data shown in this figure are presented as mean values ± SD. One-way ANOVA with Tukey’s multiple comparison test were used to calculate the presented *p*-values. Source data of **a**–**f** are provided in a Source Data File.

Previous studies have reported that *Dusp6* is transcriptionally driven by pERK signaling via the transcriptional factors ETS1 and ETS2, and thus acts as a negative-feedback regulator of ERK signaling^12,32^. Unexpectedly, both *Dusp6* mRNA and protein increased after the inhibition of ERK signaling by MEK1/2 inhibitors but decreased by the treatment with p38 inhibitors (Fig. 8a–c), suggesting that *Dusp6* expression is dependent on p38 activity rather than ERK signaling in resting neutrophils. With a Comparative genome analysis of non-coding DNA sequences within the 5′-flanking regions of *Dusp6* genes in rat, rhesus, and human utilizing Dcode tools (www.dcode.org)^33^, we identified a highly-conserved sequence spanning 746 bp (–2030 to –1284) upstream of the transcriptional start site (Supplementary Fig. 15a). As exhibited by a computational prediction of transcriptional binding sites from PROMO^34^, this putative *Dusp6* enhancer/promoter region contained a number of predicted binding sites for three transcriptional factors, glucocorticoid receptor (GR), CCAAT-enhancer-binding protein alpha (C/EBPα), and C/EBPβ (Supplementary Fig. 15b). Among these, the C/EBPβ binding site was of particular interest because of the high copy of its binding site (Supplementary Fig. 15b) and the known effects of p38 signaling on the induction of C/EBPβ expression and DNA-binding activity^35,36^. To confirm our in silico findings, we performed chromatin immunoprecipitation (ChIP) assays and found that C/EBPβ indeed bound with the putative *Dusp6* promoter sequence (Fig. 8d). Luciferase reporter assay showed markedly enhanced the activity of *Dusp6* promoter, but not the mutant promoter without putative C/EBPβ binding sites, by overexpression of rat C/EBPβ, further validating the regulation of the *Dusp6* promoter by C/EBPβ (Fig. 8e). Furthermore, a quantitative ChIP assay revealed that the binding of C/EBPβ to the *Dusp6* promoter was impaired by p38 inhibitor but enhanced by MEK1/2 inhibitor (Fig. 8f), confirming that *Dusp6* is transcriptionally regulated by the p38-C/EBPβ pathway in resting neutrophils.

DUSP6 plays a vital role in a dynamic network of phosphatase interactions^37^. Thus, we hypothesized that the expression of other phosphatases, especially those p38-targeting DUSP members in neutrophils, are under the control of DUSP6-pERK. By examining the expression levels of all typical *Dusp* genes (except for *Dusp6*) in WT and *Dusp6*-deficient resting PMNs, we found that, among all the annotated *Dusp* genes examined, both *Dusp1* and *Dusp16* mRNAs and proteins, which encode two p38-targeting DUSPs^38,39^, were strongly induced in *Dusp6*-deficient PMNs (Supplementary Fig. 16a, b). Furthermore, the expression of DUSP1 and DUSP16 was blocked by treatment with ERK inhibitors, but was activated by p38 inhibitors (Supplementary Fig. 16c). These results suggest that, in *Dusp6*-deficient neutrophils, DUSP1 and DUSP16 are induced by over-activated ERK signaling due to a lack of DUSP6, which in turn, attenuates p38 activity.

### *Dusp6* deficiency has minimal effect on other immune cell populations and immune homeostasis

We also found *Dusp6* mRNA and protein expression in macrophages and lymphocytes (Figs. 3, 4), which are known to be involved in post-MI cardiac repair. It has been reported that enhanced phagocytosis of dead CMs by macrophages also accelerates the resolution of inflammation and cardiac repair after MI^40^. To assess whether *Dusp6* deficiency leads to more macrophage phagocytosis, we chose to examine the level of Desmin, a subunit of intermediate filaments in CMs, in infiltrated macrophages (gated by CD45^+^ and HIS36^+^) at 72 h after MI using flow cytometry, and found comparable levels of Desmin in WT and *Dusp6*-deficient macrophages, suggesting that *Dusp6* deficiency has no effect on macrophage phagocytosis (Supplementary Fig. 17a, b). Likewise, *Dusp6* deficiency did not alter the expression of pro-inflammatory and reparative factors, TNF-α, IL-1β and TGF-β in infiltrated macrophages of the LV post-MI^41^ (Supplementary Fig. 17c, d). To investigate the global effect of DUSP6 on macrophages in acute inflammatory phase of MI, we performed RNA-seq analysis of CD45^+^HIS36^+^ macrophages isolated from *Dusp6*-deficient and WT infarcted LV tissues at 72 h after MI by fluorescent activated cell sorting (FACS) (Supplementary Fig. 4). Volcano plot analysis identified 638 up-regulated genes and 674 downregulated genes with high significance (log2 fold change >1, adjusted *P* value <0.05) in *Dusp6*-deficient macrophages (Supplementary Fig. 18a). Moreover, gene ontology analysis revealed the profound enrichment of stimulus- and immune-process associated components in the down-regulated gene subsets of *Dusp6*-deficient macrophages (Supplementary Fig. 18b), suggesting a compromised immune response of macrophages of mutant LVs at 72 h after MI, probably owing to the reduced CM cell death in *Dusp6*-deficient rats at this stage (Fig. 2). However, we did not find the holistic alteration on the expression levels of canonical M1 and M2 macrophage markers (Supplementary Fig. 18c), suggesting that DUSP6 has little or no effect on macrophage polarization. Interestingly, a number of ERK and p38 downstream genes were synchronously down-regulated in *Dusp6*-deficient macrophages (Supplementary Fig. 18d), which warrants future investigations.

Previous studies have reported the role of DUSP6 in T-cell differentiation and sensitivity^15,42^. By flow cytometry analysis of various T-cell lineages in the heart-draining mediastinal lymph nodes (MLNs) at 72 h after MI, we found that the ratios of T helper cells (CD4^+^CD8^-^), cytotoxic T cells (CD8^+^CD4^-^), and regulatory T cells (Tregs, CD4^+^CD25^+^FOXP3^+^), a T-cell subset that improves cardiac repair post-MI^43^, were comparable between WT and *Dusp6*-deficient hearts (Supplementary Fig. 19a–d). In addition, as previously reported, these T-cell lineages were minorly detected in infarcted cardiac tissues at 72 h after MI^43,44^. These data suggest that T-cell *Dusp6* has little or no contribution to cardiac repair at the acute inflammatory stage post-MI.

To systematically examine whether *Dusp6* ablation has an impact on rat immune homeostasis, we performed flow cytometry analysis to measure the proportions of multiple immune cell populations, including neutrophils, monocytes, total T and B lymphocytes, CD4^+^ and CD8^+^ T cells, NK cells, and dendritic cells in PBLs and MLNs. By using the previously reported gating strategies^45^ shown in Supplementary Fig. 20a, we found comparable ratios of neutrophils, total T and B lymphocytes, CD4^+^ and CD8^+^ T cells, NK cells, and dendritic cells in WT and *Dusp6*-deficient PBLs, but slightly reduced monocyte ratio in *Dusp6*-deficient PBLs (Supplementary Fig. 20b). As for MLNs, the ratios of total T and B lymphocytes, as well as CD4^+^ T cells were not affected by *Dusp6* deficiency (Supplementary Fig. 20b), although the ratio of CD8^+^ T cells, which was also hardly detected in infarcted cardiac tissues at acute inflammatory phase^44,46^, were slightly reduced. In addition, neutrophils, monocytes, NK cells, and dendritic cells were almost undetectable in MLNs (Supplementary Fig. 20b). These results suggest that *Dusp6* deficiency has minimal effect on the immune homeostasis under physiological condition, especially for the neutrophil and monocyte/macrophage participated innate immunity which predominantly mediates post-MI inflammatory response.

Taken together, our data demonstrate that *Dusp6* is transcriptionally regulated by p38-C/EBPβ signaling in resting neutrophils, and contributes to the restriction of pERK and the p38-targeting phosphatases DUSP1 and DUSP16 at relatively low level. *Dusp6* deficiency in neutrophils upregulates pERK and DUSP1/DUSP16, thus attenuating p-p38, neutrophil respiratory burst and degranulation, and finally resulting in reduced neutrophil-mediated cardiac damage after MI while Dusp6 has minimal effect on other immune cell subsets in post-MI cardiac remodeling (Supplementary Fig. 21).

## Discussion

It is now well-recognized that innate immunity plays a critical role in the pathology of MI. Acute ischemia and the subsequent myocardial necrosis trigger a temporally-defined inflammatory response and the infiltration of leukocytes, mainly neutrophils and monocytes/macrophages^3,47^. Over the years, a great deal of research on the pathogenesis of MI has focused on monocytes/macrophages, partly due to the availability of in vitro culture and infection/transfection as well as of in vivo transgenic and the genetic labeling and/or ablation of macrophages. Several distinct subsets of tissue-resident macrophages have been identified in the human heart at steady-state and during inflammation^48,49^, and they are critical for electrical conduction by interacting with the distal atrioventricular node in the normal heart^50^. Ischemia leads to massive death of cardiac-resident macrophages, accompanied by the recruitment of inflammatory monocytes during the first 3 days and then their transition to reparative phenotypes over the next few days. Although both macrophages are phagocytic, the early inflammatory subset is particularly rich in pro-inflammatory mediators, while the later reparative monocytes support angiogenesis and extracellular matrix synthesis by providing TGF-β and vascular endothelial growth factor (VEGF)^47^. The manipulation of inflammatory macrophages towards the reparative set, or enhancement of phagocytosis, improves cardiac repair by expediting the resolution of inflammation^40,45^. Our data showed that despite a detectable but low-level expression of DUSP6 in HIS36^+^ macrophages within the infarct LV tissue, the phagocytic activity, expression levels of pro-inflammatory factors (TNF-α, IL-1β) and reparative factor TGF-β, and the M1/M2 polarization of *Dusp6*-deficient macrophages were similar to WT controls, suggesting that *Dusp6* deficiency has minimal or no effect on the phagocytic function and immune response of macrophages.

Neutrophils, the other major cell type of innate immunity, account for 35–75% of circulating leukocytes under normal condition. Circulating PMNs are mature, terminally-differentiated cells that have lost their proliferative capacity and have a short circulating lifespan of 5–10 h^51^. Upon MI, they are primed by neutrophil-attracting chemokines and recruited as the first inflammatory cells from the circulation to the infarct area, via an extravasation process consisting of several sequential ligand-receptor interactions^52^. Neutrophil infiltration occurs within several hours, peaks at 1–3 days, and starts to decline from 5 days after MI^2^. Most previous studies on neutrophil function have concentrated on either their depletion or interference with their migration and infiltration. Some investigators have claimed that neutrophil depletion reduces infarct size and the extent of injury in a canine model^53^, but others have found a detrimental effect of neutrophil depletion and a beneficial role of neutrophils in tissue reorganization and wound healing, in which NGAL (neutrophil gelatinase-associated lipocalin) secreted by neutrophils promotes the macrophage-mediated clearance of cell debris^54^. Our data have shown that *Dusp6* deficiency improves post-MI cardiac function by decreasing progressive cardiac cell death (72 h after MI) while having no effect on the early necrosis and apoptosis induced by ischemia (6–24 h after MI). In line with the predominant abundance of *DUSP6* in human granulocytes, DUSP6 was highly enriched in neutrophils from rat peripheral blood, the abdominal cavity, and infarcted LV tissue; and DUSP6 deficiency had no impact on neutrophil development, maturation, and tissue infiltration in LV infarct tissue in the post-MI phase. Thus, our data suggest that specifically attenuating neutrophil function in acute inflammatory stage, but not in the early ischemic phase, contributes to overall cardiac outcome improvement in *Dusp6* mutants.

Neutrophil function in protecting against pathogen infection and non-infectious inflammatory processes depends on the release of ROS, granule components, cytokines, and chemokines, as well as the formation of NETs after apoptosis^27^. Here, we showed that *Dusp6* deficiency dampened the release of ROS and gelatinase granules (MMP9 and LTF) in peripheral and abdominal neutrophils without affecting azurophilic granule release, TNF-α and IL-1β secretion, and NETs formation. The generation of ROS is one of the most efficient antimicrobial mechanisms, but activated neutrophils also damage host tissues and cells by modifying amino acids, proteins, and lipids to alter their biological functions^55^. Members of the MMP family and other proteolytic enzymes decrease the collagen deposition and degrade extracellular matrix, leading to instability of myocardial structures and increasing the risk of cardiac dilatation and rupture^56^. Because of the reduction of oxidative DNA damage, cell death, and the decreased expression of granule markers (LTF and MMP9) of infiltrated neutrophils in *Dusp6*-deficient rat cardiac tissues at 3 days after MI (an important time window with reference to neutrophil action during MI pathogenesis), as well as the results from in vitro co-culture of neutrophils and neonatal rat ventricular cardiomyocytes, rat BMC transplantation and mouse neutrophil-specific *Dusp6* knockout mouse experiments, we conclude that *Dusp6* deficiency dampens ROS release and degranulation from neutrophils at the acute inflammatory phase post-MI and thus attenuates neutrophil-induced cardiac damage without affecting the process of neutrophil differentiation or chemotaxis.

Previous studies have shown reciprocal inhibition between pERK and p38 MAPK signaling in neutrophils and other cell types^38,57–59^. DUSP1, a nuclear phosphatase that is normally induced by ERK activation, contributes to the inhibition of p-p38 via dephosphorylation^60,61^. However, the reciprocal inhibition between pERK and p38 remained incompletely understood. Our data have shown that DUSP6 functions as an effector of p38 signaling and participates in the reciprocal inhibition between pERK and p38. Different from the previous findings that pERK signaling regulates *Dusp6* gene expression via ETS (E26 transformation-specific) transcription factors^32^, we demonstrated that *Dusp6* is under the control of the p38-dependent transcription factor C/EBPβ, which is enriched during the maturation of neutrophils^62^ and is indispensable for the maintenance of neutrophil survival and function^63^. Both DUSP1 and DUSP16, another p38-targeting phosphatase^39^, were upregulated in *Dusp6*-deficient and p38 inhibitor-treated PMNs, while they were down-regulated in PMNs treated with MEK1/2 inhibitors. Upregulation of these two phosphatases downstream of ERK in *Dusp6*-deficient PMNs blocked p38 activity. Notably, it is well known that both DUSP1 and DUSP16 also execute robust dephosphorylation of pJNK^64–66^, although the basal JNK activity was not affected in case of enhanced DUSP1/16 levels in *Dusp6*-deficient PMNs, suggesting that basal JNK activity is under the control of other unknown signaling pathways. Nonetheless, our mechanistic findings suggest that *Dusp6* is transcriptionally regulated by p38-C/EBPβ, and negatively regulated the level of pERK, thus leading to downregulation of DUSP1/16 and up-regulation of p-p38. Since our data are not sufficient to fully address the regulatory network of MAPK phosphatases and their target kinase cascades, it warrants future investigations how this reciprocal inhibition of ERK and p38 is regulated in neutrophils and other cells.

ERK and p38 signaling play opposite roles in regulating neutrophil activity^59,67^. Especially the release of ROS, secondary and gelatinase granules (LTF and MMP9) from neutrophils, which are stimulated by bacteria-derived chemotactic factors and pro-inflammatory cytokines, can be abated by the blockade of p38 rather than ERK activity^31,68–70^. Consistently, our findings here support a critical role of p38 signaling in regulating neutrophil activity; that is, DUSP6 plays a crucial role in the maintenance of p38 activity and the consequent neutrophil killing activity by reducing the pERK level. Notably, the reciprocal inhibition between ERK and p38, the efficacy of DUSP6 in ERK dephosphorylation, and the transcriptional regulation of *Dusp6* expression by p38-C/EBPβ only take place in resting PMNs, but not in any type of activated neutrophils. Consistent with previous reports^67,68^, our data further substantiate that modulation of ERK/p38 in neutrophils before stimulation is sufficient to control ROS release and degranulation. In addition, the latent role of JNK pathway in *Dusp6*-deficient PMNs still warrants future investigation, as our data cannot exclude the involvement of JNK activity, which is also functional in neutrophil survival, chemotaxis and killing of pathogens^71^.

In conclusion, by utilizing robust mouse and rat models that resembled DUSP6 expression patterns in humans, we have identified DUSP6 as an indispensable factor for regulating the tissue-damaging activity of neutrophils at the acute inflammatory phase post-MI. In resting neutrophils, *Dusp6* deficiency leads to unrestricted ERK phosphorylation and up-regulation of the p38 phosphatases DUSP1 and DUSP16, thus decreasing p38 activity and subsequently dampening the release of ROS and granule components after a stimulus, and finally alleviating neutrophil-mediated cell death and tissue damage during the inflammatory stage of MI (Supplementary Fig. 21). Our findings explicitly highlight that *Dusp6* deficiency attenuates the tissue injury after MI rather than the ischemic necrosis and reperfusion injury, thus improving the outcome of post-MI cardiac repair by suppressing neutrophil cytotoxicity while having minimal effects on other immune cells. This raises the novel possibility of attenuating DUSP6-dependent neutrophil activity after MI while having no effect on early neutrophil development, differentiation and infiltration for human cardiovascular diseases in particular and other neutrophil-related diseases in general.

## Methods

### Animals

*Dusp6* mutant Sprague-Dawley rats were generated using CRISPR/gRNA technology as previously reported^19^. To execute neutrophil depletion, rats were administrated intraperitoneal injection of rabbit polyclonal antibody against rat neutrophils (anti-PMN; Accurate Chemical Co, AIAD51140) at a dose of 250 μL/kg for three consecutive days.

To generate *Dusp6*-floxed mouse line, mouse *Dusp6* genomic DNA was amplified by PCR using the following primer pairs:

The upstream homologous arm: 5′-GATCCCGTGACCTTAGACGC-3′ and 5′-GGGAAAGCGACACAGAAGTCA-3′;

The downstream homologous arm: 5′-TGGTTACTGTGAGCCTCTGG-3′ and 5′-CCAAATGGGCGCTCATACTC-3′;

target sequence: 5′-TCTGTGTCGCTTTCCCTAACC-3′ and 5′-GTGCAGCTAACAATGGGGGT-3′.

PCR products were subcloned into a vector containing loxP sites and served as donor plasmid for CRISPR/Cas9-mediated homologous recombination. Single guide RNAs were designed using online software from Feng Zhang’s lab (http://crispr.mit.edu/) as the following sequences:

gRNA-up (for upstream loxP site): CAAGCCGAGGCTAGCGGTTAGGG;

gRNA-down (for downstream loxP site): TAACCTGATCCCCCGCAGGA AGG.

Mouse embryonic stem cells (1 × 10^7^) were electroporated with a combination of Cas9 plasmids (8 μg), 2 gRNAs (4 μg each), and donor plasmid (8 μg) in 800 μl ice-cold DPBS (Thermo Fisher, 14190144) using the Gene Pulser Xcell System (Bio-Rad) at 250 V and 500 μF in 4 mm cuvettes (Bio-Rad). Clones were then picked and screened by PCR using the following primers:

Primer pair 1: 5′-TCGCCTTCTATCGCCTTCTT-3′ and 5′-AGAGAGAAAGGGACCCAGGA-3′;

Primer pair 3: 5′-CAAGTCGCCCTGTTTTAGCC-3′ and 5′-TCTGCCAATCACCCCTTAAGT-3′.

Two positive clones were injected into mouse blastocysts to generate chimeras for further identification of *Dusp6*-floxed founder mice, which were bred with C57BL/6 mice to generate germline-transmitted *Dusp6*-floxed mice. To generate *Dusp6* neutrophil-specific knockout mice, homozygous *Dusp6*-floxed (*Dusp6*^f/f^) mice was crossed with *Mrp8*-Cre mice, which were a gift from Dr. Fengxia Ma at the Institute of Hematology and Blood Diseases Hospital, Chinese Academy of Medical Sciences and Peking Union Medical College, Tianjin, China. Wild-type Sprague-Dawley rats and C57BL/6 mice were purchased from Vital River Laboratory Animal Technology Co., Ltd (Beijing, China). Both rats and mice were raised and handled with the animal protocol (IMM-XiongJW-4) approved by the Peking University Institutional Animal Care and Use Committee, which is fully accredited by the Association for Assessment and Accreditation of Laboratory Animal Care International. Animals were maintained at the ambient temperature from 21 to 23 °C and humidity ranges from 40 to 70% with the 12-h light/12-h dark cycle. Male rats and mice at the matched age of 8–10 weeks old were utilized for all analyses.

### Rat and mouse myocardial infarction model

The MI model was created by ligation of the LAD coronary artery in male rats or mice at the matched age of 8–10 weeks as previously described and without reperfusion^22^. Briefly, each animal was anesthetized by intraperitoneal injection of pentobarbital sodium (30 mg/kg). After complete anesthesia, animals were immobilized in the supine position on an operating table, intubated, and connected to a small-animal ventilator (MouseVent, Kent Scientific Corp., Torrington, CT, USA). Under fully-controlled ventilation, a thoracotomy was made in the left intercostal space between the third and fourth ribs, and the heart exposed by removal of the pericardium. The LAD coronary artery was permanently ligated using a 6–0 (for rats) or 7–0 (for mice) non-absorbable surgical suture, and the chest and skin immediately closed. Animals were finally removed from the ventilator and kept warm until completely revived. Animals with sham surgery were subjected to the same procedure without performing the LAD ligation step.

### Echocardiography

Echocardiography was performed with the Vevo 2100 high-resolution ultrasound system (Fujifilm VisualSonics Inc., Toronto, ON, Canada) at two time points: before surgery and 4 weeks after MI. Briefly, each animal was anesthetized with 1% isoflurane and then immobilized supine on a heating pad kept at 37 °C. The hair on the left chest was completely removed using a depilatory paste. A 20 MHz variable frequency transducer was used to capture 2-D echocardiographic images on both the mid-ventricular short axis and the parasternal long axis. The resulting images were analyzed to derive indexes of cardiac function and LV dilation on the basis of a standard formula as previously described^72^.

### Quantification on the numbers of LV cardiomyocytes

Quantification on the LV cardiomyocyte numbers was performed as previously described^73^ with minor modifications. Briefly, fresh LV tissues were minced to small pieces and immediately fixed in 4% PFA overnight. Samples were subsequently digested with 0.4 mg/mL Collagenase Type II (Gibco, 17101015) for 12 h at 37 °C. The supernatant was then collected and spun down (500 rpm, 2 min) to obtain isolated cardiomyocytes. The procedure was repeated until no more cardiomyocytes were dissociated from tissue samples. The total numbers of cardiomyocytes were counted from a 10-μL aliquot of cell suspensions with 150–200 cardiomyocytes/aliquot counted by a hemocytometer.

### Measurement of infarct size by TTC-Evans blue staining

Triphenyl tetrazolium chloride (TTC)-Evans blue staining was performed according to a previous description^22^ with some modifications. Briefly, each rat or mouse was anesthetized by intraperitoneal injection of pentobarbital sodium (30 mg/kg). After exposure, the heart was perfused with 1% Evans blue by intracardiac injection, and then excised. After freezing at –20 °C for 60 min, the heart was cut into 3–4 slices, which were then incubated in PBS containing 1% TTC (37 °C, 15 min) to visualize the unstained infarct region.

### Masson’s trichrome staining

Paraffin sections of WT and mutant hearts were cut and stained with a Masson’s Trichrome Stain Kit (Leagene, DC0034). The myocardium was red and collagen fibers were bright green.

### Immunostaining

Rat heart, spleen, and other tissues were collected and fixed overnight in 4% paraformaldehyde (PFA) at room temperature. For the detection of DUSP6, periodate-lysine-PFA fixative (Leagene, DF0071) was used instead. The tissues were then dehydrated with gradient concentrations of ethanol, cleared in xylene, and finally embedded in paraffin. The tissue blocks were sectioned at 2 μm for subsequent histological studies as previously described^9^. In brief, the paraffin sections were dewaxed in xylene, rehydrated in a series of ethanols, and then washed in water and PBS. For antigen retrieval, the sections were rinsed in pre-heated citric acid buffer (pH 6.0) and boiled in a microwave for 10 min. After washing in water and PBS, the sections were blocked in 10% FBS in PBST for 30 min, and then incubated overnight with primary antibodies. For immunofluorescence staining, the sections were incubated with corresponding fluorescence-labeled secondary antibodies at room temperature for 2 h, and counterstained with DAPI. For histochemical staining with diaminobenzidine (DAB), the sections were processed with the horseradish peroxidase (HRP)/Fab Polymer Conjugated Detection System (ZSGB, PV6001) and DAB Substrates (ZSGB, ZLI9033) according to the manufacturer’s instructions, and counterstained with Mayer’s hematoxylin. The primary antibodies were anti-DUSP6 (1:100, Origene, TA323084), anti-HIS48 (1:50, clone HIS48, Abd Serotec, MCA967), anti-MPO (1:50, clone 8F4, Novus, NBP1-51148), anti-protease 3 (1:50, clone D-1, Santa Cruz, sc-74534), and anti-8-OHdG (1:100, clone N45.1, Abcam, ab48508). The secondary antibodies were Alexa Fluor 488 goat anti-rabbit IgG (1:1000, Thermo Fisher, A32731), Alexa Fluor 555 goat anti-mouse IgG (1:1000, Thermo Fisher, A21422), Alexa Fluor 555 goat anti-mouse IgM (1:1000, Thermo Fisher, A21426), and Alexa Fluor 555 goat anti-rat IgG (1:1000, Thermo Fisher, A21434). Confocal microscopy imaging was performed with Zeiss LSM 510 Meta microscope and Zen 3.1 software.

### TUNEL assay

TUNEL assays were executed on paraffin sections of WT and mutant hearts with an In Situ Cell Death Detection Kit (Roche, 11684817910) that labeled the nuclei of dying cells with green fluorescence. Cardiomyocytes were co-stained by immunostaining with cTnT antibody (1:500, clone 1C11, Abcam, ab8295) and Alexa Fluor 555 goat anti-mouse IgG (1:1000, Thermo Fisher, A21422).

### Isolation of rat peripheral and abdominal neutrophils

To isolate peripheral neutrophils, ~6 mL of whole blood was drawn from the abdominal aorta and collected into EDTA-containing tubes. Peripheral neutrophils were isolated using the Rat Peripheral Blood Neutrophil Isolation Kit (TBD Science, LZS1091) according to the manufacturer’s protocol. Briefly, 5 mL of neutrophil separation solution was placed in a Falcon tube, and the same volume of whole blood was carefully layered to make a two-step gradient. The Falcon tube was centrifuged in a swinging-rotor centrifuge at 400 × *g* for 45 min. At the end of centrifugation, two distinct phases were evident, an upper phase (plasma, mononuclear cells, and separation solution gradient) and a lower phase (neutrophils and erythrocytes). The lower phase was carefully collected and transferred to a new tube, where erythrocytes were lysed with ACK Lysis Buffer (TBD Science, NH4CL2009) and neutrophils were collected by centrifugation and washed twice with Hanks' balanced salt solution (HBSS) (MacGene, CC016).

Glycogen-elicited rat ABNs were prepared using a method described previously^74^ with minor modifications. First, 10 mL of 1% glycogen (dissolved in 0.9% NaCl) was injected intra-peritoneally to accumulate neutrophils for 6 h. The rats were then anesthetized and euthanized by decapitation. Peritoneal cells were harvested by intraperitoneal lavage using RPMI 1640 medium (10 mL per rat). The peritoneal exudate was filtered through gauze, and centrifuged at 500 × *g* for 10 min. After lysis of contaminating erythrocytes with ACK Lysis Buffer, the neutrophils were collected by centrifugation and washed twice with HBSS.

Isolated peripheral and abdominal neutrophils were re-suspended in RPMI 1640 with 1% bovine serum albumin (BSA) for further use. For treatment with chemical inhibitors, neutrophils were incubated with BIRB796 (10 μmol/L, Selleck, S1574), SB203580 (20 μmol/L, Sigma, S8307), SL 327 (10 μmol/L, Sigma, S4069), or U0126 (10 μmol/L, Sigma, U120) for 24 h or 60 min (for the pre-modulation of neutrophil respiratory burst and degranulation) at 37 °C.

### Assays of neutrophil respiratory bursts and degranulation

ROS released by neutrophils were assayed using the fluorescent indicator Dihydrorhodamine 123 (DHR 123) according to the manufacturer’s instructions. In brief, 1 × 10^6^ freshly-isolated peripheral neutrophils were incubated with DHR 123 (5 μg/mL) for 1 h in RPMI 1640 with 1% BSA at 37 °C. To stimulate respiratory bursts, 1 μM PMA (Sigma, P8319) was administered simultaneously. The non-fluorescent dye DHR 123 was converted into the fluorescent compound rhodamine 123 by ROS produced by neutrophils. The fluorescence intensity of the stained neutrophils was analyzed by flow cytometry. The cell-free supernatants from cultured neutrophils were collected for the assessment of neutrophil degranulation using ELISA kits for the neutrophil granule markers lactoferricin (LifeSpan, F4351) and MMP9 (LifeSpan, F5605), according to the manufacturer’s instructions. For assessment with ABNs, the procedures were similar but PMA stimulation was not required. For assessment of endogenous ROS level in PMNs, 100 μL whole blood was collected and incubated with incubated with DHR 123 for 1 h at 37 °C*, and were then subjected to* erythrocyte lysis and flow cytometry analysis.

To assay the lactoferricin and MMP9 levels in the infarct area, heart tissue was homogenized in PBS containing 1 mM phenylmethylsulfonyl fluoride (PMSF) (Sigma, 93482) and protease inhibitor cocktail (Roche, 11873580001) and centrifuged (12,000 × *g*, 10 min, 4 °C). The supernatants containing lactoferricin and MMP9 were then collected and measured using ELISA kits. Similarly, we assessed azurophil granule release and NETosis release by neutrophils using ELISA kits for MPO (LifeSpan, F4305) and NE (LifeSpan, F20775).

### Assays of neutrophil cytokine release in vitro

To stimulate cytokine release, 1 × 10^6^ freshly-isolated peripheral neutrophils were incubated with LPS (2 μg/mL) in RPMI 1640 with 1% BSA for 18 h. The level of cytokine release in the cell-free supernatants was determined using ELISA kits for TNF-α (LifeSpan, F12799) and IL-1β (Abbkine, KET9001) according to the manufacturers’ instructions.

### Isolation of neonatal rat ventricular cardiomyocytes (NRVMs)

NRVMs were isolated from postnatal day 1 hearts according to a previously described method^22^. Briefly, the ventricles of 1-day-old Sprague-Dawley rat pups were dissected and washed with HBSS without Ca^2+^ and Mg^2+^ (MacGene, CC016). Using micro-dissecting scissors, ventricular tissue was minced to obtain ~1 mm^3^ pieces, which were then treated with 5 mL of digestion solution containing collagenase II (0.3 mg/mL; Thermo, 17101015) and trypsin (1 mg/mL; Amresco, VWRV0785) in HBSS for 5 min at 37 °C. The resulting supernatants were removed and the residual tissue was repeatedly treated with the digestion solution until little remained. The cells in the supernatants were transferred to a tube with an equal volume of ice-cold DMEM containing 10% FBS and 1% penicillin–streptomycin and centrifuged at 1000 × *g* for 4 min at room temperature. The cell pellets were re-suspended in 25 mL DMEM containing 5% horse serum (MacGene, CS008), 1% penicillin–streptomycin, and 1 μmol/L cytosine arabinoside, and then incubated in a 100-mm dish for 1.5 h at 37 °C to eliminate fibroblast contamination. Non-adherent cells were collected and seeded at a final concentration of 5 × 10^5^ cells/mL. After incubation for 48 h, the medium was removed and the NRVMs were then cultured with DMEM containing 10% FBS and 1% penicillin-streptomycin. To co-culture NRVMs and neutrophils, equal numbers of ABNs and NRVMs were mixed and then incubated at 3 °C for 12 h before performing cell viability assays.

### Rat bone marrow transplantation

Rat bone marrow cell transplantation was performed as previously described with minor modifications^75^. Briefly, WT rats were exposed to 10 Gy of Co^60^ radiation to completely deplete the hematopoietic system, and within 4 h of radiation were given an intravenous injection of bone marrow cells (at least 1 × 10^8^ cells per rat) harvested from healthy WT or *Dusp6*-deficient rats. The outcome of hematopoietic depletion by radiation and recovery by transplantation were determined by counting the Giemsa-stained whole blood cells collected from the tail vein.

### Cell staining and flow cytometry

Single-cell suspensions of infarcted cardiac tissues were obtained by digestion with Liberase DL (Roche, 5401160001) for cardiac cells, neutrophils, and macrophages; single-T-cell suspensions were obtained by smashing mediastinal lymph nodes (MLNs) through 40 μm filters. To isolate total peripheral leukocytes, blood samples were processed by sedimentation with 6% Hydroxyethyl starch (TBD Science, HES-TBD550) and erythrocyte lysis with ACK Lysis Buffer (TBD Science, NH4CL2009) successively, then were stained with the following fluorescence-labeled antibodies against cell surface markers: anti-rat CD45-PE-Cy7 (1:300, clone OX-1, BD Biosciences, 561588), anti-rat granulocyte-FITC (1:300, clone HIS48, BD Biosciences, 554907), anti-rat macrophage-PE (1:300, clone HIS36, eBioscience, 12-0660-82), anti-rat CD11b-PE (1:300, clone WT.5, BD Biosciences, 562105), anti-rat CD31-FITC (1:200, clone TLD-3A12, Abd Serotec, MCA1334F), anti-rat CD3-APC (1:300, clone 1F4, BD Biosciences, 557030), anti-rat CD4-PE (1:300, clone OX-35, BD Biosciences, 561833), anti-rat CD8a-FITC (1:300, clone OX-8, BD Biosciences, 559976), anti-rat CD25-Alexa Flour 647 (1:300, clone OX-39, Abd Serotec, MCA273A647), anti-rat CD45RA-PE (1:300, clone OX-33, BD Biosciences, 551402), anti-rat CD161-PE (1:300, clone 10/78, BD Biosciences, 555009) and anti-rat CD86-Alexa Fluor 647 (1:300, clone 24F, Abd Serotec, MCA2874A647). These antibodies were diluted in Flow Cytometry Staining Buffer (eBioscience, 00-4222-26). For intracellular protein staining, cells were fixed and permeabilized with Leucoperm^TM^ (Abd Serotec, BUF09) and then stained with the following primary antibodies: anti-DUSP6 (1:200, clone EPR129Y, Abcam, ab76310), anti-cTnT (1:200, clone 1C11, Abcam, ab8295), anti-α-SMA-FITC (1:200, clone 1A4, Abcam, ab184675), anti-desmin (1:200, clone Y66, Abcam, ab32362), anti-TNF-α-PE (1:300, clone TN3-19.12, BD Biosciences, 559503), anti-IL-1β (1:200, Abcam, ab9722), anti-TGF-β (1:300, R&D, AB-100-NA). The secondary antibodies were Alexa Fluor 647 donkey anti-rabbit IgG (1:1000, Thermo Fisher, A31573), and Alexa Fluor 488 goat anti-mouse IgG (1:1000, Thermo Fisher, A11029). FOXP3/Transcription Factor Staining Buffer Set Kit (Invitrogen, 00-5523-00) and anti-rat FOXP3-PE (1:300, clone FJK-16s, Invitrogen, 12-5773-80) were used for FOXP3 labeling. Flow cytometry was executed on BD Biosciences FACSVerse^TM^ and LSRFortessa^TM^ flow cytometers with BD FACSuite software. The cardiac macrophages were gated by CD45^+^/HIS36^+^ and sorted on MoFlo XDP Cell Sorter (Beckman Coulter).

#### Protein preparation and western blot

Rat heart tissue, isolated neutrophils, and other cells were homogenized in RIPA buffer (Applygen, C1053) in the presence of 1 mM PMSF (Sigma, 93482), protease inhibitor cocktail (Roche, 11873580001), and PhosSTOP phosphatase inhibitor cocktail (Roche, 04906845001). After centrifugation (12,000 × *g*, 10 min, 4 °C), protein extracts were quantified with BCA Protein Assay Kits (Applygen, P1511), then resolved on 12% SDS-PAGE gels, and transferred onto PVDF membranes (Millipore, IPVH00010). Membrane blots were incubated overnight with the appropriate primary antibody, followed by incubation with the corresponding HRP-conjugated secondary antibody for 2 h at room temperature, and protein bands were detected with a SuperEnhanced Chemiluminescence Detection Kit (Applygen, P1010). The primary antibodies were: anti-GAPDH (1:5000, Easybio, BE0023), anti-β-actin (1:5000, Easybio, BE0021), anti-DUSP6 (1:1000, clone EPR129Y, Abcam, ab76310), anti-ERK (1:5000, clone 137F5, CST, 4695), anti-pERK (1:1000, clone D13.14.4E, CST, 4370), anti-p38 (1:5000, clone D13E1, CST, 8690), anti-p-p38 (1:1000, clone D3F9, CST, 4511), anti-JNK (1:1000, clone EPR16797-211, Abcam, ab179461), anti-pJNK (1:500, clone EP1597Y, Abcam, ab76572), anti-BAX (1:1000, CST, 2772), anti-BCL-2 (1:1000, CST, 2870), anti-DUSP1 (1:1000, Millipore, 07-935), and anti-DUSP16 (1:1000, Biorbyt, orb215305). Goat anti-rabbit IgG–HRP (1:5000, Easybio, BE0101) and goat anti-mouse IgG–HRP (1:5000, Easybio, BE0102) were the secondary antibodies. PageRuler™ Prestained Protein Ladder (Thermo Fisher, 26616) was used as a standard protein marker. Imaging was performed on ChemiDoc^TM^ XRS + System with Quantity-One software (Bio-Rad).

### Cell smear preparation and Giemsa staining

Total peripheral leukocytes, isolated polymorphonuclear granulocytes, and mononuclear cells prepared by centrifugation (700 × *g*, 4 min) were smeared on glass slides. For Giemsa staining, cell smears were fixed in methanol for 10 min at room temperature and then stained with the Wright-Giemsa Stain Buffer (BASO, BA-4034). The nuclei stained purple.

#### RT-PCR analysis

Total RNA was extracted using TRI Reagent (Sigma, T9424) and purified using an RNeasy mini kit (Qiagen, 74106), and ~1 μg RNA was used for reverse transcription with SuperScript™ II Reverse Transcriptase (Thermo Fisher, 18064). Quantitative real-time PCR reactions were performed using SYBR^@^ Premix DimerEraser™ (Takara, RR091A) on StepOnePlus^TM^ Real-time PCR system with StepOne v2.3 software (Thermo Fisher). Primer sequences are listed in Supplementary Table l.

### RNA-seq analysis

RNA-seq library preparation, sequencing and data analysis were performed by Novogene (Beijing, China). Briefly, total amounts and integrity of RNA samples were assessed using RNA Nano 6000 Assay Kit of Bioanalyzer 2100 system (Agilent Technologies, CA, USA). RNA libraries were prepared using KAPA RNA HyperPrep Kit (8098140702, Roche) and sequenced by Illumina NovaSeq 6000. Raw reads were trimmed with Trim Galore and then aligned to rat reference genome (rnor6) by HISAT2^76^. The featureCounts^77^ were used to count the reads number mapped to each gene. And FPKM of each gene was calculated based on the length of the gene and reads count mapped to this gene. Differential expression analysis of two conditions (WT and *Dusp6*-deficient) was performed using DEseq2^78^. Cutoffs of FDR < 0.05 and log2 fold change >1 were used for differentially expressed genes. Generation of the volcano plot and heatmaps, as well as Gene ontology analysis were performed using iDEP.94^79^.

### Chromatin immunoprecipitation

Rat neutrophil chromatin was isolated and ChIP assays were performed using a Pierce™ Magnetic ChIP Kit (Thermo, 26157) as previously described^80^. Chromatin was immunoprecipitated using anti-C/EBPβ antibody (1:50, Cell Signaling Technology, 3082), as well as Normal Rabbit IgG (1:1000, Cell Signaling Technology, 2729) and anti-Histone H3 antibody (1:1000, clone D2B12, Cell Signaling Technology, 4620) for negative and positive controls, respectively. C/EBPβ-binding sequences were amplified with the respective gene primers listed in Supplementary Table 1.

### Luciferase assays

The full-length cDNA of rat C/EBPβ was acquired from the total RNA of isolated neutrophils by RT-PCR, and inserted into pcDNA4/myc-HisB to construct the final C/EBPβ expression vector. The rat *Dusp6* promoter fragment (from –2030 to –1284) was cloned into the pGL3-Basic vector to form pGL3-*Dusp6*-Luc, and the mutant *Dusp6* promoter was synthesized to substitute all putative C/EBPβ-binding sites with AGT and cloned into the pGL3-Basic vector. For luciferase assays, 293T cells were cultured in 6-well plates and transfected with pGL3-*Dusp6*-Luc (1 μg) and pREP4-*Renilla* (20 ng), and simultaneously co-infected with 1.5 μg of either pcDNA-C/EBPβ or pEGFP-N1 (as negative control) by using FuGENE® HD Transfection Reagent (Promega, E2311). Cell lysis and luciferase activity assays were carried out 48 h after transfection using a Dual-Luciferase® Reporter Assay System (Promega, E1910) as previously reported^12^.

### Statistical analysis

The results of Masson’s staining, TTC-Evans blue staining, immunostaining, and Western blot were analyzed using Image-Pro Plus and ImageJ. All statistics were calculated using Graphpad Prism 8. The data are reported as mean ± SD. The significance of differences between two groups was determined using Two-sided unpaired T-test. Among three or more groups, One-way ANOVA with Tukey’s multiple comparison test was used for comparisons. A *p* value less than 0.05 was referred as statistically significant.

### Reporting summary

Further information on research design is available in the Nature Research Reporting Summary linked to this article.

## Supplementary information

Supplementary Information

Reporting Summary

## Data Availability

The data for the expression levels of DUSP6 RNA in multiple human cell types were obtained frrom Human Protein Atlas (https://www.proteinatlas.org/ENSG00000139318-DUSP6/single+cell+type). The RNA-seq datasets of cardiac macrophages generated in this study have been deposited in Gene Expression Omnibus (GEO) under accession code GSE195978. The remaining data in this study are available within the article, Supplementary Information and source data file. Source data are provided with this paper.

## Source data

Source Data
