# Supplementary material for: Dusp6 deficiency attenuates neutrophil-mediated cardiac damage in the acute inflammatory phase of myocardial infarction
Source: Nat Commun. 2022 Nov 5;13:6672. doi: 10.1038/s41467-022-33631-z (PMC9637103; doi:10.1038/s41467-022-33631-z)
Supplement: Supplementary file 1 — Supplementary Information [file 41467_2022_33631_MOESM1_ESM.pdf]

Supplementary Information

Dusp6 deficiency attenuates neutrophil-mediated cardiac damage in the acute inflammatory phase of myocardial infarction

Xiaohai Zhou[1], Chenyang Zhang[1,2], Xueying Wu[1], Xinli Hu[1], Yan Zhang[1], Xuelian Wang[1], Lixia Zheng[1], Peng Gao[1], Jianyong Du[1], Wen Zheng[1], Haibao Shang[1], Keping Hu[3], Zhengfan Jiang[4], Yu Nie[5], Shengshou Hu[5], Rui-Ping Xiao[1,2], Xiaojun Zhu[1,2*] & Jing-Wei Xiong[1,2*]

Contents:

1) Supplementary Figures 1-21 and Figure Legends;
2) Uncropped blots in Supplementary Figures (Supplementary Figures 22-28);
3) Supplementary Tables 1-3

Supplementary Figures and Figure Legends

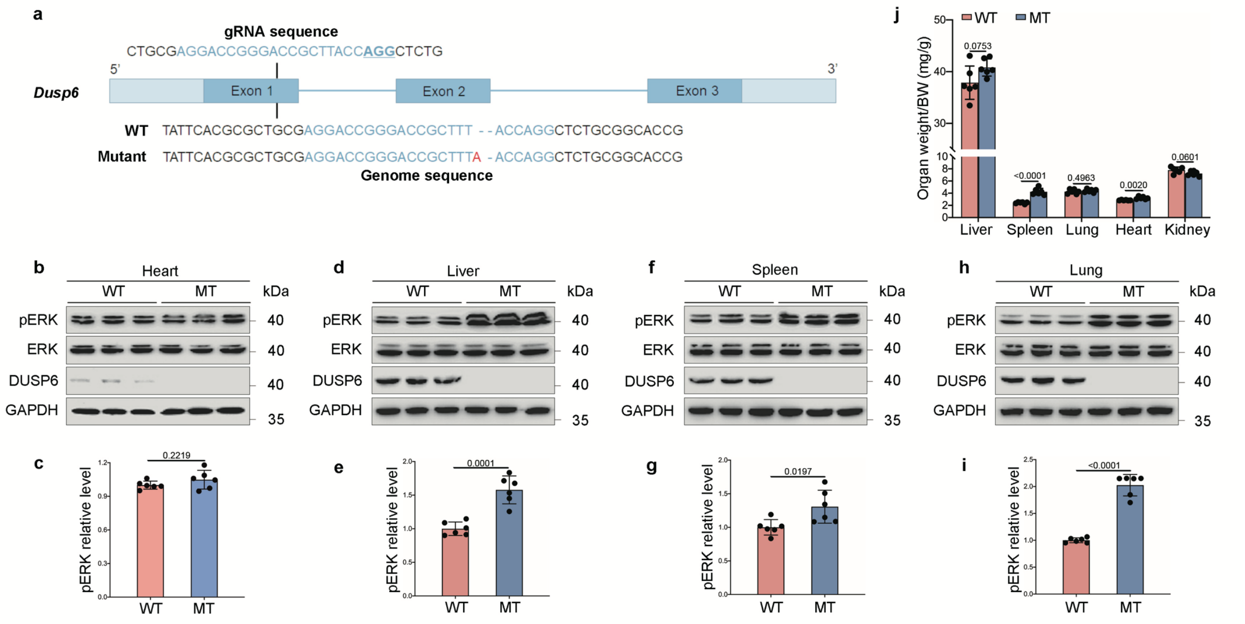

Supplementary Figure 1. *Dusp6* deficiency enhances ERK phosphorylation in the rat liver, spleen and lung but not the heart. (a) Schematic of the *Dusp6* mutant allele generated by CRISPR/gRNA in rats, showing the insertion of an adenine nucleotide in exon 1 of the *Dusp6* gene. **(b-i)** Western blots and corresponding quantitative analysis showing DUSP6 expression and ERK phosphorylation (pERK) levels in WT and *Dusp6* mutant rat heart (**b** and **c**), liver (**d** and **e**), spleen (**f** and **g**), and lung (**h** and **I**). GAPDH and ERK served as internal references. n = 6/group. All blots within each individual panel were performed in parallel with the same samples. **(j)** Quantitative measurements on the ratio of organ weight/body weight in WT and *Dusp6* mutant rats. n = 6/group. All quantitative data shown in this figure are presented as mean values ± SD. Two-sided unpaired T-test was used to calculate the presented p-values. Source data of **c**, **e**, **g**, **i** and **j** are provided in a Source Data File. WT: wild type; MT: *Dusp6* mutant.

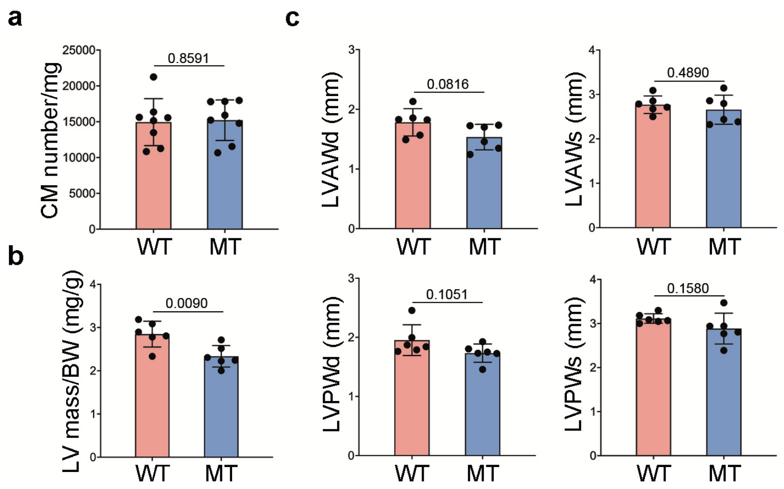

Supplementary Figure 2. *Dusp6* deficiency has no effect on the CM number, tissue mass and wall thickness of the LV. (a) Quantification of the numbers of CMs in 1 mg LV tissues from WT and *Dusp6*-deficient rats at 2 months old. n=8/group. **(b-c)** LV mass **(b)** and anterior/posterior wall thickness at diastole and systole **(c)** in WT and *Dusp6*-deficient rats at 2 months old evaluated by echocardiography. n=6/group. All quantitative data shown in this figure are presented as mean values ± SD. Two-sided unpaired T-test was used to calculate the presented p-values. Source data of **a**, **b** and **c** are provided in a Source Data File. WT: wild type; MT: *Dusp6* mutant; CM: cardiomyocytes; LV: left ventricle; BW: body weight; LVAWd: LV anterior wall thickness at diastole; LVAWs: LV anterior wall thickness at systole; LVPWd: LV posterior wall thickness at diastole; LVPWs: LV posterior wall thickness at systole.

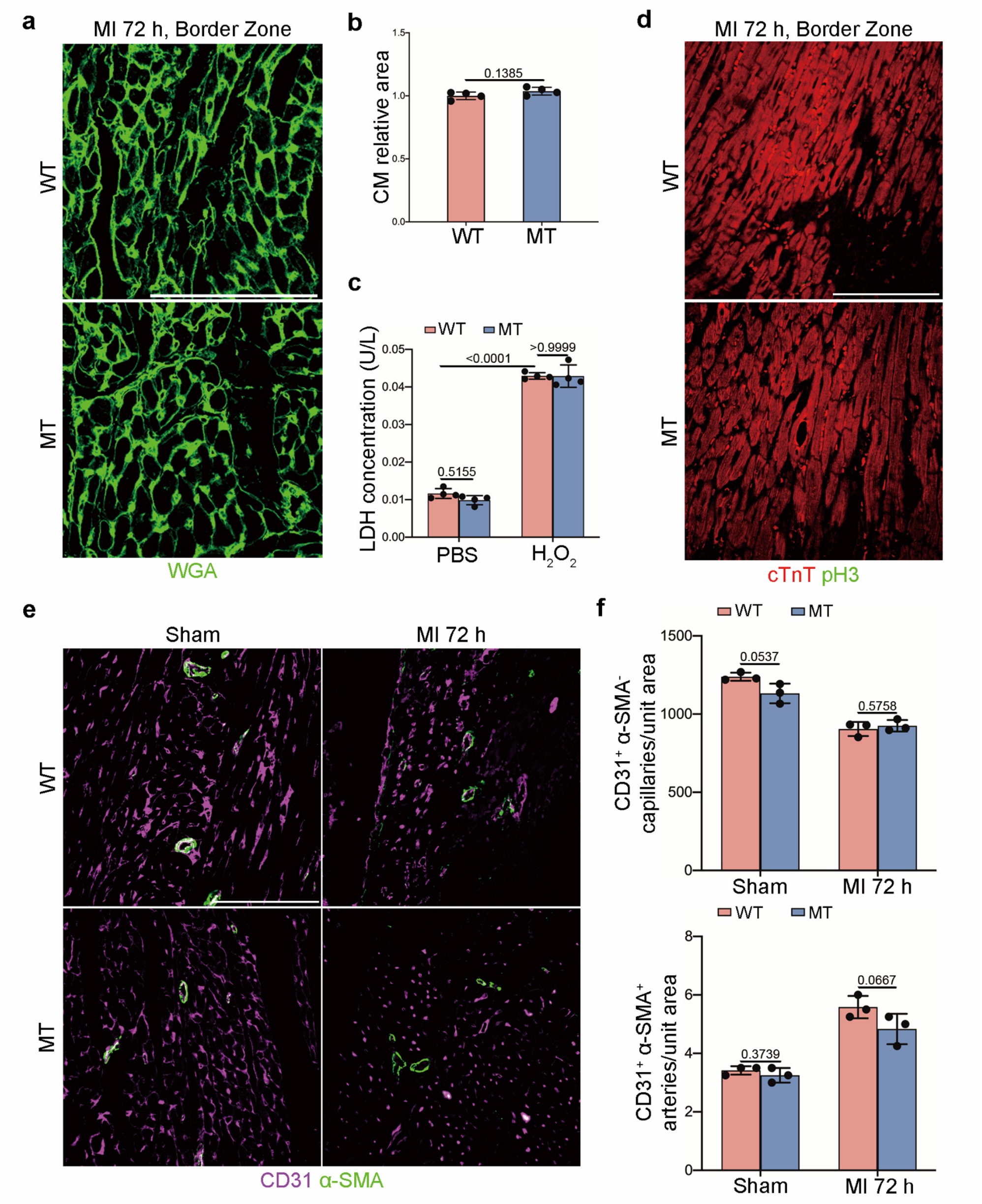

Supplementary Figure 3. *Dusp6* deficiency has no effect on CM hypertrophy, apoptosis and proliferation as well as angiogenesis after MI. (a-b) WGA staining and corresponding quantitative analysis of CM area in WT and *Dusp6*-deficient LV tissue at 72 h after MI (n = 4 biological independent samples/group; scale bar, 100 μm). **(c)** Quantitative analysis of LDH release of WT and *Dusp6* mutant NRVMs in the presence and absence of H_2O_2. n = 4/group. **(d)** Immunostaining of pH3 in WT and *Dusp6*-deficient LV tissue at 72 h after MI. cTnT staining was used to label

cardiomyocytes (n = 3 biological independent samples/group; scale bar, 100 μm). **(e-f)** Double immunofluorescence staining for CD31 and α-SMA and the numbers of $CD31^{+}$α-SMA^{-} capillaries and $CD31^{+}$α-SMA^{+} arteries in WT and *Dusp6*-deficient LV tissue in sham-operated rats and 72 h after MI (n = 3 biological independent samples/group; scale bar, 100 μm). All quantitative data shown in this figure are presented as mean values ± SD. One-way ANOVA with Tukey's multiple comparison test (for **c**) and Two-sided unpaired T-test (for **b** and **f**) were used to calculate the presented p-values. Source data of **b**, **c** and **f** are provided in a Source Data File. WT: wild type; MT: *Dusp6* mutant; CM, cardiomyocyte; WGA, wheat germ agglutinin; LDH, lactate dehydrogenase; NRVMs, neonatal rat ventricular myocytes; α-SMA, α-smooth muscle actin; pH3, phosphorylated histone H3; cTnT, cardiac troponin T.

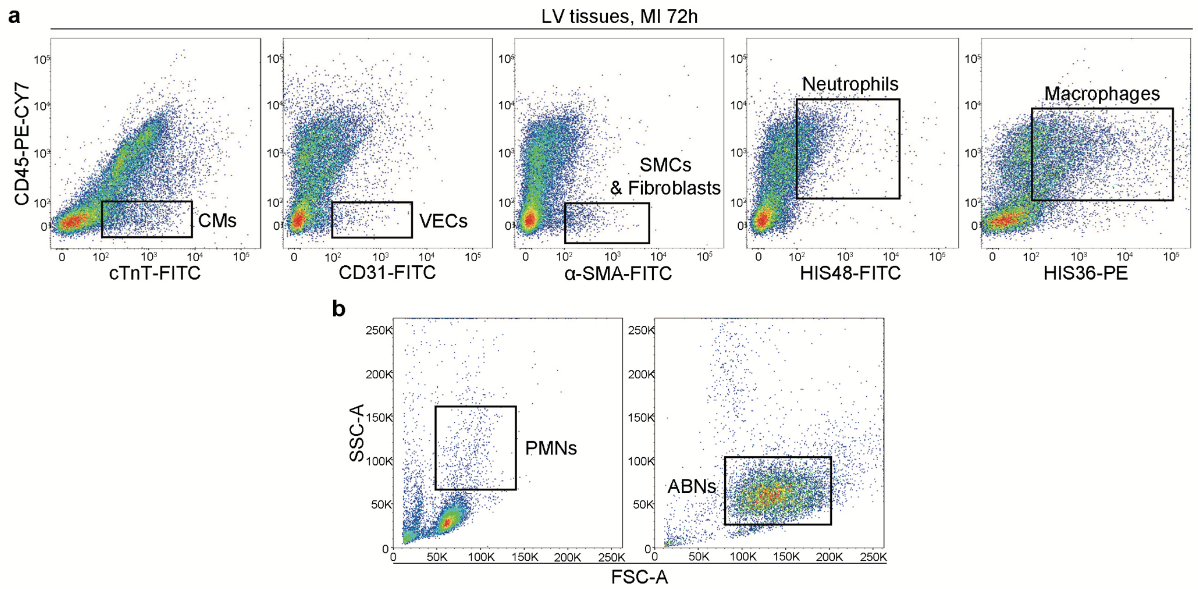

Supplementary Figure 4. Gating strategy for different cell populations from LV tissue and PBLs. (a) Representative gating images of flow cytometry analysis for single-cell populations from WT LV tissue at 72 h after MI using the pan-leukocyte cell surface marker CD45, cTnT for cardiomyocytes (CMs), CD31 for vascular endothelial cells (VECs), α-SMA for smooth muscle cells (SMCs) and fibroblasts, HIS48 for neutrophils, and HIS36 for macrophages. **(b)** Representative grouping of PMNs and ABNs by FSC/SSC in flow cytometry. PBLs, peripheral blood leukocytes; PMNs, polymorphonuclear cells (peripheral neutrophils); ABNs, abdominal neutrophils; FSC-A, forward scatter channel-area; SSC-A, side scatter channel-area.

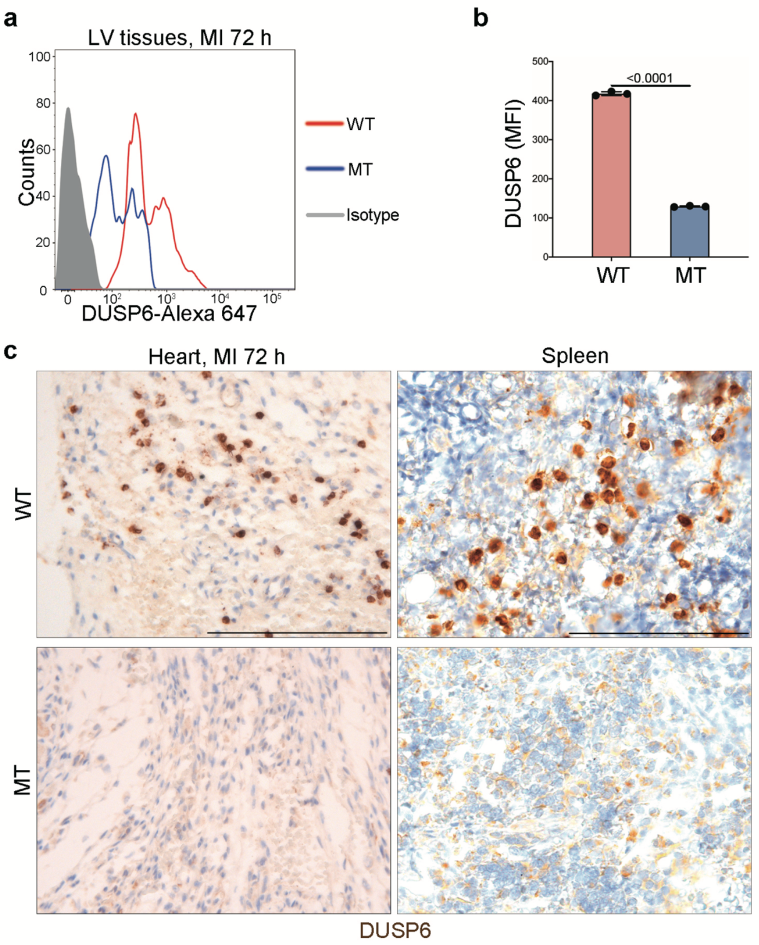

Supplementary Figure 5. Specificity of DUSP6 antibodies for flow cytometry and immunostaining assays. (a-b) Intracellular staining, flow cytometry and corresponding quantitative analysis of DUSP6 in single-cell suspensions from WT and *Dusp6*-deficient LV tissues at 72 h after MI using DUSP6 antibody 1 (Abcam, ab76310). n = 3 biological independent samples/group. **(c)** Representative immunohistochemistry images and high-magnification views of Dusp6 protein expression in WT and *Dusp6*-deficient spleens and LV tissues at 72 h after MI using DUSP6 antibody 2 (Origene, TA323084). n = 3 biological independent samples/group; scale bar: 100 μm. All quantitative data shown in this figure are presented as mean values ± SD. Two-sided unpaired T-test

was used to calculate the presented p-values. Source data of **b** are provided in a Source Data File. WT: wild type; MT: *Dusp6* mutant.

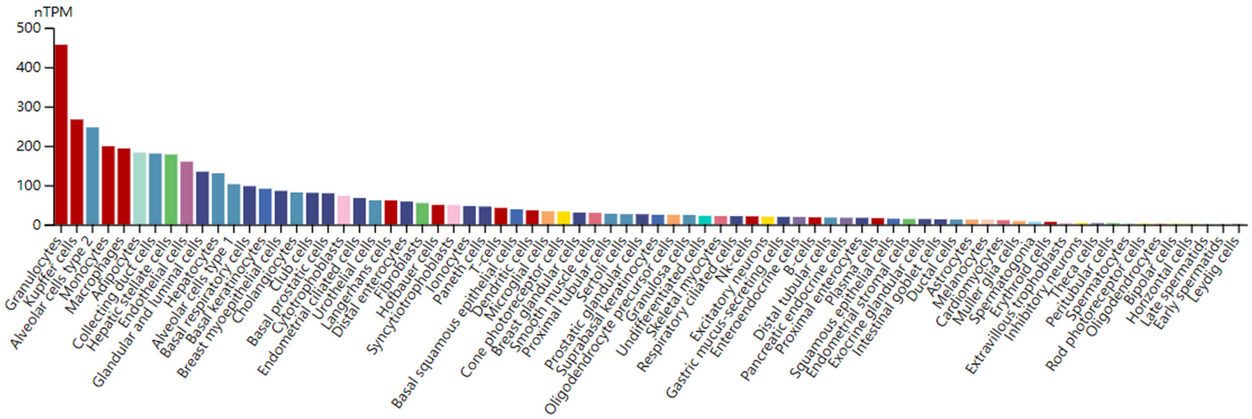

Supplementary Figure 6. Expression levels of *DUSP6* RNA in multiple human cell types (The Human Protein Atlas data). Granulocytes present the strongest *DUSP6* level among these cells, whereas the main cell subsets making up cardiac tissues, including cardiomyocytes, fibroblasts, endothelial cells and smooth muscle cells display moderate or weak *DUSP6* expression.

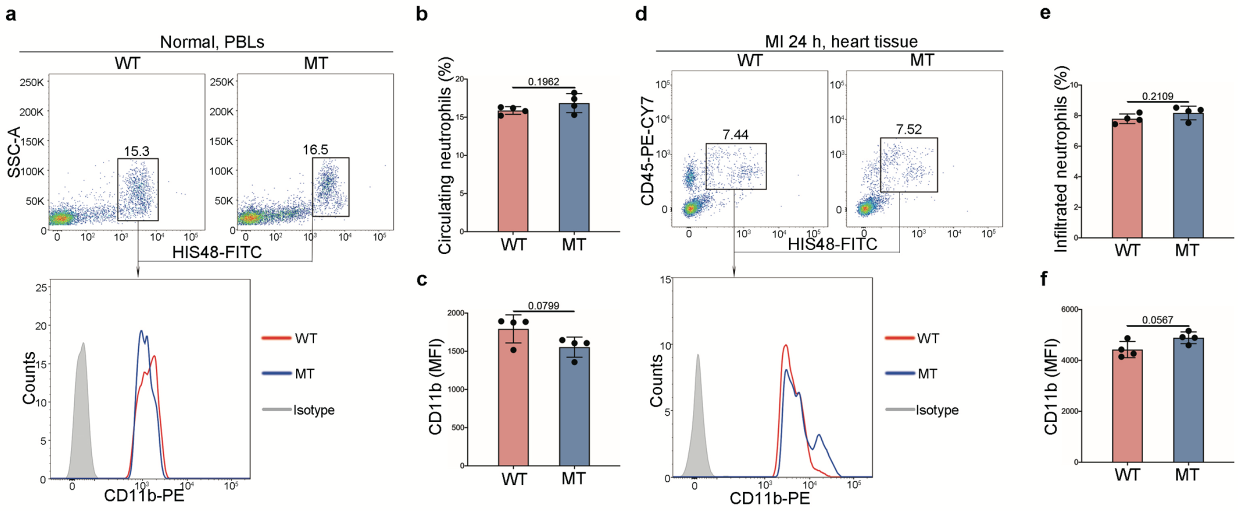

Supplementary Figure 7. *Dusp6* deficiency has no effect on neutrophil development, differentiation, and chemotaxis. (a-c) Flow cytometry with HIS48 and CD11b antibody and corresponding quantitative analysis of HIS48$^+$ neutrophils in WT and *Dusp6*-deficient PBLs, as well as the CD11b expression level in WT and *Dusp6*-deficient HIS48$^+$ neutrophils. **(d-f)** Flow cytometry and corresponding quantitative analysis of infiltrated CD45$^+$HIS48$^+$ neutrophils from WT and *Dusp6*-deficient LV tissue at 24 h after MI, as well as the CD11b expression level in WT and *Dusp6*-deficient infiltrated CD45$^+$HIS48$^+$ neutrophils. n = 4 biological independent samples/group. All quantitative data shown in this figure are presented as mean values ± SD. Two-sided unpaired T-test was used to calculate the presented p-values. Source data of **b**, **c**, **e** and **f** are provided in a Source Data File. WT: wild type; MT: *Dusp6* mutant.

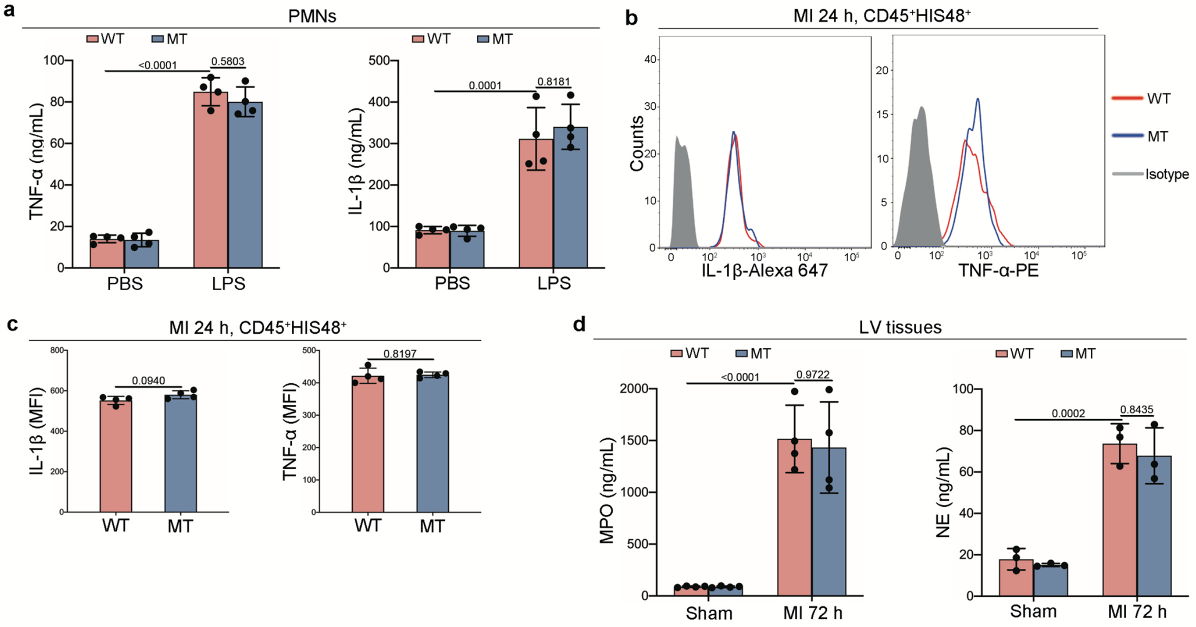

Supplementary Figure 8. Release of inflammatory cytokines, azurophilic granules, and NETs are not affected in *Dusp6*-deficient neutrophils. (a) ELISA analysis of TNF-α and IL-1β release in WT and *Dusp6*-deficient PMNs with either PBS or LPS stimulation (n = 4/group). **(b-c)** Flow cytometric sorting and corresponding quantitative analysis of IL-1β and TNF-α in $CD45^{+}HIS48^{+}$ neutrophils from WT and *Dusp6*-deficient LV tissue at 24 h after MI (n = 4/group). **(d)** ELISA analysis of MPO (n = 4/group) and NE (n = 3/group) in WT and *Dusp6*-deficient LV tissue from sham-operated rats and 72 h after MI. All quantitative data shown in this figure are presented as mean values ± SD. One-way ANOVA with Tukey's multiple comparison test (for **a** and **d**) and Two-sided unpaired T-test (for **c**) were used to calculate the presented p-values. Source data of **a**, **c** and **d** are provided in a Source Data File. WT: wild type; MT: *Dusp6* mutant; LPS, lipopolysaccharide; IL-1β, interleukin-1β; TNF-α, tumor necrosis factor-α; MPO, myeloperoxidase; NE, neutrophil elastase.

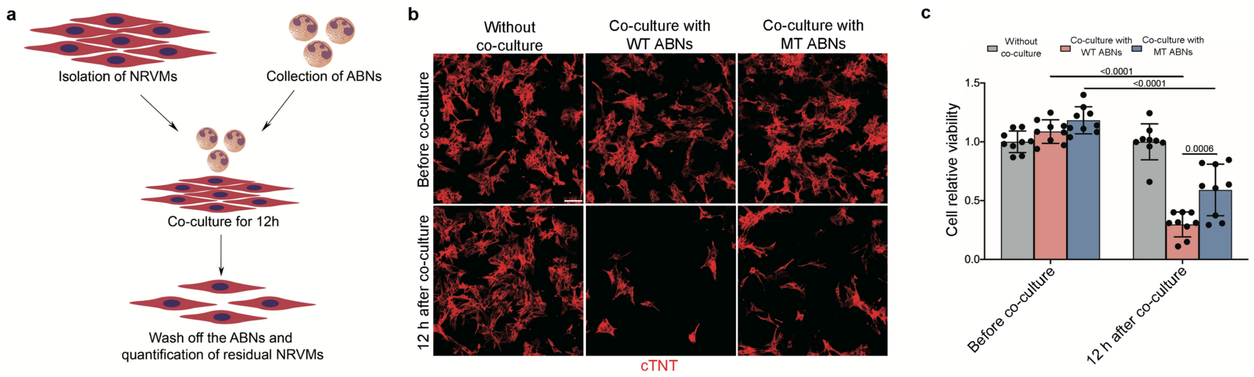

Supplementary Figure 9. *Dusp6* deficiency in neutrophils attenuates cardiac damage in vitro. (a) Schematic of in vitro assay of neutrophil killing-activity against cardiomyocytes. NRVMs from WT neonatal rats alone, NRVMs and ABNs from WT or *Dusp6*-deficient rats were co-cultured for 12 h. The numbers of viable NRVMs were determined by cTnT immunostaining and cell viability assays. **(b)** Immunostaining of cTnT in NRVMs alone, with WT ABNs or Dusp6 mutant ABNs before co-culture and 12 h after co-culture (scale bar, 50 μm). **(c)** Cell viability assays for NRVMs before and after co-culture as in **b** (n = 9/group). All quantitative data shown in this figure are presented as mean values ± SD. One-way ANOVA with Tukey's multiple comparison test was used to calculate the presented p-values. Source data of **c** are provided in a Source Data File. WT: wild type; MT: *Dusp6* mutant; NRVMs, neonatal rat ventricular myocytes; cTnT: cardiac Troponin T.

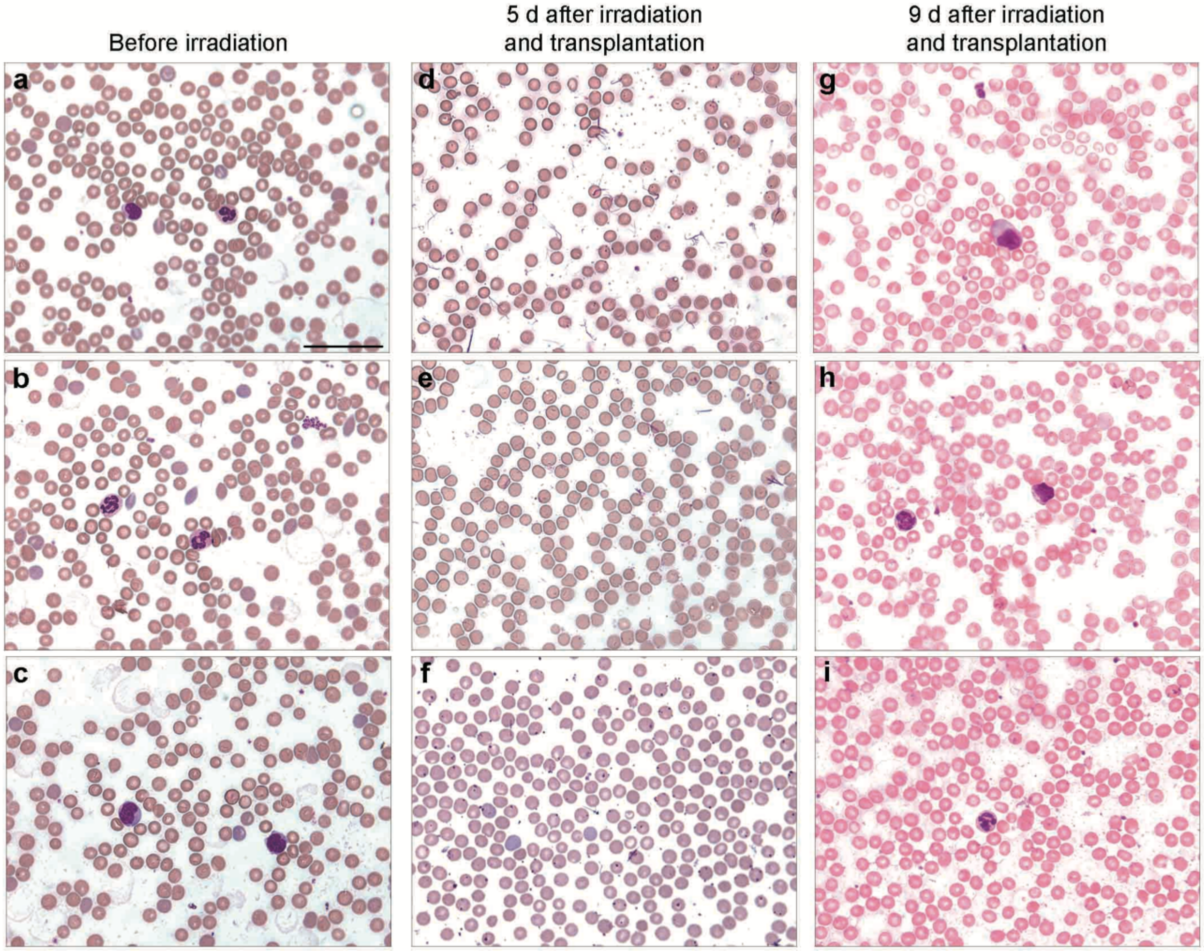

Supplementary Figure 10. Dynamic changes of peripheral blood leukocytes (PBLs) before and after bone marrow cell transplantation. Giemsa staining of rat whole blood collected from the tail vein before irradiation **(a-c),** and 5 days **(d-f)** and 9 days **(g-i)** after transplantation, showing complete depletion of leukocytes at 5 days after irradiation and recovery of new leukocytes at 9 days after irradiation and bone marrow transplantation. The nuclei of PBLs stained purple, distinguishing them from red-stained erythrocytes. n = 6 rats/group. Scale bar: 100 μm.

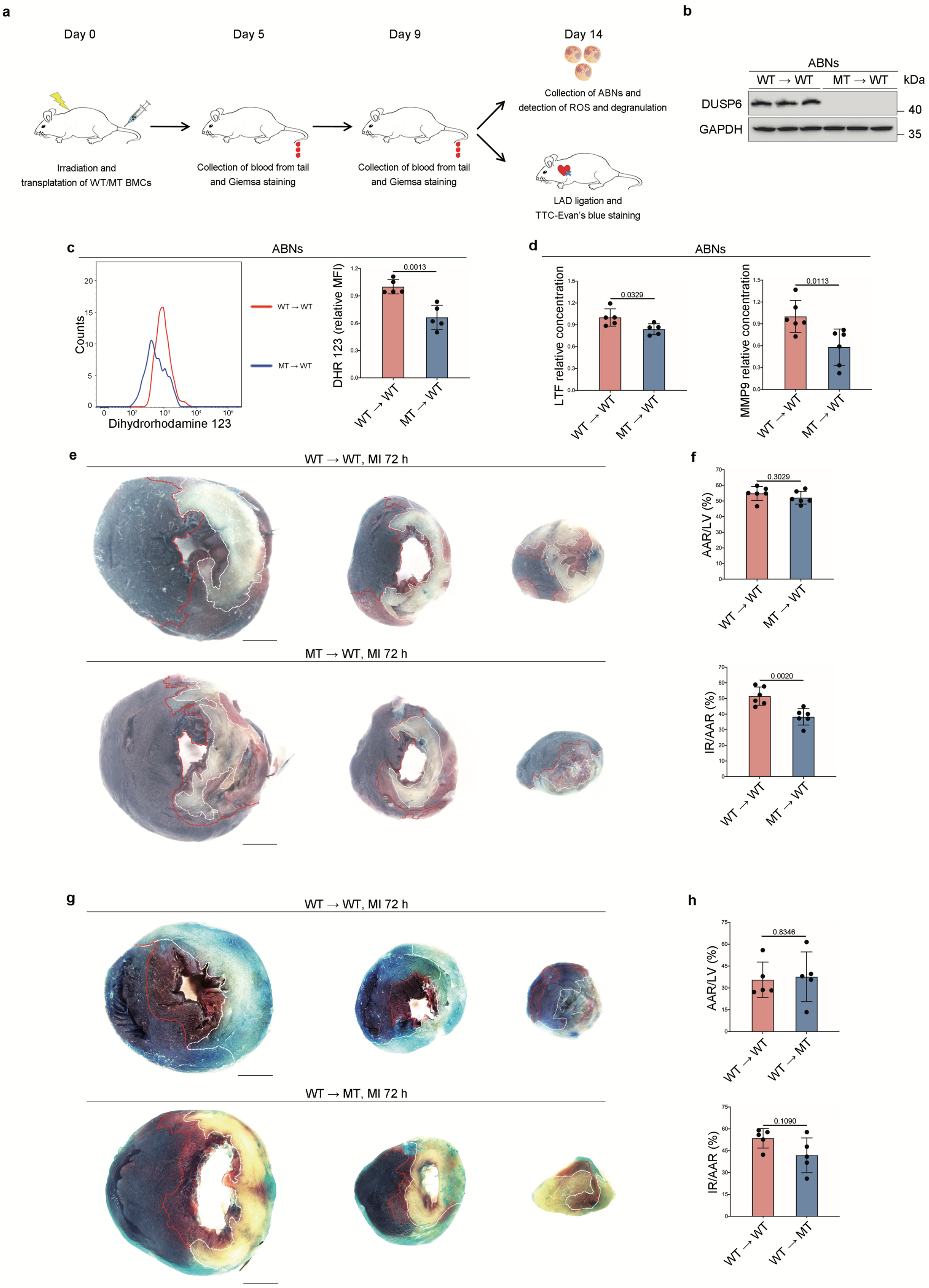
a
Day 0
Day 5
Day 9
Day 14
Irradiation and
transplatation of WT/MT BMCs
Collection of blood from tail
and Giemsa staining
Collection of blood from tail
and Giemsa staining
Collection of ABNs and
detection of ROS and degranulation
LAD ligation and
TTC-Evan's blue staining
b
ABNs
WT → WT
MT → WT
kDa
DUSP6
GAPDH
40
35
c
ABNs
Counts
Dihydrorhodamine 123
WT → WT
MT → WT
DHR 123 (relative MFI)
0.0013
d
ABNs
LTF relative concentration
0.0329
MMP9 relative concentration
0.0113
e
WT → WT, MI 72 h
MT → WT, MI 72 h
f
AAR/LV (%)
0.3029
IR/AAR (%)
0.0020
g
WT → WT, MI 72 h
WT → MT, MI 72 h
h
AAR/LV (%)
0.8346
IR/AAR (%)
0.1090
WT → WT
WT → MT

Supplementary Figure 11. Reduction of neutrophil-mediated cardiac damage after MI by transplantation of *Dusp6*-deficient bone marrow cells. (a) Schematic of rat bone marrow cell (BMC) transplantation and subsequent assay for neutrophil function and post-MI cardiac damage at different time points. **(b)** Western blot of DUSP6 in ABNs from irradiated WT rats receiving WT (WT → WT) or Dusp6-deficient (MT → WT) BMC transplantation. GAPDH served as internal control (n = 3 biological independent samples/group). Both blots were performed in parallel with the same samples. **(c-d)** DHR 123 staining assays of ROS production **(c)** and ELISA analysis of LTF and MMP9 release **(d)** in ABNs from WT → WT and MT → WT rats (n = 5 biological independent samples/group). **(e-h)** TTC-Evans blue staining and corresponding quantitative analysis of the infarct area in hearts from MT → WT **(e-f)** and WT → MT **(g-h)** rats at 72 h after MI. WT → WT rats were used as controls. n = 6 rats/group for **e-f**; n = 5 rats/group for **g-h**; scale bar, 5 mm. All quantitative data shown in this figure are presented as mean values ± SD. Two-sided unpaired T-test was used to calculate the presented p-values. Source data of **c**, **d**, **f** and **h** are provided in a Source Data File. WT: wild type; MT: *Dusp6* mutant.

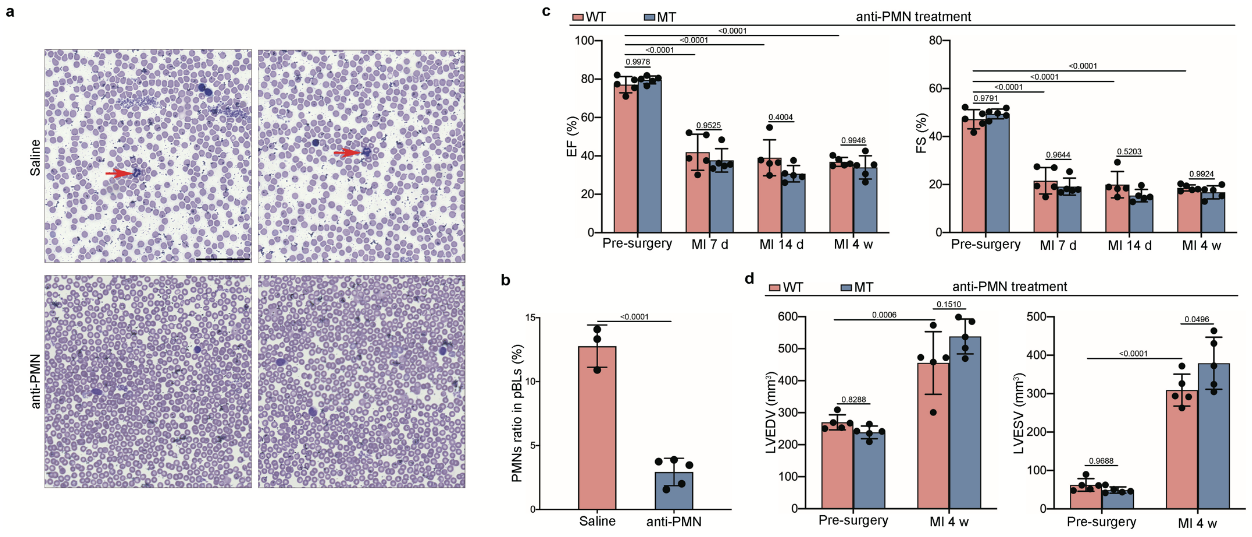

Supplementary Figure 12. Depletion of peripheral neutrophils ablates the improvement of cardiac function in *Dusp6*-deficient rats post-MI. (a-b) Giemsa staining **(a)** and corresponding quantitative analysis **(b)** of PMNs in whole blood collected from rats injected with anti-PMN antibody (250 μL/kg) or saline (as control) for three consecutive days. n = 3 animals for saline group and 5 animals for anti-PMN group. PMNs were indicated by the red arrows. Scale bar: 100 μm. **(c)** Echocardiographic measurements of EF and FS for neutrophil-depleted WT and *Dusp6*-deficient rats before surgery, as well as 7 days, 14 days and 4 weeks after MI. **(d)** Echocardiographic measurements of LVEDV and LVESV for neutrophil-depleted WT and Dusp6-deficient rats before surgery and 4 weeks after MI. n = 5 rats/group. One-way ANOVA with Tukey's multiple comparison test (for **c** and **d**) and Two-sided unpaired T-test (for **b**) were used to calculate the presented p-values. Source data of **b**, **c** and **d** are provided in a Source Data File. WT: wild type; MT: *Dusp6* mutant; PMNs: polymorphonuclear cells (peripheral neutrophils).

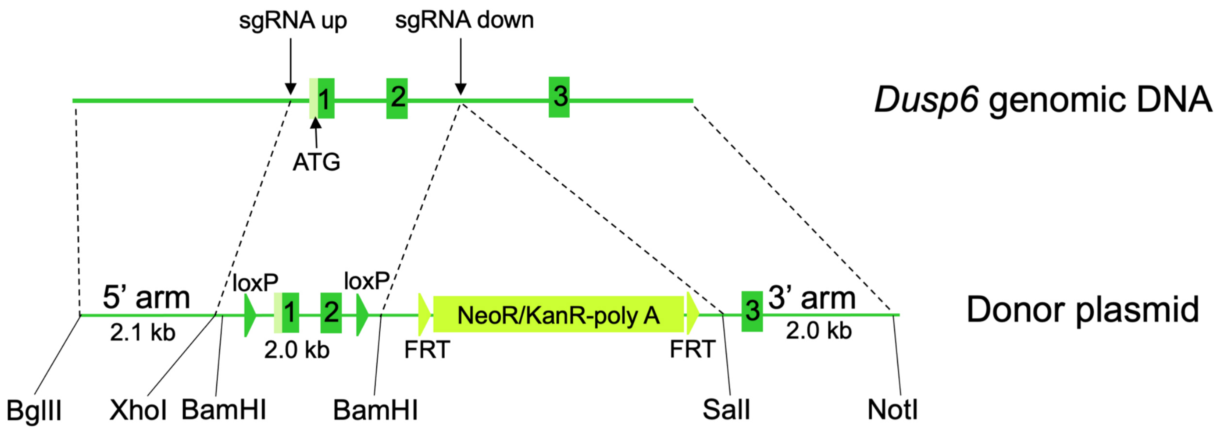

Supplementary Figure 13. Generation of Dusp6-floxed mice. Two single-guide RNAs (sgRNA) were designed to facilitate Cas9 nuclease to target the upstream of exon 1 and the downstream of exon 2, respectively. The targeting donor vector containing the sequences of exon 1 and 2 flanked by two loxP sites, as well as 5' and 3' homologous arms is shown at the bottom. The NeoR/KanR-poly A sequence was removed by using the Flp delete mice.

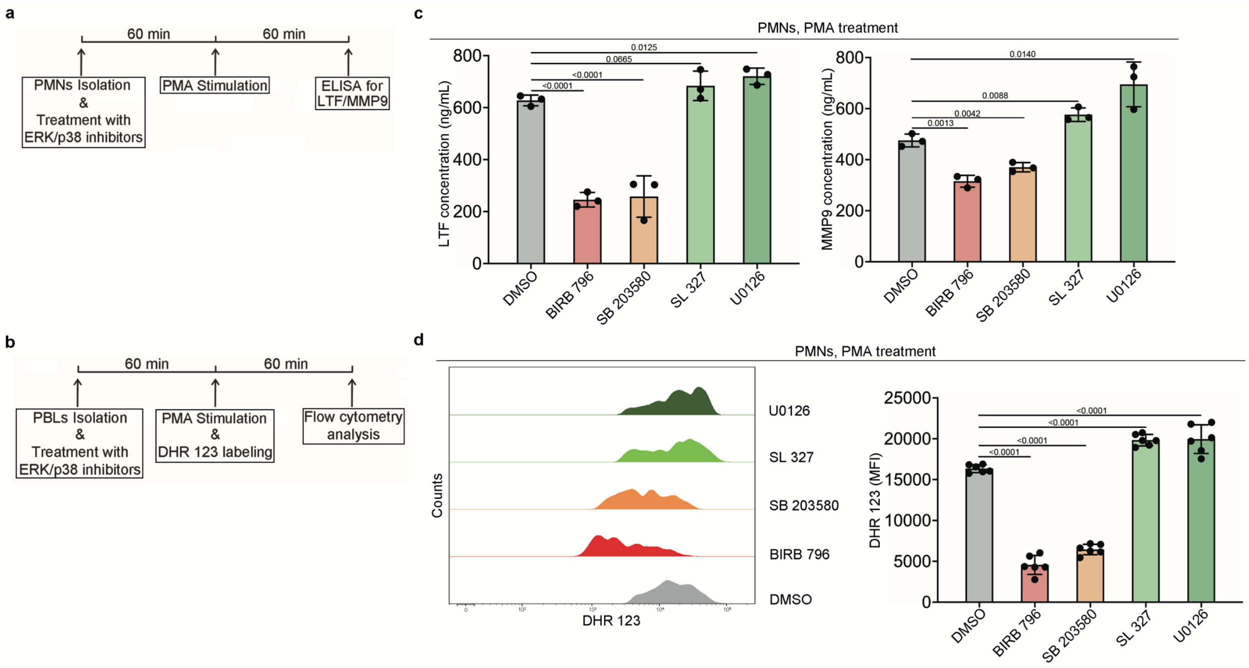

Supplementary Figure 14. Pre-inhibition of p-p38 but not pERK decreases ROS release and degranulation of neutrophils. (a-b) Protocols for the pre-treatment of resting peripheral neutrophils (PMNs) with ERK/p38 inhibitors and subsequent measurement of degranulation and ROS release after PMA stimulation. **(c)** ELISA analysis of LTF and MMP9 release in WT PMNs pre-treated with ERK/p38 inhibitors or DMSO (control). n = 3 biological independent samples/group. **(d)** DHR 123 staining, flow cytometry and corresponding quantitative analysis of ROS levels in WT PMNs pre-treated with ERK/p38 inhibitors or DMSO (control). n = 6 biological independent samples/group. All quantitative data shown in this figure are presented as mean values ± SD. One-way ANOVA with Tukey's multiple comparison test and Two-sided unpaired T-test were used to calculate the presented p-values. Source data of **c** and **d** are provided in a Source Data File. PMA, phorbol-12-myristate-13-acetate; LTF, lactoferrin; MMP9: matrix metallopeptidase 9; DHR 123: dihydrorhodamine 123.

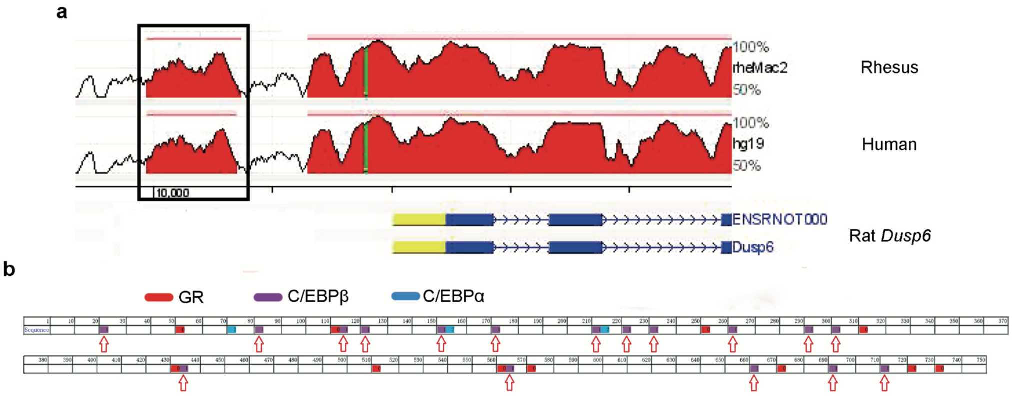

Supplementary Figure 15. In silico analysis of rat *Dusp6* gene promoter. (a) A conserved sequence (black box) in the *Dusp6* promoter region (from –2030 to –1284 bp) retrieved by sequence alignment (www.dcode.org). The heights of red peaks represent the degree of sequence homology in the genomic regions of human and rhesus compared with rats. **(b)** Computational prediction of C/EBPβ (purple with red arrows), C/EBPα (blue), and GR (red) binding sites in the conserved Dusp6 promoter fragment using PROMO.

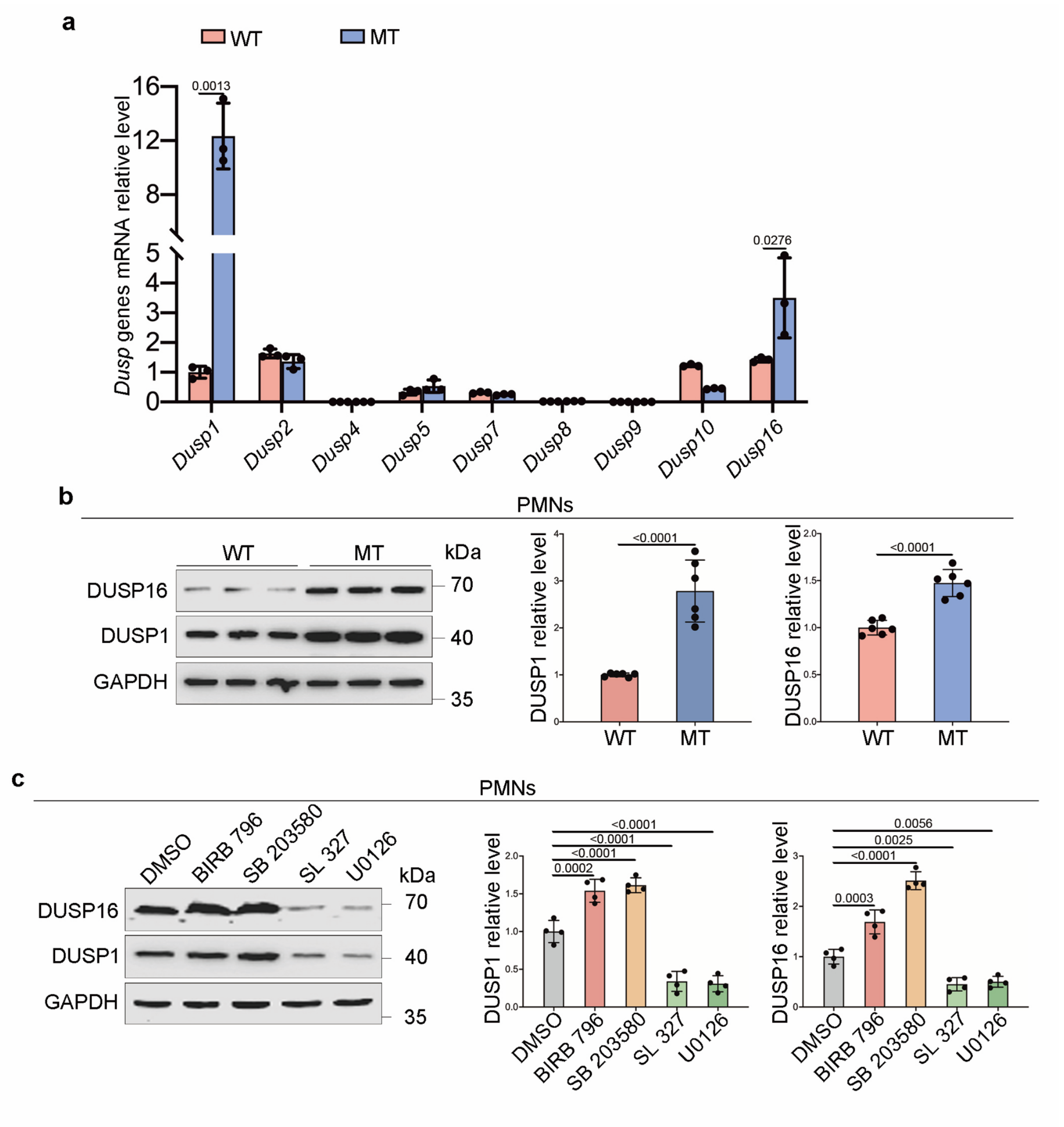

Supplementary Figure 16. *Dusp6* deficiency induces pERK-dependent DUSP1 and DUSP16 expression in resting PMNs. (a) Quantitative RT-PCR of *Dusp* family genes (other than *Dusp6*) in WT and *Dusp6*-deficient resting PMNs (n = 3 biological independent samples/group). **(b)** Western blots and quantitative analysis of DUSP1 and DUSP16 in WT and *Dusp6*-deficient PMNs (n = 6 biological independent samples/group). **(c)** Western blots and quantitative analysis of DUSP1 and DUSP16 in WT PMNs treated with either DMSO, p38 inhibitors (BIRB 796 or SB 203580), or MEK1/2 inhibitors (SL 327 or U0126). n = 4 biological independent samples/group. All

blots within each individual panel were performed in parallel with the same samples. All quantitative data shown in this figure are presented as mean values ± SD. One-way ANOVA with Tukey's multiple comparison test (for **c**) and Two-sided unpaired T-test (for **a** and **b**) were used to calculate the presented p-values. Source data of **a**, **b** and **c** are provided in a Source Data File. WT: wild type; MT: *Dusp6* mutant.

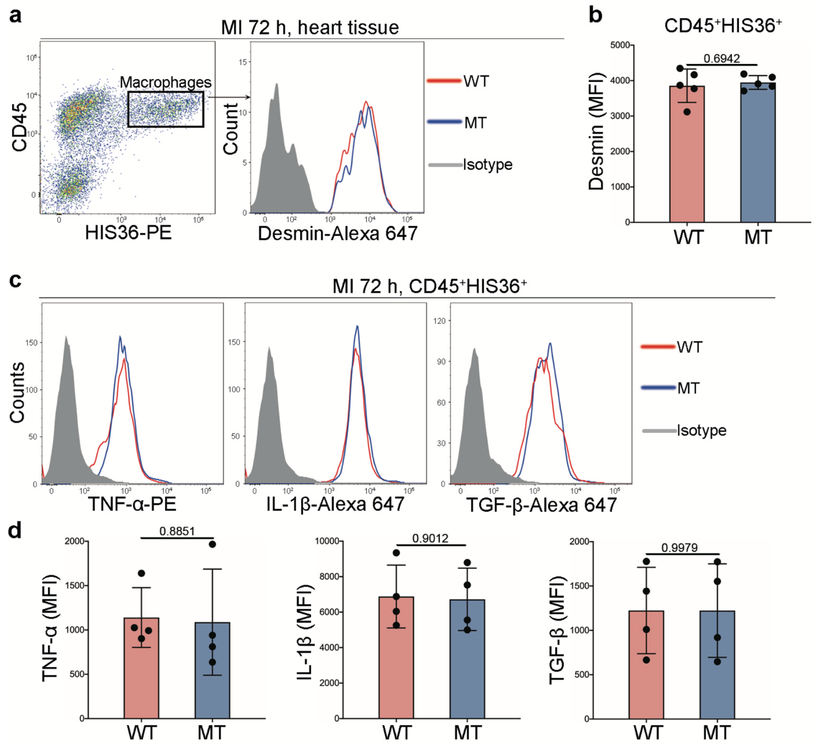

Supplementary Figure 17. ***Dusp6*** **deficiency has no effect on macrophage phagocytosis and cytokine production after MI. (a-b)** Intracellular staining, flow cytometry and corresponding quantitative analysis of the desmin level in CD45⁺HIS36⁺ macrophages from WT and *Dusp6*-deficient LV tissue at 72 h after MI (n = 5 biological independent samples/group). **(c-d)** Cell staining and flow cytometry with TNF-α, IL-1β and TGF-β antibody and corresponding quantitative analysis in WT and *Dusp6*-deficient CD45⁺HIS36⁺ macrophages existing in infarcted LV tissues at 72 h after MI (n = 5 biological independent samples/group). All quantitative data shown in this figure are presented as mean values ± SD. Two-sided unpaired T-test was used to calculate the presented p-values. Source data of **b** and **d** are provided in a Source Data File. WT: wild type; MT: *Dusp6* mutant.

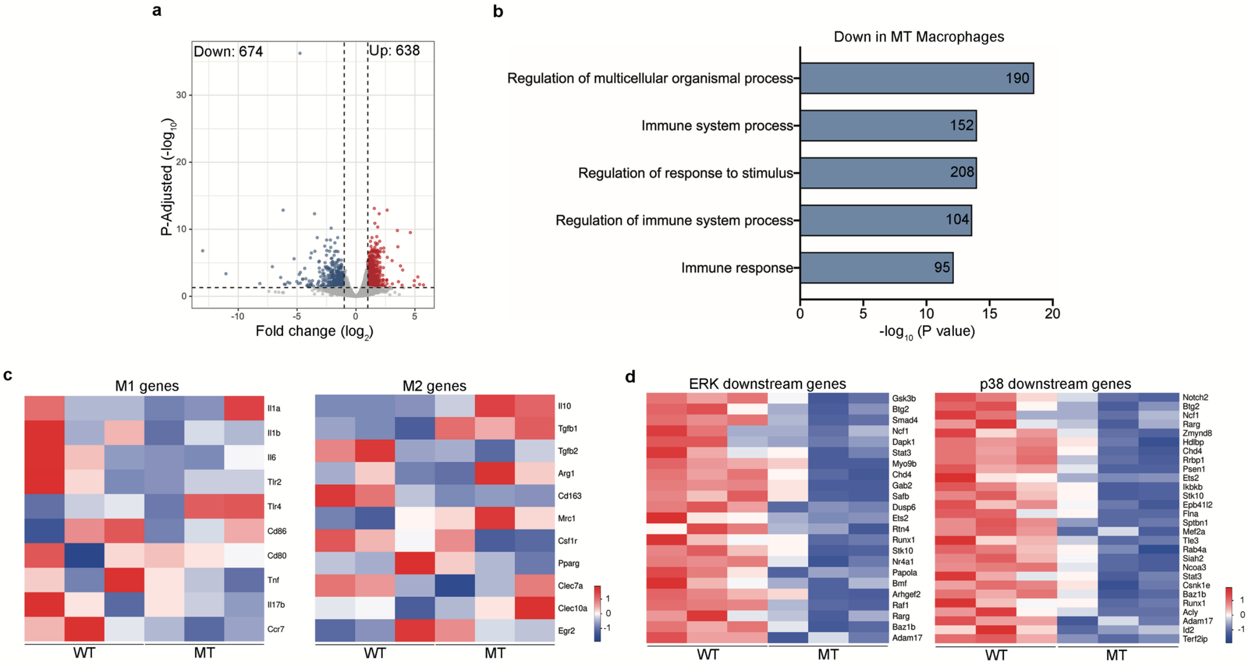

Supplementary Figure 18. Differentially expressed genes in WT and *Dusp6*-deficient macrophages at 72 h after MI. (a) Volcano plot depicting differentially expressed genes (DEGs) showing downregulation of 674 and upregulation of 638 genes, which were defined by a fold change ($\log_2$) > 1 and adjusted P value < 0.05, in *Dusp6*-deficient compared with WT cardiac macrophages at 72 h after MI. n = 3 biological independent samples/group. The p-values were attained by the Wald test and adjusted using the Benjamini and Hochberg's approach. **(b)** Gene ontology analysis of enriched biological processes in down-regulated genes in *Dusp6*-deficient macrophages. Number of genes belonging to each category is indicated. **(c-d)** Heatmaps illustrating mRNA expression fold changes of M1/M2 macrophage associated genes **(c)** and ERK/p38 downstream genes in Dusp6-deficient vs. WT cardiac macrophages at 72 h after MI. WT: wild type; MT: *Dusp6* mutant.

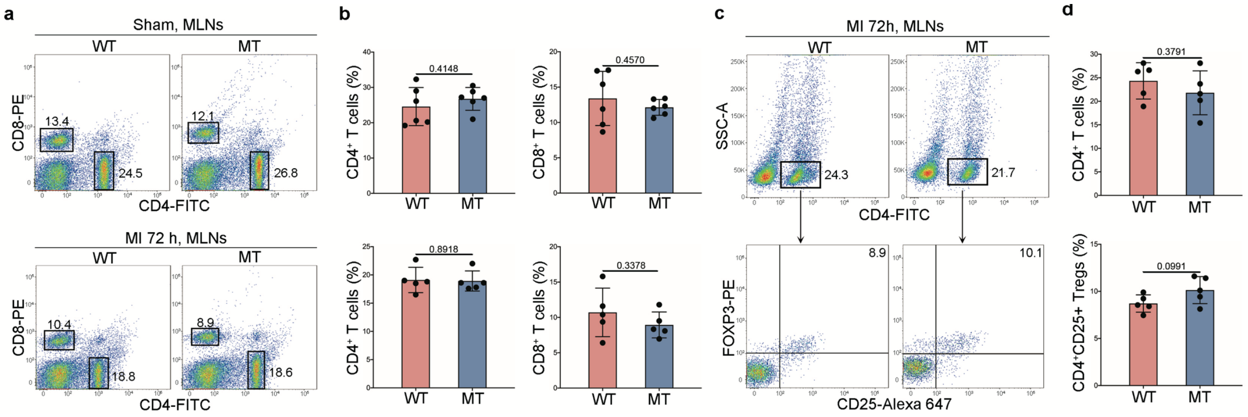

Supplementary Figure 19. ***Dusp6*** **deficiency has no effect on T cell development after MI. (a-b)** Cell staining and flow cytometry with CD4 and CD8 antibody and corresponding quantitative analysis of $CD4^+$ and $CD8^+$ T cell subsets from WT and Dusp6-deficient MLNs in sham-operated animals or at 72 h after MI. **(c-d)** Cell staining and flow cytometry with CD4, CD25 and FOXP3 antibody and corresponding quantitative analysis of $CD4^+CD25^+FOXP3^+$ Treg cells from WT and Dusp6-deficient mediastinal lymph nodes at 72 h after MI. n = 5 biological independent samples/group. All quantitative data shown in this figure are presented as mean values ± SD. Two-sided unpaired T-test was used to calculate p-values. Source data of **b** and **d** are provided in a Source Data File. WT: wild type; MT: *Dusp6* mutant; MLNs: mediastinal lymph nodes.

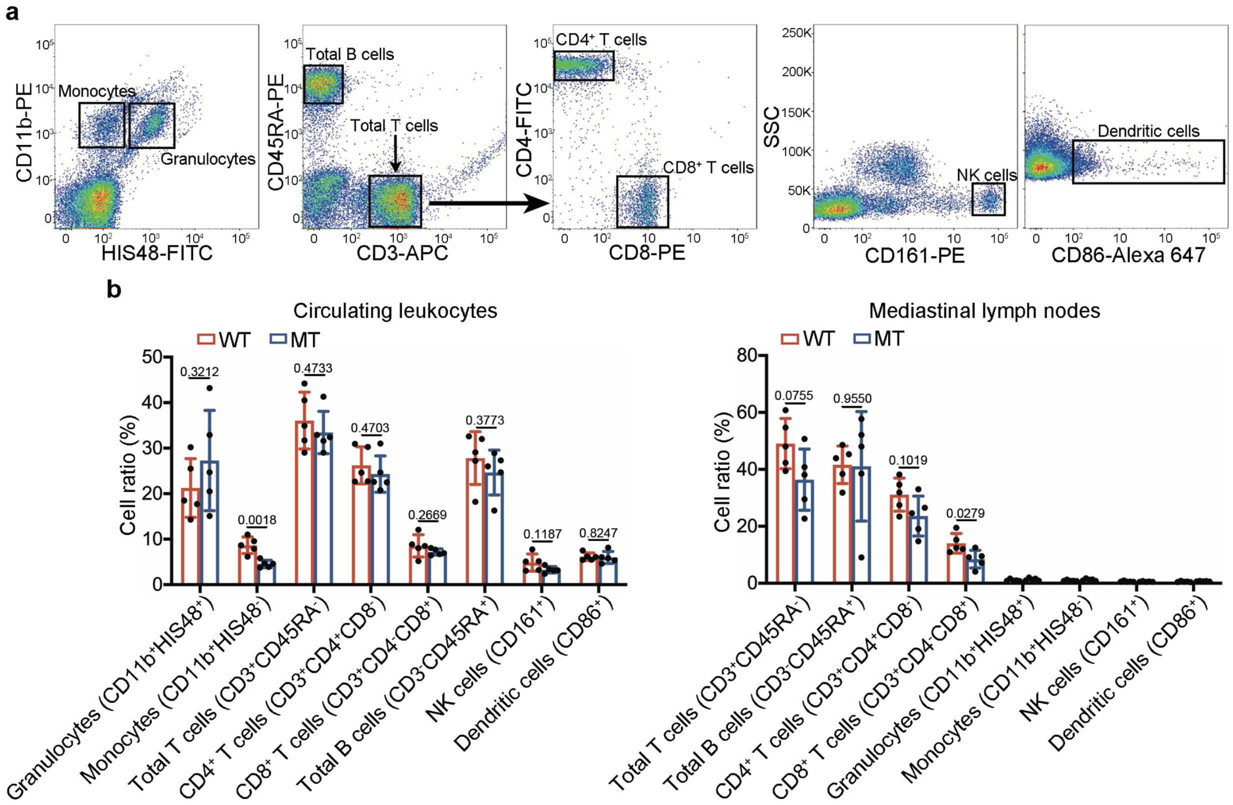

Supplementary Figure 20. Percentage of multiple immune cell populations in peripheral leukocytes and mediastinal lymph nodes of WT and *Dusp6*-deficient rats. (a) Representative gating images of flow cytometry analysis for immune cell populations using myeloid cell marker CD11b, granulocyte marker HIS48, T cell marker CD3/CD4/CD8, B cell marker CD45RA, NK cell marker CD161, and dendritic cell marker CD86. **(b)** Quantitative results for the flow cytometry analysis of granulocytes, monocytes, total T cells, $CD4^+$ T cells, $CD8^+$ T cells, total B cells, NK cells, and dendritic cells in WT and *Dusp6*-deficient circulating leukocytes and mediastinal lymph nodes. n = 5 biological independent samples/group. All quantitative data shown in this figure are presented as mean values ± SD. Two-sided unpaired T-test was used to calculate the

presented p-values. Source data of **b** are provided in a Source Data File. WT: wild type; MT: *Dusp6* mutant.

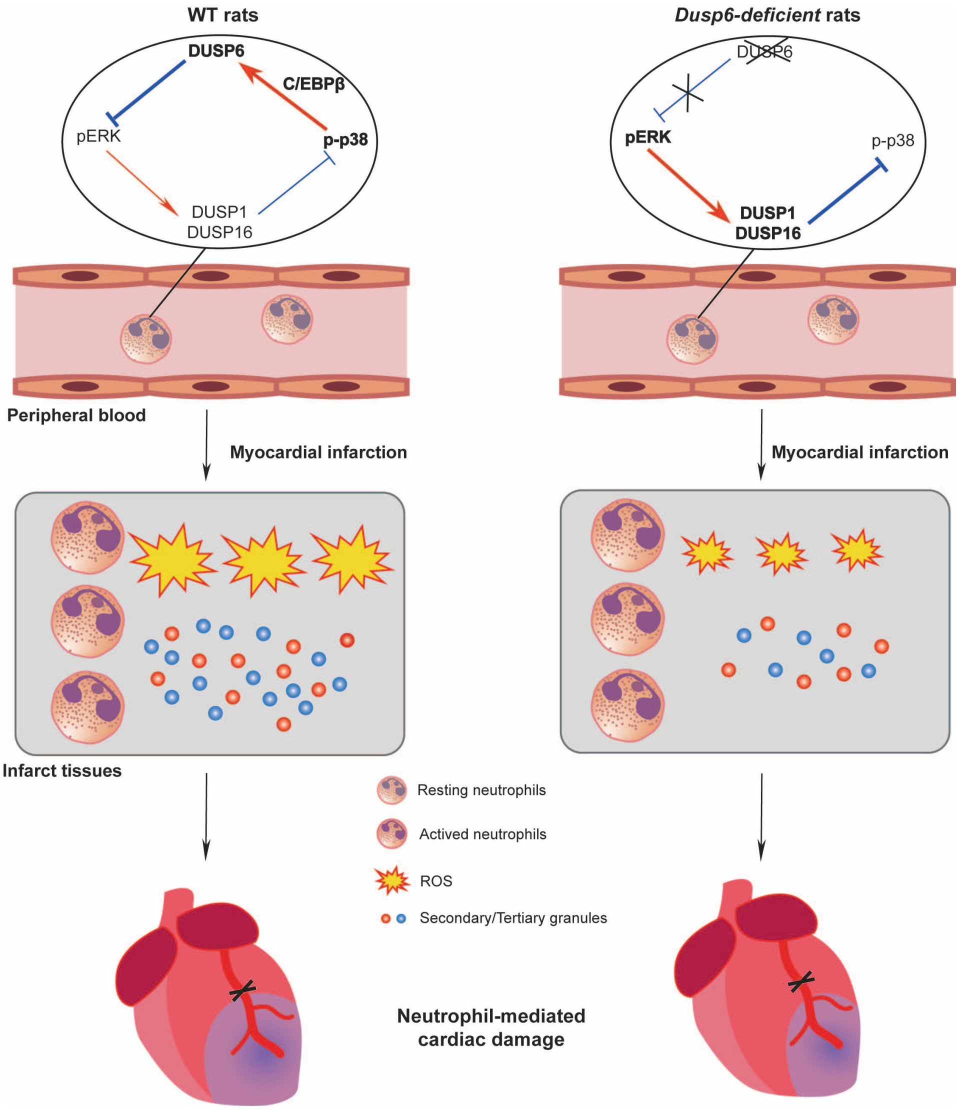

Supplementary Figure 21. Working model of the role of DUSP6 in neutrophil-mediated cardiac damage. DUSP6 is a critical effector for maintaining a high level of p-p38 MAPK by dephosphorylating pERK in resting neutrophils, where *Dusp6* itself is transcriptionally regulated by p-p38-C/EBPβ and the two p38 phosphatases DUSP1 and DUSP16 are regulated by pERK. *Dusp6* deficiency leads to elevated pERK and thus induces the expression of DUSP1 and DUSP16 to inhibit p-p38 activity, resulting in less ROS and secondary/tertiary granule release from neutrophils while having no effect on their maturation and chemotaxis-induced infiltration. Therefore, *Dusp6* deficiency

improves cardiac repair and function by attenuating neutrophil inflammation and cardiac damage at the inflammatory stage post-MI.

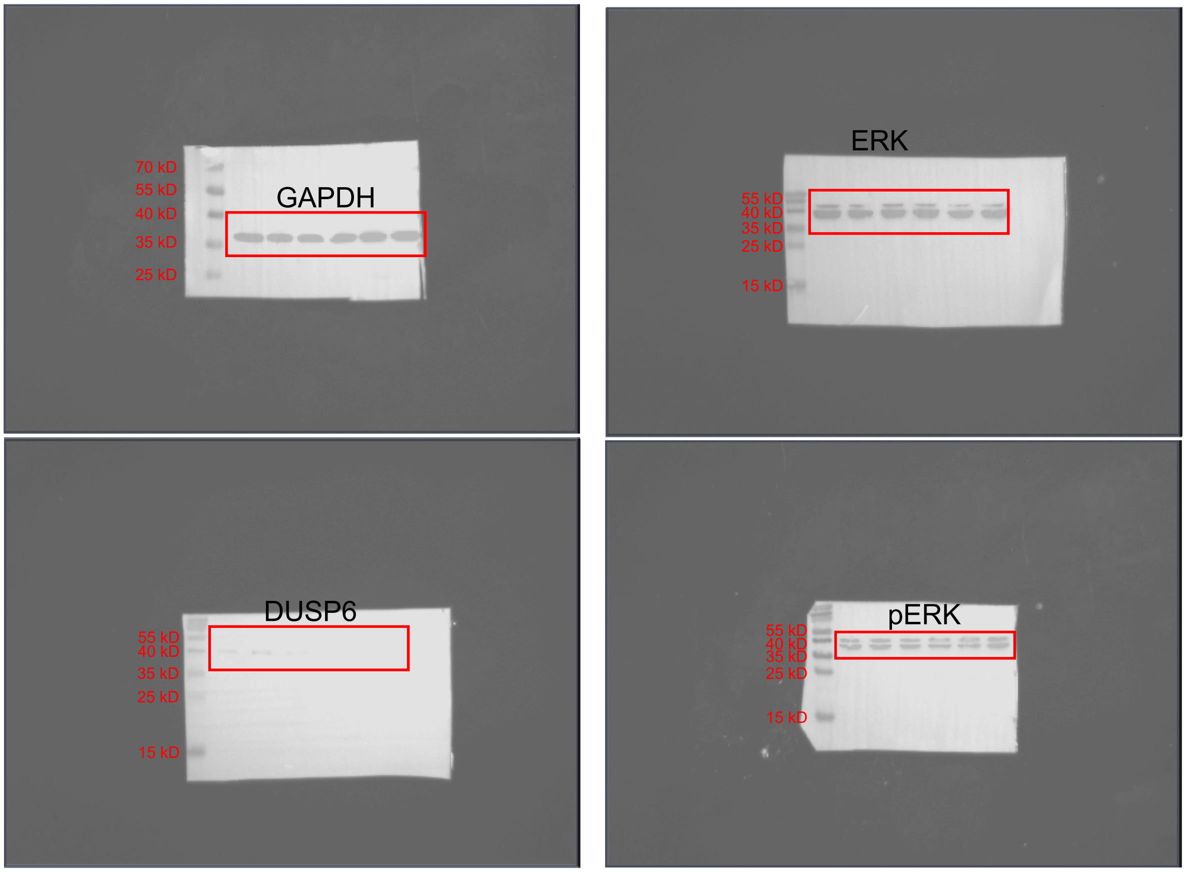

Supplementary Figure 22. Uncropped blots shown in Supplementary Figure 1b.

Indicated boxes show the corresponding cropped blots.

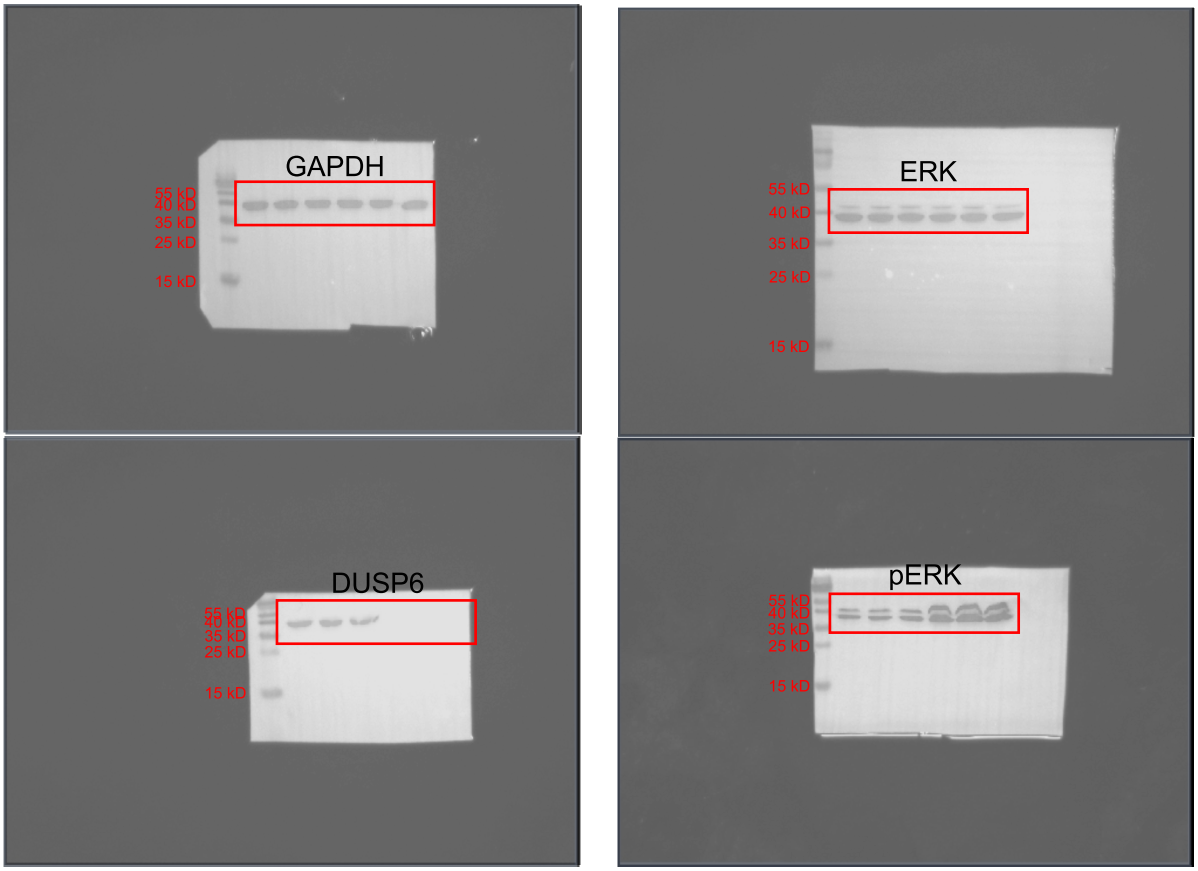

Supplementary Figure 23. Uncropped blots shown in Supplementary Figure 1d.

Indicated boxes show the corresponding cropped blots.

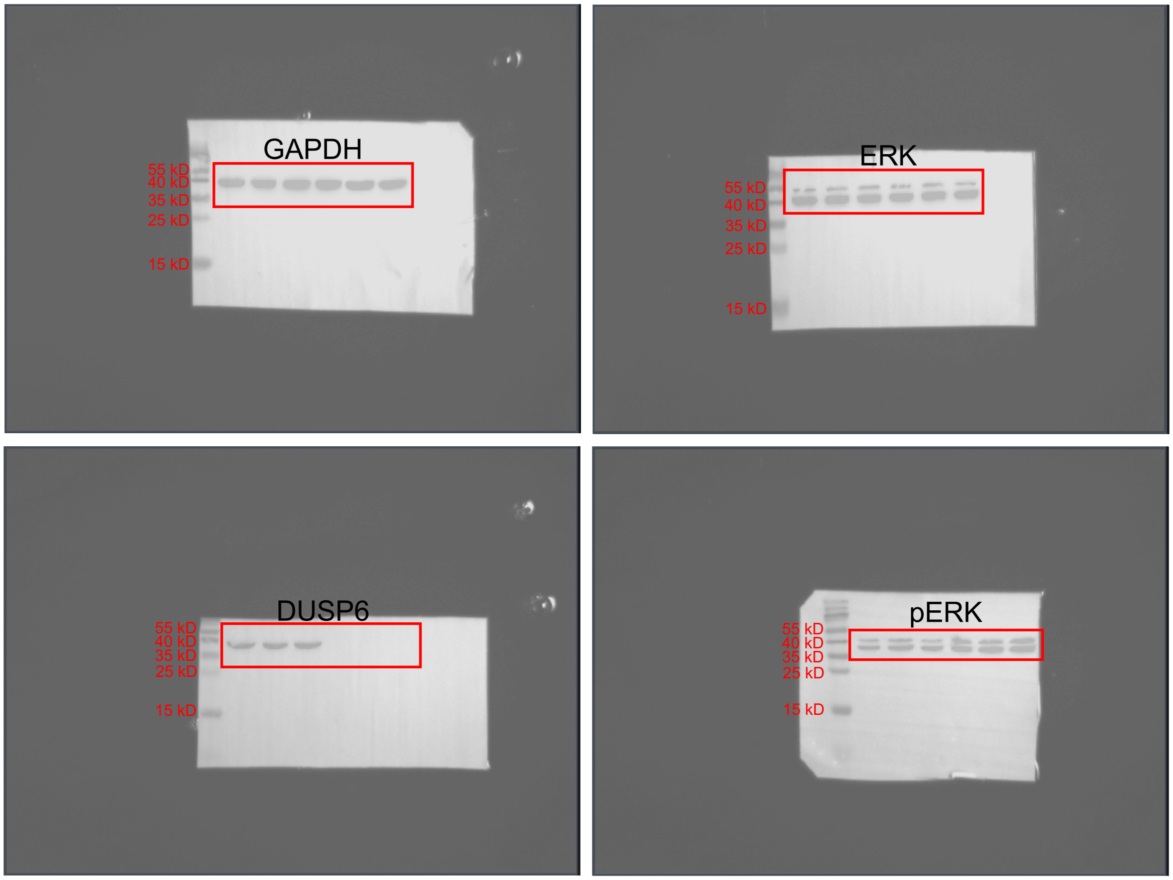

Supplementary Figure 24. Uncropped blots shown in Supplementary Figure 1f.

Indicated boxes show the corresponding cropped blots.

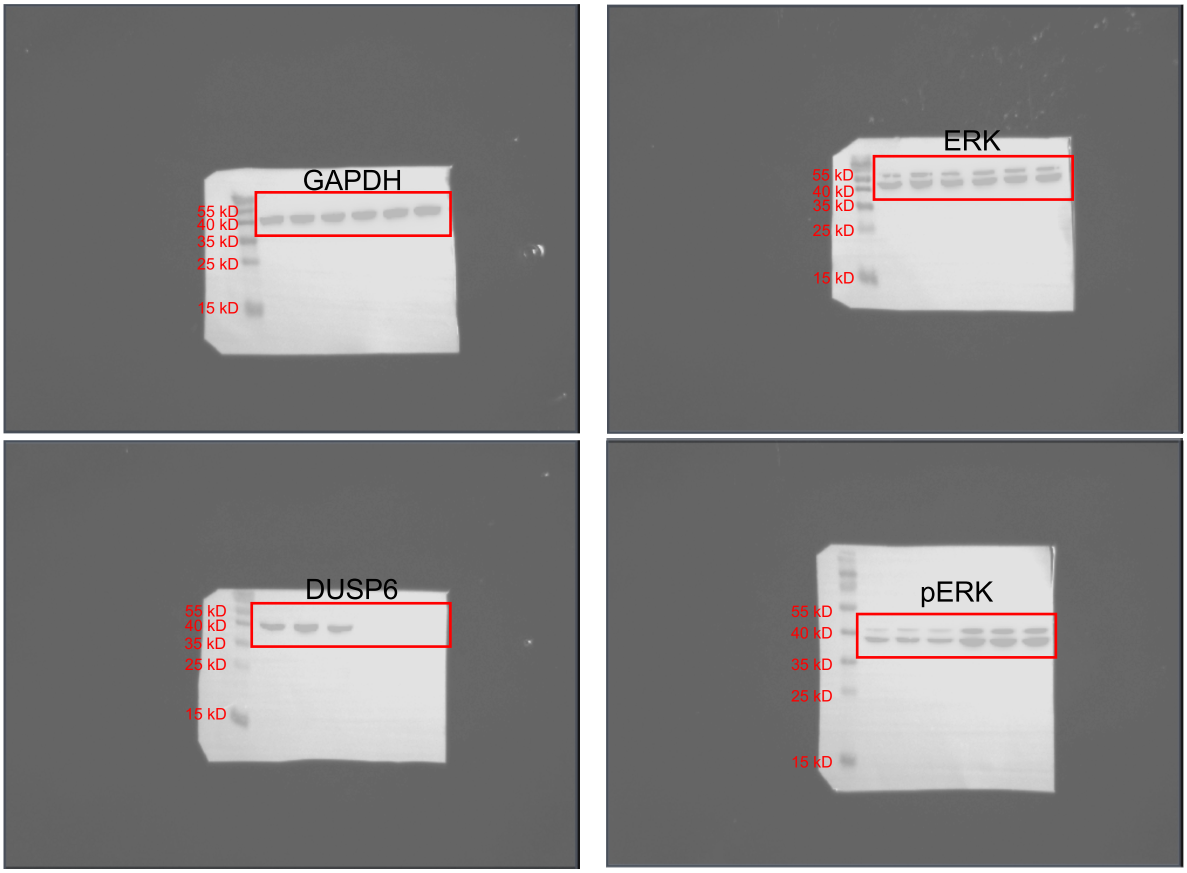

Supplementary Figure 25. Uncropped blots shown in Supplementary Figure 1h.

Indicated boxes show the corresponding cropped blots.

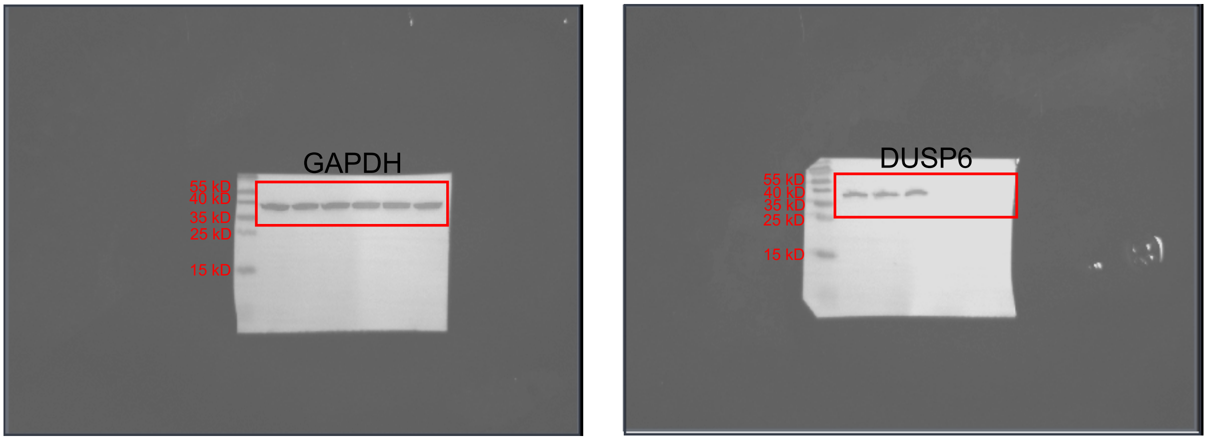

Supplementary Figure 26. Uncropped blots shown in Supplementary Figure 11b. Indicated boxes show the corresponding cropped blots.

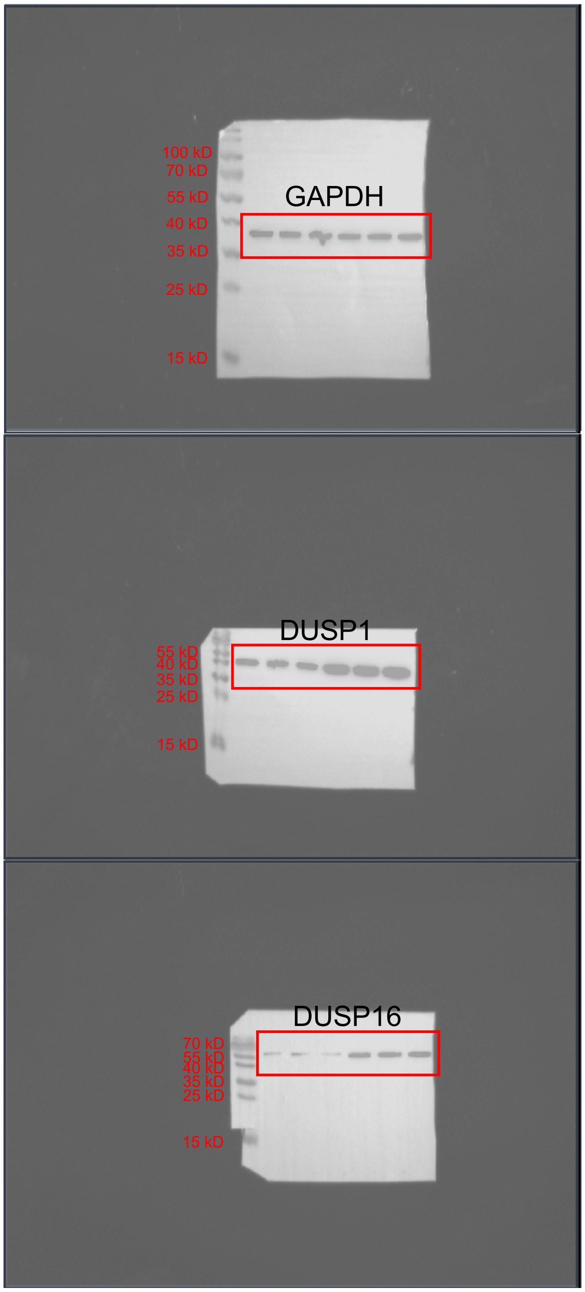

Supplementary Figure 27. Uncropped blots shown in Supplementary Figure 16b. Indicated boxes show the corresponding cropped blots.

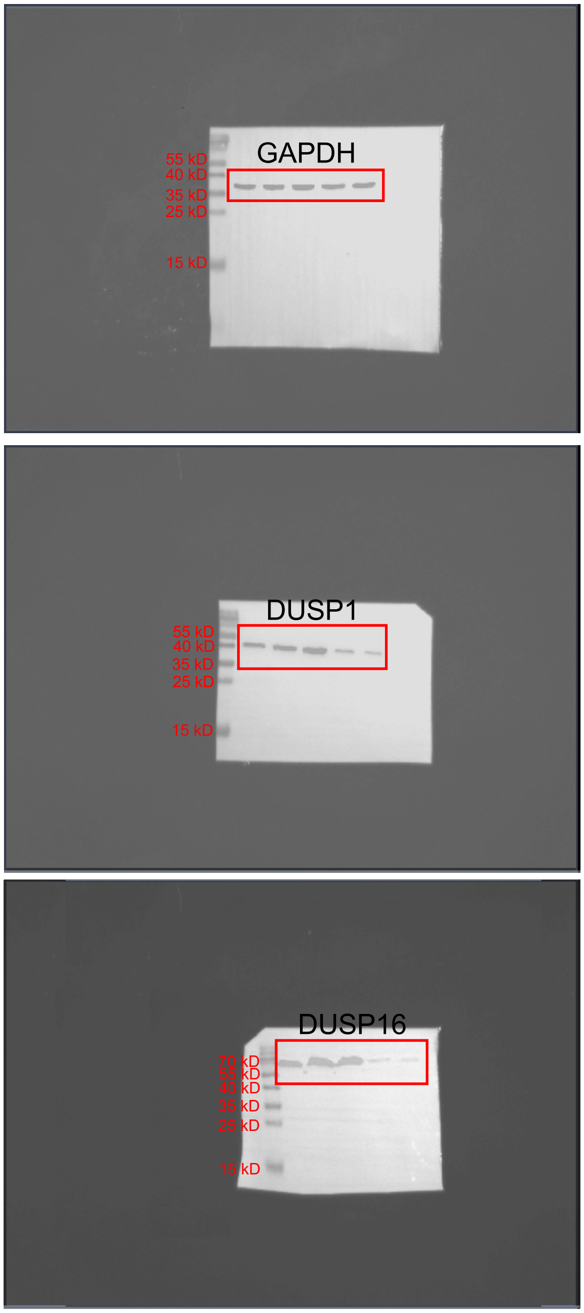

Supplementary Figure 28. Uncropped blots shown in Supplementary Figure 16c.

Indicated boxes show the corresponding cropped blots.

Supplementary Tables

Supplementary Table I. Primer sequences for qRT-PCR and ChIP-PCR

Primer name	Sequence
Dusp1 (rat) forward	CTGCTTCTGGATTGTCGCTCC
Dusp1 (rat) reverse	GCTGCGTTCGTCCAGCAGCA
Dusp2 (rat) forward	GTGCACTGCCAAGCTGGTAT
Dusp2 (rat) reverse	GTGACACAGCACCTGGGTCT
Dusp4 (rat) forward	TGCCAGGCCGGCATCTCTAG
Dusp4 (rat) reverse	GCAGGACGTGGTGAGCACCT
Dusp5 (rat) forward	CATGCATCCAAGTGCGAGTT
Dusp5 (rat) reverse	GACACAGTCAATAAAATCTA
Dusp6 (rat) forward	GGAAATGGCGATCAGCAAGA
Dusp6 (rat) reverse	TCCTCGCAGCGCGTGAATAG
Dusp6 (rat) ChIP forward	CTAGCCAGCGAAATATG
Dusp6 (rat) ChIP reverse	CGACTTGGAGGTCCAAG
Dusp7 (rat) forward	CGCTCCAAGAAGTGCGGTGT
Dusp7 (rat) reverse	CTGGGTGTGTGGTTGTCGCA
Dusp8 (rat) forward	TGCTGAGCTCTGTGAACATCTGCT
Dusp8 (rat) reverse	ACTGTCGAAGCAGCCGTCCA
Dusp9 (rat) forward	ACTTGGTCAAGCGGAAGAAG
Dusp9 (rat) reverse	AAGGTGGGTCTGAGACAGTG
Dusp10 (rat) forward	CAAGCAGCTAGCCAGCATCAAA

Dusp10 (rat) reverse	ATCTTGCCCTGTTGCAGCCT
Dusp16 (rat) forward	CTCAACAAGGAGCTGATGCAACAG
Dusp16 (rat) reverse	GATCCCAGCCAAGCAGTGGA

Supplementary Table 2. Measurement of organ weights.

	WT (2-month old)	*Dusp6* mutant (2-month old)
Body weight (BW) (g)	287 ± 13.4	279 ± 17.9
Liver weight/BW (mg/g)	37 ± 3.2	41 ± 1.8 $^{(p=0.0753)}$
Spleen weight/BW (mg/g)	2.4 ± 0.1	4.2 ± 0.5 $^{(p<0.0001)}$
Lung weight/BW (mg/g)	4.2 ± 0.3	4.4 ± 0.2 $^{(p=0.4963)}$
Heart weight/BW (mg/g)	2.8 ± 0.1	3.2 ± 0.2 $^{(p=0.0020)}$
Kidney weight/BW (mg/g)	7.8 ± 0.5	7.2 ± 0.4 $^{(p=0.0601)}$

Values are mean ± SD; n = 6 rats/group; Two-sided unpaired T-test.

Supplementary Table 3. Heart physiological data.

		EF (%)	FS (%)	LVEDV (mm^3)	LVESV (mm^3)
Pre-surgery	WT	69.79±5.44	39.80±3.50	216.3±45.3	69.35±18.35
	Dusp6 mutant	71.61±7.08	40.57±6.66	193.0±35.6	57.55±15.39
	P-value	0.9810	0.9888	0.7855	0.9214
MI 4 w	WT	33.07±11.38	16.91±6.72	390.2±70.0	242.4±81.0
	Dusp6 mutant	54.26±8.33	29.73±6.15	320.5±83.5	139.2±45.5
	P-value	<0.0001	<0.0001	0.0366	<0.0001

Values are mean ± SD. WT, n = 11; *Dusp6* mutant, n = 13. One-way ANOVA with Tukey's multiple comparison test. EF, ejection fraction; FS, fractional shortening; LVEDV, left ventricular end-diastolic volume; LVESV, left ventricular end-systolic volume.
