# Supplementary material for: Dusp6 deficiency attenuates neutrophil-mediated cardiac damage in the acute inflammatory phase of myocardial infarction
Source: Nat Commun. 2022 Nov 5;13:6672. doi: 10.1038/s41467-022-33631-z (PMC9637103; doi:10.1038/s41467-022-33631-z)
Supplement: Supplementary file 2 — Reporting Summary [file 41467_2022_33631_MOESM2_ESM.pdf]

nature portfolio

Corresponding author(s): Jingwei Xiong; XiaojunZhu

Last updated by author(s): Sep 26, 2022

Reporting Summary

Nature Portfolio wishes to improve the reproducibility of the work that we publish. This form provides structure for consistency and transparency in reporting. For further information on Nature Portfolio policies, see our Editorial Policies and the Editorial Policy Checklist.

Statistics

For all statistical analyses, confirm that the following items are present in the figure legend, table legend, main text, or Methods section.

n/a	Confirmed	
☐	☒	The exact sample size (*n*) for each experimental group/condition, given as a discrete number and unit of measurement
☐	☒	A statement on whether measurements were taken from distinct samples or whether the same sample was measured repeatedly
☐	☒	The statistical test(s) used AND whether they are one- or two-sided<br>*Only common tests should be described solely by name; describe more complex techniques in the Methods section.*
☒	☐	A description of all covariates tested
☐	☒	A description of any assumptions or corrections, such as tests of normality and adjustment for multiple comparisons
☐	☒	A full description of the statistical parameters including central tendency (e.g. means) or other basic estimates (e.g. regression coefficient) AND variation (e.g. standard deviation) or associated estimates of uncertainty (e.g. confidence intervals)
☐	☒	For null hypothesis testing, the test statistic (e.g. *F*, *t*, *r*) with confidence intervals, effect sizes, degrees of freedom and *P* value noted<br>*Give P values as exact values whenever suitable.*
☒	☐	For Bayesian analysis, information on the choice of priors and Markov chain Monte Carlo settings
☒	☐	For hierarchical and complex designs, identification of the appropriate level for tests and full reporting of outcomes
☒	☐	Estimates of effect sizes (e.g. Cohen's *d*, Pearson's *r*), indicating how they were calculated

Our web collection on statistics for biologists contains articles on many of the points above.

Software and code

Policy information about availability of computer code

Data collection	ZEN 3 for confocal imaging; Quantity One for the imaging of western blot and agarose gel electrophoresis; StepOne 2.3 for quantitative RT-PCR analysis; BD FACSuite for flow cytometry analysis.
Data analysis	Image-Pro Plus and Image J were used to analyze the results of Masson's staining, TTC-Evans blue staining, immunostaining, and Western blot. Flowjo 10 was used to analyze the results of Flow cytometry. RNA-seq data analysis was performed using HISAT2, featureCounts and DEseq2 packages. The generation of graphs, as well as the determination of significance of differences were performed with Graphpad Prism 8.

For manuscripts utilizing custom algorithms or software that are central to the research but not yet described in published literature, software must be made available to editors and reviewers. We strongly encourage code deposition in a community repository (e.g. GitHub). See the Nature Portfolio guidelines for submitting code & software for further information.

Data

Policy information about availability of data

All manuscripts must include a data availability statement. This statement should provide the following information, where applicable:

- Accession codes, unique identifiers, or web links for publicly available datasets
- A description of any restrictions on data availability
- For clinical datasets or third party data, please ensure that the statement adheres to our policy

The data for the expression levels of DUSP6 RNA in multiple human cell types were obtained frrom Human Protein Atlas (https://www.proteinatlas.org/ENSG00000139318-DUSP6/single+cell+type). The RNA-seq datasets of cardiac macrophages generated in this study have been deposited in Gene Expression

Omnibus (GEO) under accession code GSE195978. The remaining data in this study are available within the article, Supplementary Information and source data file. Source data are provided with this paper.

Human research participants

Policy information about studies involving human research participants and Sex and Gender in Research.

Reporting on sex and gender	*Use the terms sex (biological attribute) and gender (shaped by social and cultural circumstances) carefully in order to avoid confusing both terms. Indicate if findings apply to only one sex or gender; describe whether sex and gender were considered in study design whether sex and/or gender was determined based on self-reporting or assigned and methods used. Provide in the source data disaggregated sex and gender data where this information has been collected, and consent has been obtained for sharing of individual-level data; provide overall numbers in this Reporting Summary. Please state if this information has not been collected. Report sex- and gender-based analyses where performed, justify reasons for lack of sex- and gender-based analysis.*
Population characteristics	*Describe the covariate-relevant population characteristics of the human research participants (e.g. age, genotypic information, past and current diagnosis and treatment categories). If you filled out the behavioural & social sciences study design questions and have nothing to add here, write "See above."*
Recruitment	*Describe how participants were recruited. Outline any potential self-selection bias or other biases that may be present and how these are likely to impact results.*
Ethics oversight	*Identify the organization(s) that approved the study protocol.*

Note that full information on the approval of the study protocol must also be provided in the manuscript.

Field-specific reporting

Please select the one below that is the best fit for your research. If you are not sure, read the appropriate sections before making your selection.

☒ Life sciences ☐ Behavioural & social sciences ☐ Ecological, evolutionary & environmental sciences

For a reference copy of the document with all sections, see nature.com/documents/nr-reporting-summary-flat.pdf

Life sciences study design

All studies must disclose on these points even when the disclosure is negative.

Sample size	For experiments involving qRT-PCR, western blot, immunostaining, ELISA, flow cytometry and RNA-seq analyses, n = 3 was chosen as the minimal replicate number according to the typically minimum standards in the field, shown in previous studies (PMID: 19570514, 23261783, 32130914 and 32076644). Nonetheless, we performed most of these experiments with the sample size no less than 4 for each group. For all animal experiments including MI surgery and echocardiography, as well as the assessments of survival rate and infarct size, we used n = 5 as the minimal replicate number but only for the analyses in Supplementary Figure 12c-d. For other animal experiments in this study, we made the sample size as large as possible, which was determined by the specific situation of animal feeding and availability, to ensure the reliability of the results.
Data exclusions	No data exclusion was executed in our study.
Replication	We have repeated all presented experiments were at least three times with biological independent samples, and got similar results.
Randomization	All animals chosen for MI surgery and the subsequent analyses were sex- and age-matched, and were operated and analyzed equally and randomly.
Blinding	The operator of all echocardiographic analyses was blinded to group allocation during data collection and analysis. For other experiments, blinding was not possible since the primary investigators performed the experiments from the beginning to the end due to the technical nature of the experiments.

Reporting for specific materials, systems and methods

We require information from authors about some types of materials, experimental systems and methods used in many studies. Here, indicate whether each material, system or method listed is relevant to your study. If you are not sure if a list item applies to your research, read the appropriate section before selecting a response.

Materials & experimental systems

n/a	Involved in the study
☐	☒ Antibodies
☐	☒ Eukaryotic cell lines
☒	☐ Palaeontology and archaeology
☐	☒ Animals and other organisms
☒	☐ Clinical data
☒	☐ Dual use research of concern

Methods

n/a	Involved in the study
☒	☐ ChIP-seq
☐	☒ Flow cytometry
☒	☐ MRI-based neuroimaging

Antibodies

Antibodies used

anti-DUSP6 (1:100, Origene, TA323084, lot #C509AA0011), anti-HIS48 (1:50, clone HIS48, Abd Serotec, MCA967), anti-MPO (1:50, clone 8F4, Novus, NBP1-51148, lot #1619M1114A), anti-protease 3 (1:50, clone D-1, Santa Cruz, sc-74534, lot #C0414), anti-8-OHdG (1:100, clone N45.1, Abcam, ab48508, lot #GR3216151-2), anti-rat CD45-PE-Cy7 (1:300, clone OX-1, BD Biosciences, 561588, lot #9336582), anti-rat granulocyte-FITC (1:300, clone HIS48, BD Biosciences, 554907, lot #5190755), anti-rat macrophage-PE (1:300, clone HIS36, eBioscience, 12-0660-82,lot #4312190), anti-rat CD11b-PE (1:300, clone WT.5, BD Biosciences, 562105, lot #6183867), anti-rat CD31-FITC (1:200, clone TLD-3A12, Abd Serotec, MCA1334F, lot #1602), anti-rat CD3-APC (1:300, clone 1F4, BD Biosciences, 557030, lot #5149589), anti-rat CD4-PE (1:300, clone OX-35, BD Biosciences, 561833, lot #5099845), anti-rat CD8a-FITC (1:300, clone OX-8, BD Biosciences, 559976, lot #5121613), anti-rat CD25-Alexa Flour 647 (1:300, clone OX-39, Bio-Rad, MCA273A647, lot #150668), anti-rat CD45RA-PE (1:300, clone OX-33, BD Biosciences, 551402, lot #6036851), anti-rat CD161-PE (1:300, clone 10/78, BD Biosciences, 555009, lot #5176831), anti-rat CD86-Alexa Fluor 647 (1:300, clone 24F, Abd Serotec, MCA2874A647, lot #150664), anti-DUSP6 (1:200, clone EPR129Y, Abcam, ab76310, lot #GR3276783-3), anti-cTnT (1:200, clone 1C11, Abcam, ab8295), anti-α-SMA-FITC (1:200, clone 1A4, Abcam, ab184675), anti-desmin (1:200, clone Y66, Abcam, ab32362), anti-TNF-α-PE (1:300, clone TN3-19.12, BD Biosciences, 559503, lot #4323635), anti-IL1β (1:200, Abcam, ab9722, lot #GR3208882-20), anti-TGF-β (1:300, R&D, AB-100-NA, lot #EO2018111), anti-rat FOXP3-PE (1:300, clone FJK-16s, Invitrogen, 12-5773-80, lot #4232635), anti-GAPDH (1:5000, Easybio, BE0023), anti-β-actin (1:5000, Easybio, BE0021), anti-ERK (1:5000, clone 137F5, CST, 4695, lot #21), anti-pERK (1:1000, clone D13.14.4E, CST, 4370, lot #30), anti-p38 (1:5000, clone D13E1, CST, 8690, lot #8), anti-p-p38 (1:1000, clone D3F9, CST, 4511, lot #13), anti-JNK (1:1000, clone EPR16797-211, Abcam, ab179461, lot #GR31876062), anti-pJNK (1:500, clone EP1597Y, Abcam, ab76572, Lot #3323833-2), anti-BAX (1:1000, CST, 2772), anti-BCL-2 (1:1000, CST, 2870), anti-DUSP1 (1:1000, Millipore, 07-935, lot #2918976), anti-DUSP16 (1:1000, Biorbyt, orb215305, lot #DF3918), Alexa Fluor 488 goat anti-rabbit IgG (1:1000, Thermo Fisher, A32731), Alexa Fluor 555 goat anti-mouse IgG (1:1000, Thermo Fisher, A21422), Alexa Fluor 555 goat anti-mouse IgM (1:1000, Thermo Fisher, A21426), and Alexa Fluor 555 goat anti-rat IgG (1:1000, Thermo Fisher, A21434), Alexa Fluor 647 donkey anti-rabbit IgG (1:1000, Thermo Fisher, A31573), and Alexa Fluor 488 goat anti-mouse IgG (1:1000, Thermo Fisher, A11029), Goat anti-rabbit IgG–HRP (1:5000, Easybio, BE0101, lot #80790730) and goat anti-mouse IgG–HRP (1:5000, Easybio, BE0102, lot #80910305).

Validation

The specificity of anti-DUSP6 (ab76310) for western blot and flow cytometry was validated using samples from Dusp6-deficient rats and Dusp6 neutrophil-specific KO mice.

The specificity of anti-DUSP6 (TA323084) for immunohistochemistry was validated with samples from Dusp6-deficient rats.

All other antibodies were validated for the given species and application, either directly by the manufacturers or in the primary references cited and visible on the websites of the products:

anti-HIS48 (Abd Serotec, MCA967): https://www.bio-rad-antibodies.com/monoclonal/rat-granulocytes-antibody-his48-mca967.html?f=s%2Fn

anti-MPO (Novus, NBP1-51148): https://www.novusbio.com/products/myeloperoxidase-mpo-antibody-8f4_nbp1-51148

anti-protease 3 (Santa Cruz, sc-74534): https://www.scbt.com/p/pr3-antibody-d-1

anti-8-OHdG (Abcam, ab48508): https://www.abcam.com/8-hydroxy-2-deoxyguanosine-antibody-n451-ab48508.html

anti-rat CD45-PE-Cy7 (BD Biosciences, 561588): https://www.bdbiosciences.com/en-au/products/reagents/flow-cytometry-reagents/research-reagents/single-color-antibodies-ruo/pe-cy-7-mouse-anti-rat-cd45.561588

anti-rat granulocyte-FITC (BD Biosciences, 554907): https://www.bdbiosciences.com/en-us/products/reagents/flow-cytometry-reagents/research-reagents/single-color-antibodies-ruo/fitc-mouse-anti-rat-granulocytes.554907

anti-rat macrophage-PE (eBioscience, 12-0660-82): https://www.thermofisher.com/antibody/product/Mature-Macrophage-Marker-Antibody-clone-HIS36-Monoclonal/12-0660-82

anti-rat CD11b-PE (BD Biosciences, 562105): https://www.bdbiosciences.com/en-us/products/reagents/flow-cytometry-reagents/research-reagents/single-color-antibodies-ruo/pe-mouse-anti-rat-cd11b.562105

anti-rat CD31-FITC (Abd Serotec, MCA1334F): https://www.bio-rad-antibodies.com/monoclonal/rat-cd31-antibody-tld-3a12-mca1334.html?f=purified&utm_source=citeab.com&utm_medium=referral&utm_campaign=3rd+party+directory

anti-rat CD3-APC (BD Biosciences, 557030): https://www.bdbiosciences.com/en-us/products/reagents/flow-cytometry-reagents/research-reagents/single-color-antibodies-ruo/apc-mouse-anti-rat-cd3.557030

anti-rat CD4-PE (BD Biosciences, 561833): https://www.bdbiosciences.com/en-us/products/reagents/flow-cytometry-reagents/research-reagents/single-color-antibodies-ruo/fitc-mouse-anti-rat-cd4.561833

anti-rat CD8a-FITC (BD Biosciences, 559976): https://www.bdbiosciences.com/en-eu/products/reagents/flow-cytometry-reagents/research-reagents/single-color-antibodies-ruo/pe-mouse-anti-rat-cd8a.559976

anti-rat CD25-Alexa Flour 647 (Bio-Rad, MCA273A647): https://www.bio-rad-antibodies.com/static/datasheets/mca27/rat-cd25-antibody-ox-39-mca273a647.pdf

anti-rat CD45RA-PE (BD Biosciences, 551402): https://www.bdbiosciences.com/en-eu/products/reagents/flow-cytometry-reagents/research-reagents/single-color-antibodies-ruo/pe-mouse-anti-rat-cd45ra.551402

anti-rat CD161-PE (BD Biosciences, 555009): https://www.bdbiosciences.com/en-us/products/reagents/flow-cytometry-reagents/research-reagents/single-color-antibodies-ruo/pe-mouse-anti-rat-cd161a.555009

anti-rat CD86-Alexa Fluor 647 (Abd Serotec, MCA2874A647): https://www.bio-rad-antibodies.com/monoclonal/rat-cd86-antibody-24f-mca2874.html?f=purified

anti-cTnT (Abcam, ab8295): https://www.abcam.com/cardiac-troponin-t-antibody-1c11-ab8295.html

anti-α-SMA-FITC (Abcam, ab184675): https://www.abcam.com/alexa-fluor-488-alpha-smooth-muscle-actin-antibody-1a4-ab184675.html

anti-desmin (Abcam, ab32362): https://www.abcam.com/desmin-antibody-y66-cytoskeleton-marker-ab32362.html

anti-TNF-α-PE (BD Biosciences, 559503): https://www.bdbiosciences.com/en-eu/products/reagents/flow-cytometry-reagents/research-reagents/single-color-antibodies-ruo/pe-hamster-anti-rat-mouse-tnf.559503
anti-IL1β (Abcam, ab9722): https://www.abcam.com/il-1-beta-antibody-ab9722.html

anti-TGF-β (R&D, AB-100-NA): https://www.rndsystems.com/products/tgf-beta-pan-specific-antibody_ab-100-na

anti-rat FOXP3-PE (Invitrogen, 12-5773-80): https://www.thermofisher.com/antibody/product/FOXP3-Antibody-clone-FJK-16s-Monoclonal/12-5773-82

anti-GAPDH (Easybio, BE0023): http://www.bioeasytech.com/product/2355.html?goods_id=4243

anti-β-actin (Easybio, BE0021): http://www.bioeasytech.com/product/2377.html?goods_id=4265

anti-ERK (CST, 4695): https://www.cellsignal.com/products/primary-antibodies/p44-42-mapk-erk1-2-137f5-rabbit-mab/4695

anti-pERK (CST, 4370): https://www.cellsignal.com/products/primary-antibodies/phospho-p44-42-mapk-erk1-2-thr202-tyr204-d13-14-4e-xp-rabbit-mab/4370

anti-p38 (CST, 8690): https://www.cellsignal.com/products/primary-antibodies/p38-mapk-d13e1-xp-rabbit-mab/8690

anti-p-p38 (CST, 4511): https://www.cellsignal.com/products/primary-antibodies/phospho-p38-mapk-thr180-tyr182-d3f9-xp-rabbit-mab/4511

anti-JNK (Abcam, ab179461): https://www.abcam.com/jnk1--jnk2--jnk3-antibody-epr16797-211-ab179461.html

anti-pJNK (Abcam, ab76572): https://www.abcam.com/nav/primary-antibodies/rabbit-monoclonal-antibodies/jnk1jnk2jnk3-phospho-y185--y185--y223-antibody-ep1597y-ab76572.html

anti-BAX (CST, 2772): https://www.cellsignal.com/products/primary-antibodies/bax-antibody/2772

anti-BCL-2 (CST, 2870): https://www.cellsignal.com/product/productDetail.jsp?productId=2870

anti-DUSP1 (Millipore, 07-935): https://www.emdmillipore.com/US/en/product/Anti-MKP1-Antibody,MM_NF-07-535?ReferrerURL=https%3A%2F%2Fwww.google.com%2F

anti-DUSP16 (Biorbyt, orb215305): https://www.biorbyt.com/dusp16-antibody-orb215305.html

anti-C/EBPβ antibody (Cell Signaling Technology, 3082): https://www.cellsignal.com/products/primary-antibodies/c-ebpbeta-antibody/3082

anti-Histone H3 antibody (Cell Signaling Technology, 4620): https://www.cellsignal.com/products/primary-antibodies/histone-h3-d2b12-xp-rabbit-mab-chip-formulated/4620

Eukaryotic cell lines

Policy information about cell lines and Sex and Gender in Research

Cell line source(s)	HEK 293T reserved in our laboratory
Authentication	The HEK 293T has been authenticated by STR analysis in our previous study (PMID: 27929112). We did not furtherly authenticate it in this study.
Mycoplasma contamination	Cell line was not tested for Mycoplasma but no indicaiton of contamination was observed.
Commonly misidentified lines (See ICLAC register)	No commonly misidentified line was used in this study.

Animals and other research organisms

Policy information about studies involving animals; ARRIVE guidelines recommended for reporting animal research, and Sex and Gender in Research

Laboratory animals	Male Dusp6 mutant and littermate control rats at 8-10 weeks old for MI surgery and other subsequent analyses; Male Mrp8-Cre mice and female Dusp6 floxed mice at 8-10 weeks old to generate Dusp6 neutrophil-specific KO mice; Dusp6 neutrophil-specific KO mice at 8-10 weeks old for MI surgery and other subsequent analysis. Animal room condition was stated in the section of "Method".
Wild animals	No wild animals were used in this study
Reporting on sex	Male mice and rats were used for MI surgery and subsequent analyses in terms ofprevious studies (PMID: 19570514, 23261783, 32130914 and 32076644) . Female animals were used for breeding.
Field-collected samples	No filed-collected samples were used in this study.
Ethics oversight	Both rats and mice were raised and handled with the animal protocol (IMM-XiongJW-4) approved by the Peking University Institutional Animal Care and Use Committee, which is fully accredited by the Association for Assessment and Accreditation of Laboratory Animal Care International

Note that full information on the approval of the study protocol must also be provided in the manuscript.

Flow Cytometry

Plots

Confirm that:

- [x] The axis labels state the marker and fluorochrome used (e.g. CD4-FITC).
- [x] The axis scales are clearly visible. Include numbers along axes only for bottom left plot of group (a 'group' is an analysis of identical markers).
- [x] All plots are contour plots with outliers or pseudocolor plots.
- [x] A numerical value for number of cells or percentage (with statistics) is provided.

Methodology

Sample preparation	Single-cell suspensions of infarcted cardiac tissues were obtained by digestion with Liberase DL (Roche, 5401160001) for cardiac cells, neutrophils, and macrophages; single-T cell suspensions were obtained by smashing mediastinal lymph nodes (MLNs) through 40 μm filters.Total peripheral leukocytes were isolated from blood samples by performing erythrocyte sedimentation and lysis.
Instrument	FACSVerseTM and LSRFortessaTM flow cytometers (BD Biosciences), MoFlo XDP Cell Sorter (Beckman Coulter).
Software	BD FACSuite and Flowjo 10
Cell population abundance	The macrophages in infarcted LV tissues were sorted as CD45/HIS36 double positive without examining the abundance. The purity of sorted cells was validated by RNA-seq results, which displayed a predominant expression of macrophage associated genes.
Gating strategy	Debris was removed by gating on the main cell population using the FSC/SSC gating. Positive threshold for each antibody staining was defined on the basis of negative control. Identical positivity threshold was applied to all samples within one analysis.

☒ Tick this box to confirm that a figure exemplifying the gating strategy is provided in the Supplementary Information.
